# Crystal structure of *Plasmodium vivax* macrophage migration inhibitory factor

**DOI:** 10.1107/S2053230X26003870

**Published:** 2026-05-05

**Authors:** Aryana Nair, Matthew Lin, Arav Srivastava, Lijun Liu, Anne Cooper, Kevin Battaile, Elizabeth Harmon, Peter J. Myler, Bart L. Staker, Scott Lovell, Graham Chakafana, Oluwatoyin A. Asojo

**Affiliations:** a The Highlands School, 1451 East Northgate Drive, Irving, TX75062, USA; b Roslyn High School, Roslyn Heights, NY11577, USA; c Grafton High School, 403 Grafton Drive, Yorktown, VA23692, USA; d Seattle Structural Genomics Center for Infectious Diseases, Seattle, Washington, USA; e Protein Structure and X-ray Crystallography Laboratory, 2034 Becker Drive, Lawrence, KS66047, USA; f NYX, New York Structural Biology Center Upton NY11973 USA; g Center for Global Infectious Disease Research Seattle Children’s Research Institute 307 Westlake Avenue North, Suite 500 Seattle WA98109 USA; h Chemistry and Biochemistry Hampton University 200 William R. Harvey Way Hampton VA23666 USA; i Biochemistry and Cell Biology, Dartmouth Geisel School of Medicine, One Medical Center Drive, Lebanon, NH03756, USA; University of Oxford, United Kingdom

**Keywords:** SSGCID, structural genomics, macrophage migration inhibitory factor, l-dopachrome isomerase, malaria vaccine candidate, cytokines

## Abstract

*Plasmodium vivax* is a major cause of malaria globally and has recently been transmitted locally in the USA. *P. vivax* produces homologs of host proteins, including cytokines such as macrophage migration inhibitory factor (MIF). MIF regulates both adaptive and innate immune responses and contributes to the pathogenesis of parasitic infections, including malaria. Plasma concentrations of *P. vivax* MIF (*Pv*MIF) correlate with the severity of *P. vivax* malaria. *Plasmodium* spp. MIFs have been recognized as candidate malaria vaccines. *Pv*MIF, like other protozoan MIFs, binds to host CD74 and can suppress host MIF–CD74 signaling. The production, crystallization and 1.8 Å resolution structure of *Pv*MIF (PDB entry 9b0m, pdb_00009b0m) are reported. *Pv*MIF crystallized in space group *P*6_3_ with a single molecule in the asymmetric unit. The biological unit of *Pv*MIF is the prototypical MIF trimer.

## Abstract 2

The production, crystallization and 1.8 Å resolution crystal structure of macrophage migratory inhibitory factor from *P. vivax* are reported.

## 1. Introduction

Over one-third of the world’s population are at risk from *Plasmodium vivax*, the most geographically expansive of the five human-infecting *Plasmodium* spp. (Battle *et al.*, 2019 ▸). The Southeast Asian and Western Pacific regions have a highest prevalence of *P. vivax* cases (Howes *et al.*, 2016 ▸; Weiss *et al.*, 2025 ▸). *P. vivax* was formerly endemic in North America and Europe, and was eradicated by the 1970s; however, in 2023 the first locally transmitted *P. vivax* malaria cases in two decades were reported (DeVita *et al.*, 2025 ▸). *Plasmodium* spp. are transmitted by bites of infected female *Anopheles* mosquitoes, and once in the human host, *P. vivax* infects hepatocytes (liver stage or pre-erythrocytic stage), eventually leading to blood-stage infection or the erythrocytic stage associated with typical malaria symptoms (Khan & Daily, 2022 ▸; Chu & White, 2021 ▸). *P. vivax* can persist in human hosts as hypnozoites in the liver, which can cause relapses that can extend over several months or years (Flannery *et al.*, 2022 ▸). The cyclical fever and weakness episodes of *P. vivax* malaria lead to high treatment costs and productivity loss in endemic countries (Baird *et al.*, 2016 ▸). Additionally, the dormant liver stage can cause relapse weeks, months or even decades after the first infection, complicating treatment of *P. vivax* malaria and requiring antimalarial drugs that are effective against both the blood and liver stages. Additionally, the main antimalarial with *P. vivax* activity, primaquine, is contraindicated in people with glucose-6-phosphate dehydrogenase (G6PD) deficiency (Douglas *et al.*, 2023 ▸). This is because primaquine induces hemolytic anemia in people with glucose-6-phosphate dehydrogenase (G6PD) deficiency, which corresponds to approximately 15% of the population living in *P. vivax* endemic regions (Douglas *et al.*, 2023 ▸).

The Seattle Structural Genomics Center for Infectious Diseases and collaborators are investigating *P. vivax* proteins for therapeutics discovery as well as to obtain mechanistic insights (Vijayan *et al.*, 2021 ▸; Mendez *et al.*, 2025 ▸; Bolling *et al.*, 2024 ▸). One of the proteins of interest is the *P. vivax* orthologue of human macrophage migration inhibitory factor (MIF). MIF is a cytokine that regulates adaptive and innate responses and has roles in the pathogenesis of parasitic infections, including malaria (Calandra & Roger, 2003 ▸). Protozoan parasite MIF homologs mimic their human MIF counterparts (hMIF1, NCBI Accession No. CAG30406.1, and hMIF2, NCBI Accession No. CAG30317.1), facilitating the modulation of host immune responses and suppressing apoptosis-induced cell death (Twu *et al.*, 2014 ▸; Ghosh *et al.*, 2019 ▸). Multiple structures of hMIF1 and hMIF2 have been determined and reveal biological trimers that bind to CD74 (Rajasekaran *et al.*, 2014 ▸; Meza-Romero *et al.*, 2016 ▸; Sun *et al.*, 1996 ▸). Plasma concentrations of *P. vivax* MIF (*Pv*MIF) correlated with parasitaemia and severity of the disease in *P. vivax* malaria patients (Han *et al.*, 2010 ▸). Furthermore, *Plasmodia* spp. MIFs have been recognized as candidate malaria vaccines that protect against severe malaria infection (Baeza Garcia *et al.*, 2018 ▸). Additionally, the suppression of MIF–CD74 signaling by *Plasmodia* spp. MIF was protective against severe malaria infection (Baeza Garcia *et al.*, 2021 ▸). As part of ongoing studies to clarify the structures and functions of *P. vivax* proteins that are therapeutic targets, we present here the cloning, purification, crystallization and structure of *Pv*MIF.

## 2. Materials and methods

### 2.1. Macromolecule production

Cloning, expression and purification followed standard SSGCID protocols, as described previously (Serbzhinskiy *et al.*, 2015 ▸; Kimble *et al.*, 2024 ▸; Srivastava *et al.*, 2024 ▸). Briefly, the full-length gene for putative macrophage migration inhibitory factor from *P. vivax* Salvador I (UniProt A5K093), encoding amino acids 1–116 (*Pv*MIF), was PCR-amplified from cDNA using the primers in Table 1 ▸. The gene was cloned into pET-28a, which encodes an N-terminal His-tag and adds 21 additional residues to the expressed protein. Chemical transformation of competent *Escherichia coli* BL21(DE3) Rosetta cells with the resulting plasmid was followed by small-scale expression tests. 2 l of culture was then grown using established SSGCID protocols.

His-*Pv*MIF was purified in a two-step protocol consisting of an immobilized metal (Ni^2+^) affinity chromatography (IMAC) step and size-exclusion chromatography (SEC) at 4°C. All chromatography runs were performed on an ÄKTApurifier 10 (GE Healthcare) using automated IMAC and SEC programs (Bryan *et al.*, 2011 ▸). Thawed bacterial pellets (∼25 g) were lysed by sonication in 200 ml lysis buffer [25 m*M* HEPES pH 7.0, 500 m*M* NaCl, 5%(*v*/*v*) glycerol, 0.5%(*w*/*v*) CHAPS, 30 m*M* imidazole, 10 m*M* MgCl_2_, 1 m*M* TCEP, 250 µg ml^−1^ AEBSF, 0.025%(*w*/*v*) sodium azide]. After sonication, the crude lysate was treated with 20 µl Benzonase (25 U µl^−1^) and incubated with mixing at room temperature for 45 min. The lysate was clarified by centrifugation at 11 850*g* for 1 h using a Sorvall centrifuge (Thermo Scientific). The clarified supernatant was then passed over an Ni–NTA HisTrap FF 5 ml column (GE Healthcare) which had been pre-equilibrated with loading buffer [25 m*M* HEPES pH 7.0, 500 m*M* NaCl, 5%(*v*/*v*) glycerol, 30 m*M* imidazole, 1 m*M* TCEP, 0.025%(*w*/*v*) sodium azide]. The column was washed with 20 column volumes (CV) of loading buffer and was eluted with loading buffer plus 250 m*M* imidazole in a linear gradient over 7 CV. Peak fractions were pooled and concentrated to 5 ml for SEC. A SEC column (Superdex 75, GE) was equilibrated with SEC running buffer [20 m*M* HEPES pH 7.0, 300 m*M* NaCl, 5%(*v*/*v*) glycerol, 1 m*M* TCEP]. The peak fractions were collected and analyzed for *Pv*MIF using SDS–PAGE. The protein eluted as a single, monodisperse peak of ∼19 kDa in SEC running buffer. Based on molecular standards, the expected monomer molecular weight is ∼15 kDa. The peak fraction was pooled and concentrated to 12 mg ml^−1^ using an Amicon purification system (Millipore). Aliquots of 110 µl were flash-frozen in liquid nitrogen and stored at −80°C until use. The expression clone (HepyC.00487.a.B1.GE40934) and recombinant protein (PlviB.00834.a) can be requested from SSGCID (https://www.ssgcid.org/available-materials).

### 2.2. Crystallization

His-*Pv*MIF crystals were grown by vapor diffusion directly from the Index (Hampton Research) crystallization screen condition as described in Table 2 ▸.

### 2.3. Data collection and processing

Data were collected at 100 K using a Dectris EIGER2 XE 9M detector on NSLS-II beamline 19-ID at Brookhaven National Laboratory (Table 3 ▸). Data were integrated with *XDS* (Kabsch, 2010 ▸) via *autoPROC* (Vonrhein *et al.*, 2024 ▸) and scaled with *AIMLESS* (Evans, 2011 ▸). Raw X-ray diffraction images have been stored at the Integrated Resource for Reproducibility in Macromolecular Crystallo­graphy at https://www.proteindiffraction.org.

### 2.4. Structure solution and refinement

The structure was determined by molecular replacement with *Phaser* (McCoy *et al.*, 2007 ▸) from the *CCP*4 suite of programs (Collaborative Computational Project, Number 4, 1994 ▸; Krissinel *et al.*, 2004 ▸; Winn *et al.*, 2011 ▸; Agirre *et al.*, 2023 ▸). The molecular-replacement search model was PDB entry 2wkf (Dobson *et al.*, 2009 ▸). As with other SSGCID structures, refinement involved iterative cycles in *Phenix* (Adams *et al.*, 2011 ▸; Liebschner *et al.*, 2019 ▸) followed by manual rebuilding of the structure using *Coot* (Emsley & Cowtan, 2004 ▸; Emsley *et al.*, 2010 ▸). The quality of the structure was checked with *MolProbity* (Williams *et al.*, 2018 ▸). Data-reduction and refinement statistics are shown in Table 4 ▸. Coordinates and structure factors have been deposited in the Worldwide PDB (wwPDB) as entry 9b0m (pdb_00009b0m).

## 3. Results and discussion

**Figure 1:**
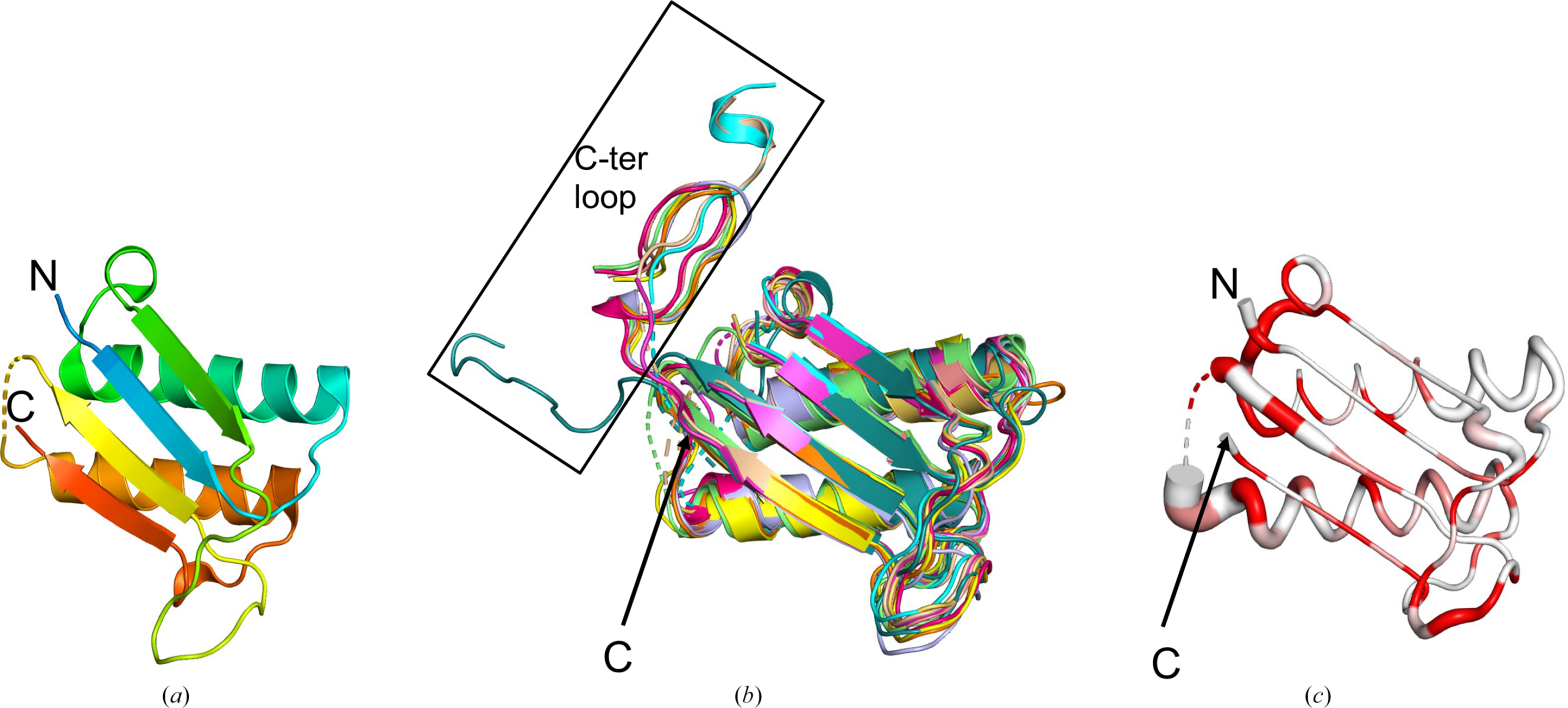
The *Pv*MIF monomer. (*a*) Cartoon representation of a *Pv*MIF monomer colored in rainbow from blue at the N-terminus to red at the C-terminus. (*b*) Superposed monomers of PDB entry 9b0m (gray) with PDB entries 9mf3 (lemon green), 2wkf (magenta), 4p7m (tan), 3gac (wheat), 2wkb (cyan), 4dh4 (slate blue), 8ap3 (orange), 8ur2 (green), 8vj2 (smudge green) and 2xcz (yellow), the orthologues identified by *ENDScript* that were used to generate the sausage plot (Dobson *et al.*, 2009 ▸; Pantouris *et al.*, 2014 ▸; Shao *et al.*, 2010 ▸; Sommerville *et al.*, 2013 ▸; Spiller *et al.*, 2023 ▸; Srivastava *et al.*, 2024 ▸; Kimble *et al.*, 2024 ▸; Wasiel *et al.*, 2010 ▸). (*c*) *Pv*MIF sausage plot generated by *ENDScript*. The ribbon (sausage) shows the relative conservation of secondary structure compared with the closest MIF structures. The ribbon thickness reflects the degree of secondary-structure similarity, with thinner ribbons indicating more conserved regions and thicker ribbons indicating lower conservation, as determined by r.m.s.d. alignment of the structures in (*c*). The ribbon is colored based on sequence conservation, with red indicating identical residues (*a*), (*b*) and (*c*) show the same view and orientation of *Pv*MIF. The C-terminus and N-terminus are indicated by C and N, respectively.

The recombinant protein screened for crystallization included 21 additional amino-acid residues at the N-terminus corresponding to the purification tag and cleavage site, resulting in a 137-amino-acid polypeptide (Table 1 ▸). The protein crystallized directly from the screen conditions, and the structure of His-*Pv*MIF was determined in space group *P*6_3_, with a single monomer in the asymmetric unit (Fig. 1 ▸*a*). Only 96 of the 137 amino acids had ordered electron density. The final refined model is missing 41 amino-acid residues corresponding to the 21 N-terminal vector-derived residues, a five-residue loop 66-LGGIN-70 and the C-terminal loop 101-DCSAQNFAFNGSLFG-115. This apo structure contains no biologically relevant ligands, as it was neither soaked nor co-crystallized with any such ligands. The secondary structure of *Pv*MIF was determined by *PDBSum* (https://www.ebi.ac.uk/thornton-srv/databases/pdbsum/) as 24% strand, 36.6% α-helix and 5.2% 3_10_-helix.

The most similar structures to *Pv*MIF were identified by *PDBeFold* (https://www.ebi.ac.uk/msd-srv/ssm/) analysis (Krissinel & Henrick, 2004 ▸) using default protein secondary-structure element thresholds of 70% (https://www.ebi.ac.uk/pdbe/sites/default/files/documents/service_tutorials/PDBeFold.pdf) as MIF structures (PDB entries 2wkf, 4p7m and 4p7s) from *P. falciparum* (*Pf*MIF) with an r.m.s.d. of ∼0.7 Å (Dobson *et al.*, 2009 ▸; Pantouris *et al.*, 2014 ▸). This is unsurprising since *Pf*MIF shares ∼66% sequence identity with *Pv*MIF. The next most similar structure is that of a plant MIF-like protein (PDB entry 8dq6), with an r.m.s.d of ∼1.1 Å but only ∼23% identity to *Pv*MIF (Spiller *et al.*, 2023 ▸). Apart from the plant MIF-like protein, the most similar structures include those of a MIF from another *Plasmodium* spp., *P. berghei*, which shares ∼64% sequence identity with *Pv*MIF. Structures of MIFs from the rodent parasite *P. yoelii* (*Py*MIF; PDB entries 3gac and 3gad) with an r.m.s.d. of ∼0.7 Å and sharing ∼63% identity with *Pv*MIF (Shao *et al.*, 2010 ▸) ranked next in similarity (a summary of the outcome of *PDBeFold* is listed in Supplementary Table S1).

**Figure 2:**
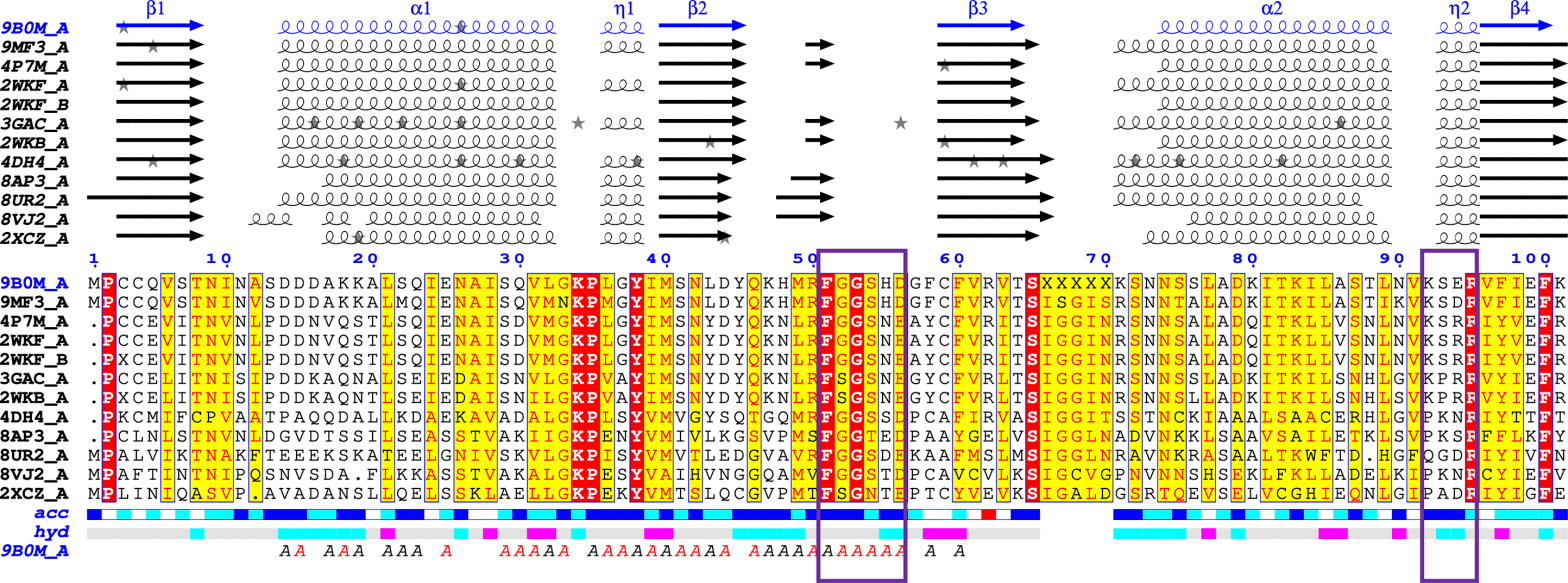
*ENDScript* with a *BLAST+* search performed against the PDBAA database and *BLAST+* hits aligned using *ClustalO* reveals the nearest structural neighbors of *Pv*MIF. Identical and conserved residues are highlighted in red and yellow, respectively. The different secondary-structure elements shown are α-helices (α), 3_10_-helices (η), β-strands (β) and β-turns (TT) (Gouet *et al.*, 1999 ▸, 2003 ▸). Regions corresponding to the MIF1 residues implicated in CD74 binding are shown in purple boxes.

Similar results were obtained with *ENDScript* using a *BLAST+* search performed against the PDBAA database and *BLAST+* hits aligned using *ClustalO* (Gouet *et al.*, 2003 ▸; Robert & Gouet, 2014 ▸). *ENDScript* revealed that the structure of *Pv*MIF is most similar to those from other *Plasmodium *spp., including *P. knowlesi* (PDB entry 9mf3; Seattle Structural Genomics Center for Infectious Diseases, unpublished work), *P. falciparum* (PDB entries 2wkf and 4p7m; Dobson *et al.*, 2009 ▸; Pantouris *et al.*, 2014 ▸), *P. yoelii* (PDB entry 3gac; Shao *et al.*, 2010 ▸) and *P. berghei* (PDB entry 2wkb; Dobson *et al.*, 2009 ▸). The next most similar structures are the MIFs from the protozoan parasite *Toxoplasma gondii* (PDB entry 4dh4; Sommerville *et al.*, 2013 ▸) and a plant MIF-like protein (PDB entry 8ap3; Spiller *et al.*, 2023 ▸). These are followed by the structures of *Trichomonas vaginalis* MIF (PDB entry 8ur2; Srivastava *et al.*, 2024 ▸), *Onchocerca volvulus* MIF (PDB entry 8vj2; Kimble *et al.*, 2024 ▸) and *Prochlorococcus marinus* MIF (PDB entry 2xcz; Wasiel *et al.*, 2010 ▸). Comparison of the superposed MIF structures reveals that the missing C-terminal residues in *Pv*MIF correspond to a loop (Fig. 1 ▸*b*). An *ENDScript*-generated sausage plot reveals that *Pv*MIF is a prototypical MIF, with greatest structural variation in the carboxyl-termini, as indicated by the ribbon thickness in the sausage plot (Fig. 1 ▸*c*). The regions with identical amino-acid residues are shown in red; notably, the N-terminal proline at the tautomerase site is conserved across all structures. Sequence-based secondary alignment also reveals the alignment of secondary-structure elements across all MIF structures, highlighting variations in strand, helix and loop lengths, especially at the carboxyl-termini (Fig. 2 ▸).

**Figure 3:**
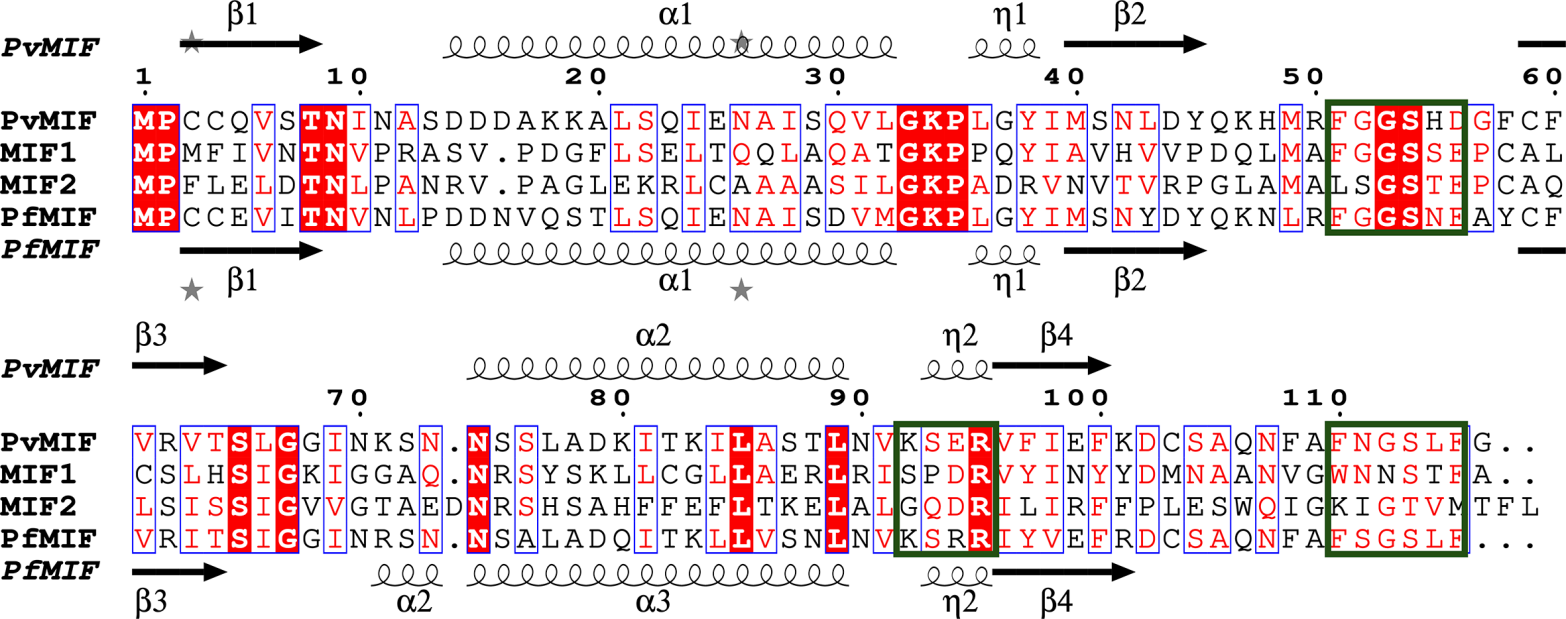
*ESPript* structure-based alignment with *Clustal Omega* sequence alignment of *Pv*MIF (PDB entry 9b0m), human MIF1 (PDB entry 1mif), human MIF2 (PDB entry 1ddt) and *Pf*MIF (PDB entry 2wkf). Human MIF residues implicated in CD74 binding are indicated with green boxes. The secondary-structure elements are as follows: α-helices are shown as large coils, 3_10_-helices are shown as small coils labeled h, β-strands are shown as arrows labeled β and β-turns are labeled TT. The identical residues are shown in a white font on a red background, conserved residues in a red font and conserved regions in blue boxes. The sequence alignment was performed using *ClustalW* and was followed by *ESPript* structure alignment.

CD74 binding by parasite MIFs facilitates their suppression of human MIF–CD74 signaling (Baeza Garcia *et al.*, 2021 ▸; Leng *et al.*, 2003 ▸). *ESPript* analysis with *Clustal Omega* alignment of *Pv*MIF with *Pf*MIF and human MIF1 and MIF2 enables the comparison of residues implicated in CD74 binding (Fig. 3 ▸). CD74 binding requires the formation of the prototypical MIF trimer (Leng *et al.*, 2003 ▸; Meza-Romero *et al.*, 2016 ▸). Experimental and docking studies of human MIF1 (hMIF1) implicate chain *A* residues 50-FGGSEP-55, K76, 90-SPDR-93 and 109-NNS-111 and chain *B* residues 34-PQ-35, 108-WNN-110 and 111-STFA-114 in CD74 binding (Meza-Romero *et al.*, 2016 ▸). Only five of the MIF1 residues implicated in CD74 binding are conserved in both *Pv*MIF and *Pf*MIF. The five residues are Phe50, Gly51, Gly52, Ser53 and Arg94 in MIF1, which correspond to Phe51, Gly52, Gly53, Ser54 and Arg95 in *Pv*MIF and *Pf*MIF (Fig. 3 ▸).

**Figure 4:**
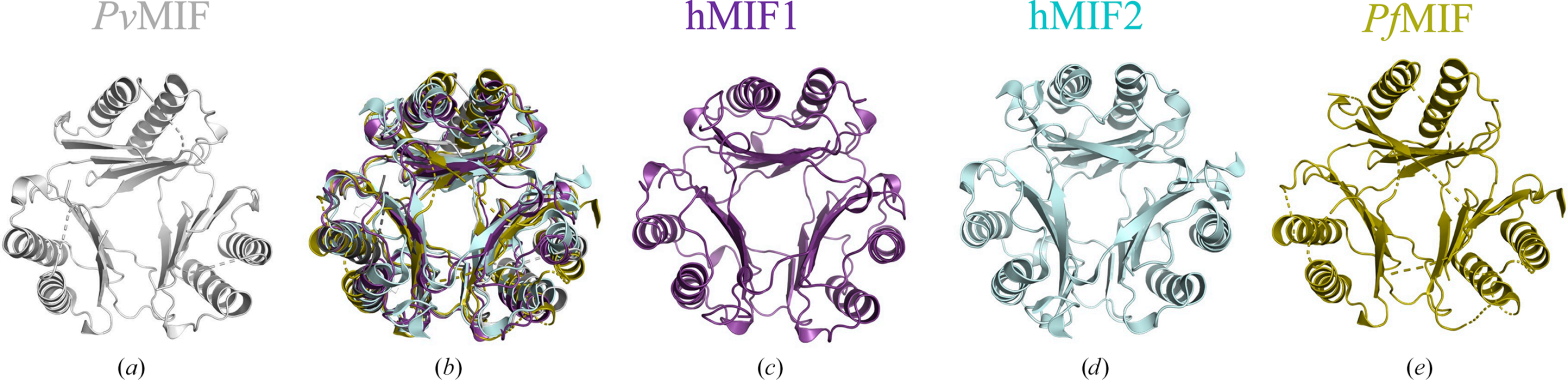
(*a*) Cartoon representation of the biological unit of *Pv*MIF (gray) reveals a prototypical MIF trimer. (*b*) Superposed trimers of *Pv*MIF (PDB entry 9b0m, gray) with human MIF1 (hMIF1; PDB entry 1mif, purple), human MIF2 (hMIF2; PDB entry 3ker, cyan) and *Pf*MIF (PDB entry 4p7m, yellow). The prototypical MIF trimers of (*c*) hMIF1 (PDB entry 1mif, purple; Sun *et al.*, 1996 ▸), (*d*) hMIF2 (PDB entry 3ker, cyan; Rajasekaran *et al.*, 2014 ▸) and (*e*) *Pf*MIF (PDB entry 4p7m) are shown. Proteins were aligned with *PyMOL* (DeLano, 2002 ▸).

The protomer of *Pv*MIF forms the prototypical MIF trimer with two symmetry mates (Fig. 4 ▸). The *Pv*MIF trimer aligns well with hMIF1 (r.m.s.d. of ∼1.1 Å for 210 residues), MIF2 (r.m.s.d. of ∼11.3 Å for 213 residues) and *Pf*MIF (r.m.s.d. of ∼10.5 Å for 240 residues) as measured by *PyMOL*. Additionally, the *Pv*MIF trimer retains a similar interface and packing to hMIF (Sun *et al.*, 1996 ▸) and hMIF2 (Rajasekaran *et al.*, 2014 ▸), thereby allowing the loop regions and residues implicated in CD74 binding to remain exposed.

## 4. Conclusion

*Pv*MIF shares primary-, secondary-, tertiary- and quaternary-structural features with human MIFs. Five of the human MIF1 amino-acid residues implicated in CD74 binding are conserved in *Pv*MIF. Additional studies are required to determine how *Pv*MIF binds CD74.

## Supplementary Material

PDB reference: *P. vivax* MIF, 9b0m

Supplementary Table S1. DOI: 10.1107/S2053230X26003870/ada5001sup1.pdf

## Figures and Tables

**Table 1:** Macromolecule-production information

Source organism	*Plasmodium vivax* Salvador I
DNA source	Wes Van Voorhis, UW PPG
Forward primer	5′-ATGCCCTGCTGTCAGGTTAGCA-3′
Reverse primer	5′-CCCAAACAGAGAGCCATTAAACG-3′
Expression vector	pET-28a, AVA N-terminal tag
Expression host	*E. coli* BL21(DE3) Rosetta
Complete amino-acid sequence of the construct produced†	**MAHHHHHHMGTLEAQTQGPGS**MPCCQVSTNINASDDDAKKALSQIENAISQVLGKPLGYIMSNLDYQKHMRFGGSHDGFCFVRVTSLGGINKSNNSSLADKITKILASTLNVKSERVFIEFKDCSAQNFAFNGSLFG

† Additional vector-derived N-terminal residues are underlined and in bold.

**Table 2:** Crystallization

Method	Vapor diffusion, sitting drop
Plate type	96-well compact Rigaku
Temperature (K)	291
Protein concentration (mg ml^−1^)	12
Buffer composition of protein solution	20 m*M* HEPES pH 7.0, 300 m*M* NaCl, 5%(*v*/*v*) glycerol, 1 m*M* TCEP
Composition of reservoir solution	25%(*w*/*v*) polyethylene glycol 3350, 0.1 *M* sodium acetate trihydrate pH 4.5
Volume and ratio of drop	0.2 µl:0.2 µl
Volume of reservoir (µl)	40
Cryosolution	20%(*w*/*v*) polyethylene glycol 3350, 0.08 *M* sodium acetate trihydrate pH 4.5, 20%(*v*/*v*) glycerol

**Table 3:** Data collection and processing Values in parentheses are for the outer shell.

Diffraction source	Beamline 19-ID, NSLS-II
Temperature (K)	100
Detector	Dectris EIGER2 XE 9M
Space group	*P*6_3_
*a*, *b*, *c* (Å)	75.46, 75.46, 36.95
α, β, γ (°)	90, 90, 120
Resolution range (Å)	37.73–1.80 (1.84–1.80)
Total No. of reflections	154623 (9558)
Completeness (%)	100.0 (100.0)
Multiplicity	13.7 (14.1)
〈*I*/σ(*I*)〉	11.2 (1.7)
*R*_r.i.m._	0.124 (1.948)
*R*_p.i.m._	0.034 (0.514)
CC_1/2_	0.994 (0.664)

**Table 4:** Structure refinement Value in parentheses are for the outer shell.

Resolution range (Å)	37.73–1.80 (1.84–1.80)
No. of reflections, working set	11293 (2664)
No. of reflections, test set	534 (123)
Final *R*_cryst_	0.187 (0.283)
Final *R*_free_	0.218 (0.287)
No. of non-H atoms	
Protein	734
Ligand	13
Water	19
Total	766
R.m.s. deviations	
Bond lengths (Å)	0.009
Angles (°)	0.944
Average *B* factors (Å^2^)	
Protein	54.9
Ligand	78.4
Water	50.3
*MolProbity* statistics	
Protein geometry	
Poor rotamers (%)	0
Favored rotamers (%)	91
Ramachandran outliers (%)	0
Ramachandran favored (%)	100
Ramachandran distribution *Z*-score	1.96 ± 0.82
C^β^ deviations > 0.25 Å (%)	0
Bad bonds (%)	0
Bad angles (%)	0
Peptide omegas *cis*-prolines (%)	0
Additional validations	0/117
