# Supplementary material for: Crystal structure of Plasmodium vivax macrophage migration inhibitory factor
Source: Acta Crystallogr F Struct Biol Commun. 2026 May 5;82(Pt 6):194–200. doi: 10.1107/S2053230X26003870 (PMC13224792; doi:10.1107/S2053230X26003870)
Supplement: Supplementary file 1 [file f-82-00194-sup1.pdf]

STRUCTURAL BIOLOGY
COMMUNICATIONS

Volume 82 (2026)

Supporting information for article:

Crystal structure of *Plasmodium vivax* macrophage migration inhibitory factor

Aryana Nair, Matthew Lin, Arav Srivastava, Lijun Liu, Anne Cooper, Kevin Battaile, Elizabeth Harmon, Peter J. Myler, Bart Staker, Scott Lovell, Graham Chakafana and Oluwatoyin A. Asojo

PDBe Fold v2.59. (src3) 14 Apr 2014 result file.

RESULT SUMMARY

##	Q-score	P-score	Z-score	RMSD	Nalgn	Nsse	Ngaps	Seq-%	Nmd	Nres-Q	Nsse-Q	Nres-T	Nsse-T	Query	Target
1	0.8834	15.82	11.79	0.695	95	7	2	0.6632	0	96	7	101	7	PDB 9b0m:A	PDB 2wkf:A
2	0.8443	13.04	10.67	0.676	89	6	3	0.6517	0	96	7	93	6	PDB 9b0m:A	PDB 4p7m:C
3	0.839	14.04	11.09	0.719	89	6	3	0.6517	0	96	7	93	6	PDB 9b0m:A	PDB 4p7s:C
4	0.8348	14.36	11.29	0.736	94	6	1	0.6809	0	96	7	104	7	PDB 9b0m:A	PDB 4p7m:A
5	0.834	14.31	11.27	0.742	94	6	1	0.6809	0	96	7	104	7	PDB 9b0m:A	PDB 4p7s:A
6	0.8205	13.78	11.05	0.680	91	6	2	0.6813	0	96	7	100	7	PDB 9b0m:A	PDB 4p7s:B
7	0.8197	13.74	11.03	0.686	91	6	2	0.6813	0	96	7	100	7	PDB 9b0m:A	PDB 4p7m:B
8	0.8098	9.951	9.414	1.070	95	6	2	0.2632	0	96	7	103	7	PDB 9b0m:A	PDB 8dq6:A
9	0.7842	13.7	11.02	0.983	94	6	1	0.6383	0	96	7	106	7	PDB 9b0m:A	PDB 2wkb:A
10	0.7712	11.62	10.18	0.735	95	6	2	0.6632	0	96	7	115	8	PDB 9b0m:A	PDB 3gac:D
11	0.7703	9.11	9.004	1.361	93	6	1	0.2366	0	96	7	97	7	PDB 9b0m:A	PDB 8dq6:C
12	0.77	11.81	10.21	0.745	95	6	2	0.6632	0	96	7	115	7	PDB 9b0m:A	PDB 3gad:D
13	0.7696	11.71	10.17	0.748	95	6	2	0.6632	0	96	7	115	7	PDB 9b0m:A	PDB 3gac:A
14	0.7694	11.81	10.21	0.750	95	6	2	0.6632	0	96	7	115	8	PDB 9b0m:A	PDB 3gad:A
15	0.7684	11.78	10.2	0.758	95	6	2	0.6632	0	96	7	115	7	PDB 9b0m:A	PDB 3gad:B
16	0.7651	11.84	10.23	0.730	95	6	2	0.6632	0	96	7	116	7	PDB 9b0m:A	PDB 3gac:F
17	0.7646	11.68	10.15	0.789	95	6	2	0.6632	0	96	7	115	7	PDB 9b0m:A	PDB 3gac:E
18	0.7605	11.71	10.17	0.769	95	6	2	0.6632	0	96	7	116	7	PDB 9b0m:A	PDB 3gac:B
19	0.7598	11.81	10.21	0.720	95	6	2	0.6632	0	96	7	117	7	PDB 9b0m:A	PDB 3gad:C
20	0.759	11.81	10.21	0.727	95	6	2	0.6632	0	96	7	117	7	PDB 9b0m:A	PDB 3gac:C
21	0.7577	11.81	10.21	0.738	95	6	2	0.6632	0	96	7	117	7	PDB 9b0m:A	PDB 3gad:F
22	0.7543	11.71	10.17	0.766	95	6	2	0.6632	0	96	7	117	8	PDB 9b0m:A	PDB 3gad:E
23	0.7497	12.72	10.61	0.977	94	6	2	0.617	0	96	7	111	7	PDB 9b0m:A	PDB 2wkb:D
24	0.7477	8.813	8.977	1.176	95	6	2	0.2632	0	96	7	109	8	PDB 9b0m:A	PDB 8dq6:B
25	0.7433	11.69	10.08	1.416	92	7	2	0.3587	0	96	7	97	7	PDB 9b0m:A	PDB 3t5s:A
26	0.7359	13.59	10.9	0.893	89	6	2	0.6517	0	96	7	103	6	PDB 9b0m:A	PDB 2wkf:B
27	0.7241	13.25	10.83	1.043	93	6	1	0.6344	0	96	7	111	7	PDB 9b0m:A	PDB 2wkb:B
28	0.7196	13.37	10.88	1.005	94	6	2	0.617	0	96	7	115	7	PDB 9b0m:A	PDB 2wkb:F
29	0.7147	12.55	10.54	1.215	95	7	3	0.2105	0	96	7	113	8	PDB 9b0m:A	PDB 3fwu:A
30	0.711	12.76	10.55	1.184	94	7	3	0.2021	0	96	7	112	7	PDB 9b0m:A	PDB 3b64:A
31	0.7037	13.41	10.9	0.966	93	6	1	0.6344	0	96	7	116	7	PDB 9b0m:A	PDB 2wkb:E
32	0.7025	10.3	9.693	1.251	95	6	1	0.2105	0	96	7	114	8	PDB 9b0m:A	PDB 6cuq:A
33	0.7006	13.25	10.83	1.132	94	6	2	0.617	0	96	7	115	7	PDB 9b0m:A	PDB 2wkb:C
34	0.6924	10.36	9.722	1.275	95	6	1	0.2105	0	96	7	115	8	PDB 9b0m:A	PDB 6cuq:C
35	0.6854	11.59	10.04	1.174	93	7	3	0.3226	0	96	7	114	8	PDB 9b0m:A	PDB 4dh4:A
36	0.6816	10.33	9.707	1.269	95	6	1	0.2105	0	96	7	117	8	PDB 9b0m:A	PDB 6cuq:B
37	0.6662	8.313	8.441	1.240	94	6	3	0.1809	0	96	7	118	7	PDB 9b0m:A	PDB 6vvw:F
38	0.6575	10.43	9.503	1.432	94	7	2	0.2447	0	96	7	114	7	PDB 9b0m:A	PDB 2xcz:A
39	0.6536	7.243	7.858	1.303	95	6	4	0.1895	0	96	7	121	8	PDB 9b0m:A	PDB 6vvw:E
40	0.6469	7.296	8.056	1.480	93	6	5	0.1183	0	96	7	112	7	PDB 9b0m:A	PDB 5uif:B
41	0.6418	7.417	8.122	1.589	94	6	6	0.117	0	96	7	112	7	PDB 9b0m:A	PDB 5uif:A
42	0.6389	7.385	7.938	1.362	95	6	4	0.1895	0	96	7	122	7	PDB 9b0m:A	PDB 6vvw:D
43	0.6341	6.85	7.635	1.267	92	6	4	0.1848	0	96	7	118	7	PDB 9b0m:A	PDB 6vvw:B
44	0.6311	6.962	7.872	1.326	94	6	4	0.1809	0	96	7	122	8	PDB 9b0m:A	PDB 5unq:D
45	0.6307	8.567	8.574	1.329	94	6	5	0.1915	0	96	7	122	8	PDB 9b0m:A	PDB 6vvw:H
46	0.6264	7.343	8.083	1.520	92	6	4	0.1196	0	96	7	112	7	PDB 9b0m:A	PDB 5uif:C
47	0.6255	6.962	7.872	1.329	94	6	4	0.1809	0	96	7	123	8	PDB 9b0m:A	PDB 5unq:A
48	0.6236	6.891	7.832	1.341	94	6	4	0.1809	0	96	7	123	8	PDB 9b0m:A	PDB 5unq:F
49	0.6231	7.368	8.096	1.325	93	5	6	0.1505	0	96	7	121	7	PDB 9b0m:A	PDB 7ms9:J
50	0.6207	6.821	7.793	1.360	94	6	4	0.1809	0	96	7	123	7	PDB 9b0m:A	PDB 5unq:E
51	0.614	9.976	9.285	1.309	91	7	5	0.1429	0	96	7	118	8	PDB 9b0m:A	PDB 3n4h:A
52	0.6104	9.039	8.823	1.453	92	7	8	0.1522	0	96	7	117	8	PDB 9b0m:A	PDB 2flt:A
53	0.6067	7.392	8.109	1.430	93	6	6	0.1505	0	96	7	121	7	PDB 9b0m:A	PDB 7ms9:A
54	0.6039	7.368	8.096	1.448	93	6	6	0.1505	0	96	7	121	7	PDB 9b0m:A	PDB 7ms9:E
55	0.6038	6.891	7.832	1.331	92	6	3	0.1848	0	96	7	122	8	PDB 9b0m:A	PDB 5unq:C
56	0.6022	7.465	8.149	1.286	91	6	5	0.1538	0	96	7	121	7	PDB 9b0m:A	PDB 7ms9:F
57	0.6016	7.489	8.162	1.290	91	6	5	0.1538	0	96	7	121	7	PDB 9b0m:A	PDB 7ms9:C
58	0.5991	7.587	8.215	1.308	91	6	5	0.1538	0	96	7	121	7	PDB 9b0m:A	PDB 7ms9:I
59	0.5986	7.417	8.122	1.311	91	6	5	0.1538	0	96	7	121	7	PDB 9b0m:A	PDB 7ms9:H
60	0.598	7.296	8.056	1.315	91	6	5	0.1538	0	96	7	121	7	PDB 9b0m:A	PDB 7ms9:K
61	0.5977	7.514	8.175	1.317	91	6	5	0.1538	0	96	7	121	7	PDB 9b0m:A	PDB 7ms9:L
62	0.5976	7.075	8.07	1.318	91	6	5	0.1538	0	96	7	121	8	PDB 9b0m:A	PDB 7ms9:G
63	0.5962	7.245	8.162	1.327	91	6	5	0.1538	0	96	7	121	8	PDB 9b0m:A	PDB 7ms9:D
64	0.5957	7.176	7.99	1.331	91	6	5	0.1538	0	96	7	121	7	PDB 9b0m:A	PDB 7ms9:B
65	0.5899	9.068	8.837	1.415	90	7	7	0.1556	0	96	7	117	8	PDB 9b0m:A	PDB 3mf8:A
66	0.5871	6.653	7.742	1.629	90	5	3	0.3	0	96	7	111	7	PDB 9b0m:A	PDB 4gum:E
67	0.5729	7.344	8.083	1.504	92	6	4	0.1522	0	96	7	123	8	PDB 9b0m:A	PDB 6vvr:A
68	0.5626	7.489	8.162	1.490	91	6	4	0.1538	0	96	7	123	8	PDB 9b0m:A	PDB 6vvr:C
69	0.5615	7.267	7.872	1.486	89	6	9	0.1348	0	96	7	118	7	PDB 9b0m:A	PDB 3mf7:A
70	0.5443	5.843	7.175	1.680	90	7	5	0.06667	0	96	7	118	9	PDB 9b0m:A	PDB 7tvk:F
71	0.5402	5.556	7.003	1.707	90	7	5	0.06667	0	96	7	118	9	PDB 9b0m:A	PDB 7tvk:C
72	0.5377	9.036	8.815	1.828	91	6	2	0.2198	0	96	7	117	8	PDB 9b0m:A	PDB 7mse:A
73	0.5337	8.878	8.734	1.853	91	6	2	0.2198	0	96	7	117	8	PDB 9b0m:A	PDB 8dbb:B
74	0.5236	8.013	8.281	1.767	89	6	2	0.2135	0	96	7	117	8	PDB 9b0m:A	PDB 3kan:C
75	0.5185	9.554	9.082	1.392	93	7	6	0.1505	0	96	7	143	10	PDB 9b0m:A	PDB 7ms1:B
76	0.517	5.82	7.161	1.703	88	7	6	0.06818	0	96	7	118	9	PDB 9b0m:A	PDB 7tvk:D
77	0.5155	8.063	8.308	1.820	89	6	2	0.2135	0	96	7	117	7	PDB 9b0m:A	PDB 1dpt:A
78	0.5139	5.82	7.161	1.695	88	7	6	0.06818	0	96	7	119	9	PDB 9b0m:A	PDB 7tvk:B
79	0.5133	5.792	7.147	1.728	88	7	6	0.06818	0	96	7	118	9	PDB 9b0m:A	PDB 7tvk:A
80	0.5086	8.92	8.765	1.439	93	7	6	0.1505	0	96	7	144	9	PDB 9b0m:A	PDB 7ms3:A
81	0.5075	9.26	8.938	1.474	93	7	6	0.1505	0	96	7	143	9	PDB 9b0m:A	PDB 7ms1:A
82	0.5056	4.187	6.089	1.993	92	6	6	0.08696	0	96	7	121	7	PDB 9b0m:A	PDB 4lkb:F
83	0.498	9.439	9.024	1.467	93	7	6	0.1505	0	96	7	146	9	PDB 9b0m:A	PDB 7ms8:A
84	0.4947	4.664	6.408	1.845	89	6	6	0.08989	0	96	7	121	7	PDB 9b0m:A	PDB 4lkb:A
85	0.4889	9.174	8.895	1.511	93	7	7	0.1505	0	96	7	147	9	PDB 9b0m:A	PDB 2flz:A
86	0.4827	4.506	6.302	2.317	95	6	4	0.08421	0	96	7	122	7	PDB 9b0m:A	PDB 4lkb:B
87	0.4815	5.227	6.845	1.844	91	6	4	0.1648	0	96	7	130	8	PDB 9b0m:A	PDB 4lhp:K
88	0.479	6.158	7.411	1.960	92	6	4	0.1413	0	96	7	129	8	PDB 9b0m:A	PDB 2aal:E
89	0.4784	5.187	6.818	1.892	91	6	4	0.1648	0	96	7	129	7	PDB 9b0m:A	PDB 3mlc:B
90	0.4783	5.478	6.805	1.818	90	6	5	0.1667	0	96	7	129	8	PDB 9b0m:A	PDB 3mlc:E
91	0.478	6.114	7.385	1.967	92	6	4	0.1413	0	96	7	129	7	PDB 9b0m:A	PDB 2aal:C

92	0.4773	4.468	6.275	1.890	88	6	7	0.07955	0	96	7	121	7	PDB 9b0m:A	PDB 4lkb:D
93	0.477	5.207	6.831	1.850	91	6	4	0.1648	0	96	7	131	8	PDB 9b0m:A	PDB 3mjz:H
94	0.4768	6.092	7.371	1.975	92	6	4	0.1413	0	96	7	129	8	PDB 9b0m:A	PDB 2aal:D
95	0.4765	6.16	7.411	1.977	92	6	4	0.1413	0	96	7	129	8	PDB 9b0m:A	PDB 2aal:F
96	0.4746	5.289	6.884	1.793	90	6	5	0.1667	0	96	7	131	8	PDB 9b0m:A	PDB 4lho:C
97	0.4744	5.207	6.831	1.869	91	6	4	0.1648	0	96	7	131	8	PDB 9b0m:A	PDB 3mjz:A
98	0.4734	5.207	6.831	1.876	91	6	4	0.1648	0	96	7	131	7	PDB 9b0m:A	PDB 3mjz:J
99	0.4733	5.187	6.818	1.877	91	6	4	0.1648	0	96	7	131	8	PDB 9b0m:A	PDB 3mjz:D
100	0.473	5.166	6.805	1.879	91	6	4	0.1648	0	96	7	131	8	PDB 9b0m:A	PDB 4lhp:H
101	0.4727	5.187	6.818	1.881	91	6	4	0.1648	0	96	7	131	8	PDB 9b0m:A	PDB 4lhp:J
102	0.4723	5.146	6.792	1.884	91	6	4	0.1648	0	96	7	131	8	PDB 9b0m:A	PDB 4lhp:A
103	0.4722	8.64	8.621	1.529	91	7	8	0.1538	0	96	7	145	9	PDB 9b0m:A	PDB 2flz:C
104	0.472	5.207	6.831	1.885	91	6	4	0.1648	0	96	7	131	7	PDB 9b0m:A	PDB 3mjz:L
105	0.4718	5.227	6.845	1.887	91	6	4	0.1648	0	96	7	131	8	PDB 9b0m:A	PDB 4lhp:D
106	0.4715	6.003	7.319	2.011	92	6	4	0.1413	0	96	7	129	8	PDB 9b0m:A	PDB 2aal:A
107	0.4715	5.227	6.845	1.889	91	6	4	0.1648	0	96	7	131	8	PDB 9b0m:A	PDB 4lho:A
108	0.4711	5.609	7.082	1.690	88	6	4	0.1818	0	96	7	130	7	PDB 9b0m:A	PDB 4lhp:F
109	0.471	6.005	7.319	2.015	92	6	4	0.1413	0	96	7	129	8	PDB 9b0m:A	PDB 2aag:F
110	0.4707	5.204	6.831	1.895	91	6	4	0.1648	0	96	7	131	7	PDB 9b0m:A	PDB 4lhp:L
111	0.4689	5.611	7.082	1.706	88	6	4	0.1818	0	96	7	130	7	PDB 9b0m:A	PDB 3mjz:F
112	0.4689	6.07	7.358	1.784	89	6	3	0.1798	0	96	7	130	8	PDB 9b0m:A	PDB 3mjz:K
113	0.4689	6.003	7.319	2.029	92	6	4	0.1413	0	96	7	129	8	PDB 9b0m:A	PDB 2aag:D
114	0.4681	6.025	7.332	2.035	92	6	4	0.1413	0	96	7	129	8	PDB 9b0m:A	PDB 2aag:E
115	0.4681	5.23	6.845	1.816	89	6	3	0.1685	0	96	7	129	8	PDB 9b0m:A	PDB 3mlc:A
116	0.4673	6.003	7.319	2.016	92	6	4	0.1413	0	96	7	130	8	PDB 9b0m:A	PDB 2aal:B
117	0.4672	8.892	8.751	1.487	90	7	8	0.1556	0	96	7	145	9	PDB 9b0m:A	PDB 2flz:B
118	0.4672	5.893	7.253	1.770	89	6	3	0.1685	0	96	7	131	8	PDB 9b0m:A	PDB 3mjz:G
119	0.4672	6.97	7.72	1.920	86	7	6	0.1744	0	96	7	117	8	PDB 9b0m:A	PDB 8dbb:C
120	0.4666	5.981	7.306	2.045	92	6	4	0.1413	0	96	7	129	8	PDB 9b0m:A	PDB 2aag:A
121	0.4666	5.504	7.016	1.775	89	6	3	0.1798	0	96	7	131	8	PDB 9b0m:A	PDB 4lhp:G
122	0.4663	5.958	7.293	2.047	92	6	4	0.1413	0	96	7	129	8	PDB 9b0m:A	PDB 2aag:C
123	0.4647	4.173	6.076	1.978	88	6	5	0.09091	0	96	7	121	7	PDB 9b0m:A	PDB 4lkb:C
124	0.4643	5.567	7.056	1.791	89	6	3	0.1798	0	96	7	131	7	PDB 9b0m:A	PDB 3mjz:C
125	0.4639	5.462	6.99	1.716	88	6	4	0.1818	0	96	7	131	8	PDB 9b0m:A	PDB 4lhp:C
126	0.4636	5.333	6.911	1.693	87	6	4	0.1839	0	96	7	129	8	PDB 9b0m:A	PDB 3mlc:C
127	0.4634	5.481	7.003	1.798	89	6	3	0.1685	0	96	7	131	7	PDB 9b0m:A	PDB 4lho:B
128	0.4634	5.936	7.279	2.043	92	6	4	0.1413	0	96	7	130	8	PDB 9b0m:A	PDB 2aag:B
129	0.4582	5.418	6.963	1.680	87	6	5	0.1724	0	96	7	131	8	PDB 9b0m:A	PDB 4lhp:B
130	0.457	5.483	7.003	1.689	87	6	5	0.1724	0	96	7	131	8	PDB 9b0m:A	PDB 4lhp:I
131	0.4569	5.548	7.042	1.769	88	6	3	0.1818	0	96	7	131	8	PDB 9b0m:A	PDB 3mjz:E
132	0.4569	5.504	7.016	1.690	87	6	4	0.1839	0	96	7	131	8	PDB 9b0m:A	PDB 4lhp:E
133	0.4563	5.395	6.95	1.695	87	6	5	0.1724	0	96	7	131	8	PDB 9b0m:A	PDB 3mjz:B
134	0.4558	5.677	7.121	2.048	84	6	6	0.25	0	96	7	110	7	PDB 9b0m:A	PDB 4gum:G
135	0.4558	5.46	6.99	1.698	87	6	5	0.1724	0	96	7	131	7	PDB 9b0m:A	PDB 3mjz:I
136	0.4551	4.039	5.982	2.418	93	6	5	0.08602	0	96	7	120	7	PDB 9b0m:A	PDB 4lkb:E
137	0.4482	6.378	7.491	2.055	86	7	6	0.1512	0	96	7	117	8	PDB 9b0m:A	PDB 4nwp:H
138	0.4466	5.868	7.405	2.039	86	7	6	0.1512	0	96	7	118	9	PDB 9b0m:A	PDB 4nwp:G
139	0.4454	3.961	5.928	2.212	89	5	6	0.1573	0	96	7	120	7	PDB 9b0m:A	PDB 1mww:A
140	0.445	6.322	7.591	2.051	86	7	6	0.1512	0	96	7	118	9	PDB 9b0m:A	PDB 4nwp:E
141	0.439	3.305	5.538	2.369	91	5	5	0.0989	0	96	7	121	7	PDB 9b0m:A	PDB 7yxv:A
142	0.4297	3.4	5.423	2.511	85	7	7	0.1647	0	96	7	103	8	PDB 9b0m:A	PDB 1fim:A
143	0.4251	4.563	6.235	2.704	86	7	6	0.1744	0	96	7	100	7	PDB 9b0m:A	PDB 8dqa:A
144	0.4203	6.302	7.577	2.078	85	7	6	0.1412	0	96	7	121	9	PDB 9b0m:A	PDB 4nwq:B
145	0.4193	2.423	5.048	2.573	84	6	6	0.1429	0	96	7	101	8	PDB 9b0m:A	PDB 4gum:H
146	0.3954	2.581	5.172	2.690	86	6	5	0.1279	0	96	7	108	8	PDB 9b0m:A	PDB 4gum:F
147	0.3945	6.116	7.219	2.178	84	7	7	0.1429	0	96	7	122	7	PDB 9b0m:A	PDB 1u9d:B
148	0.3942	6.089	7.204	2.181	84	7	7	0.1429	0	96	7	122	7	PDB 9b0m:A	PDB 1u9d:A
149	0.3916	2.986	5.108	2.692	86	7	7	0.0814	0	96	7	109	8	PDB 9b0m:A	PDB 7mrv:A
150	0.3902	3.482	5.483	2.637	87	7	6	0.1609	0	96	7	114	8	PDB 9b0m:A	PDB 5bsi:G
151	0.3885	3.298	5.349	2.553	86	7	6	0.186	0	96	7	115	8	PDB 9b0m:A	PDB 1uiz:D
152	0.3824	4.137	6.351	2.207	89	7	6	0.1348	0	96	7	140	10	PDB 9b0m:A	PDB 4u5p:A
153	0.3816	3.475	5.483	2.634	86	7	6	0.1628	0	96	7	114	8	PDB 9b0m:A	PDB 5bsi:J
154	0.3812	2.477	5.184	2.637	86	7	6	0.1628	0	96	7	114	10	PDB 9b0m:A	PDB 4osf:C
155	0.3802	2.661	5.319	2.645	86	7	6	0.1395	0	96	7	114	10	PDB 9b0m:A	PDB 2ooh:B
156	0.3788	2.537	5.144	2.693	85	6	8	0.1412	0	96	7	110	8	PDB 9b0m:A	PDB 4gum:D
157	0.3786	2.541	5.229	2.658	86	7	6	0.1628	0	96	7	114	10	PDB 9b0m:A	PDB 7xvx:B
158	0.3786	2.606	5.274	2.515	84	7	7	0.1667	0	96	7	114	10	PDB 9b0m:A	PDB 5hvs:B
159	0.3776	2.559	5.158	2.763	87	6	6	0.1264	0	96	7	113	8	PDB 9b0m:A	PDB 4gum:I
160	0.3768	3.153	5.172	2.672	86	6	6	0.1047	0	96	7	114	6	PDB 9b0m:A	PDB 1mif:C
161	0.3766	2.925	5.513	2.674	86	7	6	0.1744	0	96	7	114	10	PDB 9b0m:A	PDB 1mif:B
162	0.3759	3.542	5.619	2.601	86	6	7	0.1395	0	96	7	117	7	PDB 9b0m:A	PDB 4gro:A
163	0.3753	2.56	5.244	2.614	85	7	6	0.1647	0	96	7	114	10	PDB 9b0m:A	PDB 4k9g:B
164	0.3735	3.688	5.727	2.691	87	6	7	0.1264	0	96	7	117	7	PDB 9b0m:A	PDB 4gro:G
165	0.3715	3.424	5.808	2.500	83	6	6	0.1205	0	96	7	114	8	PDB 9b0m:A	PDB 8ap3:A
166	0.3683	3.497	5.861	2.573	84	6	6	0.131	0	96	7	115	8	PDB 9b0m:A	PDB 8ap3:B
167	0.3671	2.722	5.169	2.889	88	7	5	0.1591	0	96	7	114	9	PDB 9b0m:A	PDB 4gum:A
168	0.3647	3.943	5.767	1.676	73	6	6	0.09589	0	96	7	116	6	PDB 9b0m:A	PDB 3fwt:A
169	0.3638	2.246	4.925	2.874	87	6	4	0.1034	0	96	7	113	8	PDB 9b0m:A	PDB 1hfo:F
170	0.3635	4.364	6.047	2.417	67	5	6	0.08955	0	96	7	78	6	PDB 9b0m:A	PDB 7ywa:A
171	0.363	2.614	5.199	2.497	82	6	7	0.1463	0	96	7	114	8	PDB 9b0m:A	PDB 4wr8:R
172	0.3502	1.692	3.587	3.069	80	5	5	0.075	0	96	7	93	6	PDB 9b0m:A	PDB 1qjh:A
173	0.3493	4.161	5.903	2.331	66	5	6	0.07576	0	96	7	81	6	PDB 9b0m:A	PDB 1ghh:A
174	0.3462	4.429	6.249	2.640	82	6	5	0.2805	0	96	7	114	8	PDB 9b0m:A	PDB 1mif:A
175	0.3461	1.592	3.486	3.096	82	5	5	0.08537	0	96	7	98	6	PDB 9b0m:A	PDB 2bxj:B
176	0.338	2.243	4.515	2.921	86	7	6	0.09302	0	96	7	117	8	PDB 9b0m:A	PDB 4q3f:A
177	0.3375	1.956	4.485	2.995	87	7	6	0.08046	0	96	7	117	10	PDB 9b0m:A	PDB 7mse:C
178	0.337	2.378	4.623	2.930	86	7	7	0.0814	0	96	7	117	9	PDB 9b0m:A	PDB 3kan:B
179	0.3353	0.8764	3.73	3.061	79	5	6	0.1139	0	96	7	95	6	PDB 9b0m:A	PDB 8e6y:A
180	0.3338	3.33	5.741	2.161	78	6	6	0.1154	0	96	7	125	8	PDB 9b0m:A	PDB 1otg:B
181	0.332	2.287	4.467	2.733	84	6	8	0.09524	0	96	7	121	7	PDB 9b0m:A	PDB 6vvw:A
182	0.3318	2.244	4.515	2.977	86	7	7	0.0814	0	96	7	117	8	PDB 9b0m:A	PDB 4q3f:C
183	0.3306	1.584	3.471	3.253	81	5	4	0.09877	0	96	7	95	6	PDB 9b0m:A	PDB 6i6e:A
184	0.3288	2.466	4.699	3.004	86	7	5	0.06977	0	96	7	117	8	PDB 9b0m:A	PDB 3ker:D
185	0.3288	1.862	4.699	3.004	86	7	5	0.06977	0	96	7	117	10	PDB 9b0m:A	PDB 3ker:C
186	0.3286	1.989	4.201	3.135	80	6	6	0.1125	0	96	7	97	6	PDB 9b0m:A	PDB 1fka:F
187	0.3275	1.571	3.471	3.250	81	5	4	0.09877	0	96	7	96	6	PDB 9b0m:A	PDB 6i69:A
188	0.323	1.694	3.931	2.670	82	5	7	0.08537	0	96	7	121	6	PDB 9b0m:A	PDB 6vvw:C

189	0.3123	0.7784	2.989	2.748	66	6	5	0.1061	0	96	7	79	6	PDB 9b0m:A	PDB 4rjv:C
190	0.3123	3.791	5.408	2.735	77	6	6	0.07792	0	96	7	108	8	PDB 9b0m:A	PDB 4m1a:A
191	0.3121	3.809	5.423	2.737	77	6	6	0.07792	0	96	7	108	8	PDB 9b0m:A	PDB 4m1a:B
192	0.3112	3.029	5.076	2.931	83	6	5	0.09639	0	96	7	118	8	PDB 9b0m:A	PDB 2os5:A
193	0.311	1.239	3.434	2.756	68	5	5	0.05882	0	96	7	84	5	PDB 9b0m:A	PDB 6qfj:A
194	0.3106	1.382	3.268	3.183	80	5	5	0.1	0	96	7	101	6	PDB 9b0m:A	PDB 4dv2:F
195	0.31	1.312	3.512	2.712	67	5	5	0.0597	0	96	7	83	5	PDB 9b0m:A	PDB 6qfj:B
196	0.3081	0.9852	3.224	3.003	73	5	5	0.06849	0	96	7	90	6	PDB 9b0m:A	PDB 6wi5:B
197	0.3081	0.7651	2.975	2.836	71	6	5	0.07042	0	96	7	90	6	PDB 9b0m:A	PDB 6wi5:A
198	0.3057	1.364	3.253	2.922	76	5	6	0.07895	0	96	7	101	6	PDB 9b0m:A	PDB 5lmq:F
199	0.3023	1.296	3.18	3.188	79	5	6	0.08861	0	96	7	101	6	PDB 9b0m:A	PDB 5lmo:F
200	0.3006	1.471	3.37	3.129	78	5	6	0.1026	0	96	7	101	6	PDB 9b0m:A	PDB 5lmt:F
201	0.3005	1.448	3.931	3.153	85	6	6	0.05882	0	96	7	119	8	PDB 9b0m:A	PDB 7tvk:E
202	0.3004	0.6051	3.395	2.895	75	5	8	0.09333	0	96	7	101	6	PDB 9b0m:A	PDB 5lmn:F
203	0.2996	0.6541	2.842	3.215	79	5	5	0.08861	0	96	7	101	6	PDB 9b0m:A	PDB 1ibl:F
204	0.2995	1.065	3.252	2.730	58	5	5	0.1207	0	96	7	64	5	PDB 9b0m:A	PDB 6wmk:D
205	0.299	0.7365	3.576	2.656	64	5	5	0.09375	0	96	7	80	6	PDB 9b0m:A	PDB 4rjv:B
206	0.2976	1.138	3.384	3.159	78	6	5	0.08974	0	96	7	101	6	PDB 9b0m:A	PDB 4dv0:F
207	0.2975	0.5821	2.686	3.135	70	5	6	0.01429	0	96	7	82	5	PDB 9b0m:A	PDB 3byp:B
208	0.2971	0.8405	3.692	3.007	76	5	7	0.09211	0	96	7	101	6	PDB 9b0m:A	PDB 1n34:F
209	0.2962	0.8701	3.092	3.026	69	6	5	0.1014	0	96	7	83	6	PDB 9b0m:A	PDB 4rjv:A
210	0.2961	0.9008	3.069	2.668	65	5	5	0.06154	0	96	7	83	5	PDB 9b0m:A	PDB 6qfj:C
211	0.2959	0.7612	2.911	2.836	66	5	6	0.06061	0	96	7	81	5	PDB 9b0m:A	PDB 6qfj:D
212	0.2951	0.7667	2.975	3.184	78	5	6	0.1154	0	96	7	101	6	PDB 9b0m:A	PDB 2uxb:F
213	0.2937	1.414	3.615	2.766	65	5	5	0.06154	0	96	7	81	5	PDB 9b0m:A	PDB 6qfj:E
214	0.2934	1.326	3.209	3.201	78	5	6	0.1154	0	96	7	101	6	PDB 9b0m:A	PDB 5lms:F
215	0.2929	1.001	3.871	2.796	68	5	5	0.1324	0	96	7	88	7	PDB 9b0m:A	PDB 2mtl:A
216	0.2923	1.097	3.341	2.864	71	6	4	0.05634	0	96	7	94	6	PDB 9b0m:A	PDB 2lqj:A
217	0.2914	0.8412	3.063	3.090	70	6	5	0.1571	0	96	7	85	6	PDB 9b0m:A	PDB 2kl8:A
218	0.2909	1.818	4.388	2.863	78	5	6	0.1154	0	96	7	114	7	PDB 9b0m:A	PDB 4gum:B
219	0.2895	1.426	3.628	2.797	68	5	4	0.07353	0	96	7	89	5	PDB 9b0m:A	PDB 4oyc:B
220	0.289	0.7758	2.989	2.648	64	5	5	0.09375	0	96	7	83	6	PDB 9b0m:A	PDB 4rjv:D
221	0.2876	0.1321	2.057	3.311	70	5	6	0.08571	0	96	7	80	5	PDB 9b0m:A	PDB 4y0l:A
222	0.2862	0.7368	2.486	3.433	73	5	6	0.09589	0	96	7	84	6	PDB 9b0m:A	PDB 7bfd:D
223	0.2858	0.8143	3.019	3.280	78	5	6	0.08974	0	96	7	101	6	PDB 9b0m:A	PDB 4ji8:F
224	0.2854	0.4913	3.282	3.248	82	6	4	0.08537	0	96	7	113	8	PDB 9b0m:A	PDB 5mmj:f
225	0.2842	1.184	3.063	2.816	72	5	7	0.09722	0	96	7	101	6	PDB 9b0m:A	PDB 5lmp:F
226	0.2823	0.9022	3.069	2.697	63	5	5	0.06349	0	96	7	81	5	PDB 9b0m:A	PDB 6qfj:F
227	0.2823	0.5871	2.686	3.207	69	5	3	0.07246	0	96	7	82	5	PDB 9b0m:A	PDB 3byp:A
228	0.2817	2.162	4.355	3.063	79	6	7	0.1013	0	96	7	113	8	PDB 9b0m:A	PDB 6c5f:B
229	0.2795	0.6885	2.426	3.504	73	5	6	0.09589	0	96	7	84	6	PDB 9b0m:A	PDB 7bfd:I
230	0.279	2.232	4.411	3.063	80	6	7	0.125	0	96	7	117	7	PDB 9b0m:A	PDB 8dbb:A
231	0.2787	0.7156	2.916	3.550	68	5	7	0.1176	0	96	7	72	6	PDB 9b0m:A	PDB 1fvs:A
232	0.2786	0.7156	2.916	3.280	77	6	5	0.09091	0	96	7	101	6	PDB 9b0m:A	PDB 4k0k:F
233	0.2785	1.283	3.107	3.226	70	5	6	0.07143	0	96	7	85	6	PDB 9b0m:A	PDB 7b8v:F
234	0.278	2.241	4.425	3.074	80	6	7	0.125	0	96	7	117	8	PDB 9b0m:A	PDB 6c5f:A
235	0.2774	2.212	4.397	3.080	80	6	7	0.125	0	96	7	117	8	PDB 9b0m:A	PDB 6c5f:C
236	0.2772	0.2976	2.336	3.527	78	5	8	0.08974	0	96	7	96	6	PDB 9b0m:A	PDB 5o5j:F
237	0.2768	1.61	3.807	2.799	65	5	5	0.04615	0	96	7	85	5	PDB 9b0m:A	PDB 6vd8:A
238	0.2761	0.6244	2.336	3.541	73	5	6	0.09589	0	96	7	84	6	PDB 9b0m:A	PDB 7bfd:F
239	0.276	1.154	3.399	3.575	67	5	7	0.1194	0	96	7	70	6	PDB 9b0m:A	PDB 5vde:D
240	0.276	2.162	4.355	3.096	80	6	7	0.125	0	96	7	117	7	PDB 9b0m:A	PDB 4q3f:B
241	0.2752	0.61	2.321	3.551	73	5	6	0.09589	0	96	7	84	6	PDB 9b0m:A	PDB 7bfd:B
242	0.2749	0.8261	2.977	3.183	67	5	4	0.04478	0	96	7	80	5	PDB 9b0m:A	PDB 3w5y:B
243	0.2748	0.6809	2.871	3.604	70	5	6	0.04286	0	96	7	76	6	PDB 9b0m:A	PDB 1s6u:A
244	0.2742	0.5881	2.292	3.563	73	5	6	0.09589	0	96	7	84	6	PDB 9b0m:A	PDB 7bfd:E
245	0.2729	1.572	3.817	3.103	74	5	4	0.1081	0	96	7	101	6	PDB 9b0m:A	PDB 2uxd:F
246	0.2725	0.2269	2.217	3.514	64	5	9	0.03125	0	96	7	66	6	PDB 9b0m:A	PDB 2roe:A
247	0.2722	1.111	3.355	3.574	67	5	7	0.1194	0	96	7	71	6	PDB 9b0m:A	PDB 5vde:C
248	0.2722	0.3953	2.419	3.763	82	5	6	0.09756	0	96	7	100	5	PDB 9b0m:A	PDB 2ykr:F
249	0.2721	0.5804	2.753	3.122	68	5	4	0.1176	0	96	7	85	7	PDB 9b0m:A	PDB 4pww:A
250	0.2718	0.2634	2.191	3.345	68	5	5	0.01471	0	96	7	79	5	PDB 9b0m:A	PDB 3w63:A
251	0.2716	0.781	2.53	3.592	73	5	5	0.09589	0	96	7	84	6	PDB 9b0m:A	PDB 7bfd:C
252	0.2716	1.125	3.37	3.581	67	5	7	0.1194	0	96	7	71	6	PDB 9b0m:A	PDB 5vde:A
253	0.2715	2.631	4.748	2.967	78	5	8	0.05128	0	96	7	118	7	PDB 9b0m:A	PDB 7mw7:A
254	0.2712	0.2433	2.165	3.239	68	5	5	0.01471	0	96	7	82	5	PDB 9b0m:A	PDB 6han:D
255	0.2707	0.2326	2.151	3.282	68	5	5	0.01471	0	96	7	81	5	PDB 9b0m:A	PDB 3w8g:A
256	0.2705	0.8835	3.107	3.359	68	5	6	0.04412	0	96	7	79	6	PDB 9b0m:A	PDB 3qsi:J
257	0.2701	0.587	2.292	3.537	73	5	6	0.09589	0	96	7	86	6	PDB 9b0m:A	PDB 7b90:L
258	0.2696	0.2643	2.205	3.331	68	5	5	0.01471	0	96	7	80	5	PDB 9b0m:A	PDB 3w60:A
259	0.2695	0.5123	2.187	3.615	73	5	5	0.09589	0	96	7	84	6	PDB 9b0m:A	PDB 7bfd:G
260	0.2693	0.2936	2.336	3.101	65	6	8	0.1077	0	96	7	79	6	PDB 9b0m:A	PDB 3ibw:A
261	0.2689	0.6467	2.366	3.538	72	5	6	0.09722	0	96	7	84	6	PDB 9b0m:A	PDB 7bfd:A
262	0.2689	2.228	4.411	3.098	79	6	8	0.1139	0	96	7	117	7	PDB 9b0m:A	PDB 1dpt:B
263	0.2688	0.756	2.96	3.378	64	5	9	0.09375	0	96	7	70	6	PDB 9b0m:A	PDB 3k7r:H
264	0.2687	0.3991	2.023	3.835	76	5	4	0.09211	0	96	7	85	6	PDB 9b0m:A	PDB 7b8v:M
265	0.2687	0.8872	3.056	3.141	67	5	5	0.1343	0	96	7	83	5	PDB 9b0m:A	PDB 2zzt:A
266	0.2687	0.5513	2.247	3.708	74	5	4	0.09459	0	96	7	84	6	PDB 9b0m:A	PDB 7bfd:J
267	0.2686	1.194	3.442	3.573	67	5	7	0.1194	0	96	7	72	6	PDB 9b0m:A	PDB 5vde:B
268	0.2683	0.5429	2.232	3.711	74	5	6	0.09459	0	96	7	84	6	PDB 9b0m:A	PDB 7bfd:H
269	0.2683	0.2151	2.111	3.346	68	5	5	0.01471	0	96	7	80	5	PDB 9b0m:A	PDB 5hsp:A
270	0.2681	0.2667	2.205	3.348	68	5	5	0.01471	0	96	7	80	5	PDB 9b0m:A	PDB 3w61:A
271	0.2678	0.2363	2.151	3.352	68	5	5	0.01471	0	96	7	80	5	PDB 9b0m:A	PDB 6hhs:G
272	0.2676	0.8958	3.122	3.584	67	6	7	0.1194	0	96	7	72	6	PDB 9b0m:A	PDB 1cc8:A
273	0.2673	0.7029	2.901	3.445	65	5	7	0.1077	0	96	7	71	6	PDB 9b0m:A	PDB 3k7r:I
274	0.2671	0.5828	2.686	3.127	69	5	6	0.01449	0	96	7	89	6	PDB 9b0m:A	PDB 3byr:A
275	0.2671	0.562	2.723	3.270	64	5	8	0.07812	0	96	7	73	6	PDB 9b0m:A	PDB 7qzd:A
276	0.2665	0.1353	2.023	3.692	69	5	5	0.08696	0	96	7	74	6	PDB 9b0m:A	PDB 4p7t:C
277	0.2665	0.3445	1.948	3.861	76	5	5	0.09211	0	96	7	85	6	PDB 9b0m:A	PDB 7b8v:L
278	0.2662	0.574	2.738	3.373	65	5	7	0.09231	0	96	7	73	6	PDB 9b0m:A	PDB 2ggp:A
279	0.2662	0.6415	2.827	3.344	73	5	7	0.08219	0	96	7	93	6	PDB 9b0m:A	PDB 7mn2:A
280	0.266	0.5132	2.664	3.449	68	5	7	0.07353	0	96	7	78	6	PDB 9b0m:A	PDB 8b2r:A
281	0.266	0.2667	2.205	3.372	68	5	5	0.01471	0	96	7	80	5	PDB 9b0m:A	PDB 3w5z:A
282	0.2655	0.4048	2.038	3.873	76	5	4	0.09211	0	96	7	85	6	PDB 9b0m:A	PDB 7b8v:T
283	0.2654	2.146	4.341	3.137	79	6	8	0.1139	0	96	7	117	7	PDB 9b0m:A	PDB 1dpt:C
284	0.2652	1.837	4.023	2.963	65	5	4	0.1077	0	96	7	84	6	PDB 9b0m:A	PDB 4pug:B
285	0.2651	0.2305	2.138	3.344	68	5	5	0.01471	0	96	7	81	5	PDB 9b0m:A	PDB 6gmv:A

286	0.2649	0.2419	2.165	3.436	69	5	4	0.01449	0	96	7	81	5	PDB 9b0m:A	PDB 6hhs:C
287	0.2647	0.6821	2.871	3.603	64	5	6	0.1406	0	96	7	66	6	PDB 9b0m:A	PDB 1fe0:A
288	0.2643	0.6191	2.798	3.621	67	6	9	0.08955	0	96	7	72	6	PDB 9b0m:A	PDB 7qpx:A
289	0.2641	1.137	3.384	3.624	67	6	7	0.1194	0	96	7	72	6	PDB 9b0m:A	PDB 5vdf:E
290	0.2639	0.2204	2.124	3.248	68	5	5	0.01471	0	96	7	84	5	PDB 9b0m:A	PDB 6han:B
291	0.2639	0.3575	1.963	3.891	76	5	5	0.09211	0	96	7	85	6	PDB 9b0m:A	PDB 7b8v:G
292	0.2632	0.2326	2.151	3.366	68	5	5	0.01471	0	96	7	81	5	PDB 9b0m:A	PDB 6h85:A
293	0.2631	0.6925	2.886	3.575	64	6	6	0.1406	0	96	7	67	6	PDB 9b0m:A	PDB 4qot:A
294	0.263	0.294	2.245	3.407	68	5	5	0.01471	0	96	7	80	5	PDB 9b0m:A	PDB 6gmt:A
295	0.263	0.3858	2.486	3.545	66	5	6	0.1061	0	96	7	72	6	PDB 9b0m:A	PDB 3k7r:G
296	0.262	0.5829	2.753	3.634	64	5	6	0.1406	0	96	7	66	6	PDB 9b0m:A	PDB 3iwl:A
297	0.2619	0.695	2.886	3.465	65	5	7	0.1077	0	96	7	72	6	PDB 9b0m:A	PDB 3k7r:E
298	0.2611	0.5111	2.262	3.584	78	5	6	0.08974	0	96	7	100	6	PDB 9b0m:A	PDB 5uz4:F
299	0.2611	0.5186	2.202	3.924	76	5	4	0.09211	0	96	7	85	6	PDB 9b0m:A	PDB 7bfc:F
300	0.2611	0.5773	2.277	3.592	72	5	5	0.08333	0	96	7	85	6	PDB 9b0m:A	PDB 7bff:F
301	0.261	0.5881	2.292	3.557	72	5	5	0.09722	0	96	7	86	6	PDB 9b0m:A	PDB 7b90:C
302	0.2609	0.05772	2.142	2.897	66	6	6	0.1212	0	96	7	90	7	PDB 9b0m:A	PDB 2bje:G
303	0.2608	0.4383	2.56	3.662	67	5	6	0.1045	0	96	7	72	6	PDB 9b0m:A	PDB 3k7r:C
304	0.2606	0.4818	2.62	3.573	66	5	6	0.1061	0	96	7	72	6	PDB 9b0m:A	PDB 5vdf:B
305	0.2605	0.7023	3.187	2.897	61	5	3	0.1475	0	96	7	77	6	PDB 9b0m:A	PDB 6mrs:A
306	0.2604	0.4731	2.605	3.482	65	5	8	0.09231	0	96	7	72	6	PDB 9b0m:A	PDB 3k7r:J
307	0.2602	0.5098	2.664	3.308	69	5	6	0.07246	0	96	7	86	6	PDB 9b0m:A	PDB 7b90:K
308	0.2602	1.032	3.213	3.303	59	5	6	0.1864	0	96	7	63	5	PDB 9b0m:A	PDB 4a46:C
309	0.2601	0.2825	2.232	3.440	68	5	4	0.01471	0	96	7	80	5	PDB 9b0m:A	PDB 6h9q:A
310	0.26	0.2326	2.151	3.455	69	5	4	0.01449	0	96	7	82	5	PDB 9b0m:A	PDB 6hhs:E
311	0.26	1.007	3.239	3.394	64	5	8	0.1094	0	96	7	72	6	PDB 9b0m:A	PDB 5vdf:H
312	0.2597	0.53	2.217	3.656	73	5	5	0.09589	0	96	7	86	6	PDB 9b0m:A	PDB 7bfg:E
313	0.2596	1.329	3.525	2.979	66	5	4	0.0303	0	96	7	88	5	PDB 9b0m:A	PDB 6sd2:c
314	0.2595	0.9193	3.151	3.229	68	6	5	0.07353	0	96	7	86	6	PDB 9b0m:A	PDB 7bfg:L
315	0.2594	0.4348	2.082	3.826	75	5	5	0.09333	0	96	7	86	6	PDB 9b0m:A	PDB 7b90:A
316	0.2594	0.3034	2.351	3.630	66	5	6	0.0303	0	96	7	71	6	PDB 9b0m:A	PDB 2rsq:A
317	0.2594	0.1651	2.003	3.411	68	5	5	0.01471	0	96	7	81	5	PDB 9b0m:A	PDB 6h9p:C
318	0.2592	0.5186	2.202	3.662	73	5	5	0.09589	0	96	7	86	6	PDB 9b0m:A	PDB 7b90:E
319	0.2592	0.1767	2.726	3.428	62	5	6	0.129	0	96	7	67	6	PDB 9b0m:A	PDB 4yea:B
320	0.2591	0.6334	2.812	3.498	65	5	8	0.09231	0	96	7	72	6	PDB 9b0m:A	PDB 5vdf:D
321	0.259	0.1736	2.726	3.431	62	5	6	0.129	0	96	7	67	6	PDB 9b0m:A	PDB 7zc3:B
322	0.2589	0.4727	2.605	3.432	62	6	6	0.129	0	96	7	67	6	PDB 9b0m:A	PDB 4qot:B
323	0.2589	0.4877	2.62	3.432	62	6	6	0.129	0	96	7	67	6	PDB 9b0m:A	PDB 3iwx:B
324	0.2588	0.1999	2.084	3.344	68	5	5	0.01471	0	96	7	83	5	PDB 9b0m:A	PDB 3w66:A
325	0.2587	0.4383	2.56	3.502	65	5	7	0.1077	0	96	7	72	6	PDB 9b0m:A	PDB 3k7r:F
326	0.2587	0.4639	2.127	3.835	75	5	5	0.09333	0	96	7	86	6	PDB 9b0m:A	PDB 7bfg:A
327	0.2587	0.04696	2.082	2.923	66	6	6	0.1212	0	96	7	90	7	PDB 9b0m:A	PDB 4oix:A
328	0.2586	1.39	3.589	2.989	58	5	4	0.1379	0	96	7	68	5	PDB 9b0m:A	PDB 6mrr:A
329	0.2586	0.2115	2.111	3.472	69	5	4	0.01449	0	96	7	82	5	PDB 9b0m:A	PDB 6hhs:F
330	0.2586	0.2305	2.138	3.383	68	5	5	0.01471	0	96	7	82	5	PDB 9b0m:A	PDB 6h84:A
331	0.2585	0.4163	2.052	3.873	75	5	5	0.09333	0	96	7	85	6	PDB 9b0m:A	PDB 7b8v:E
332	0.2584	0.07978	2.217	3.105	68	5	6	0.1176	0	96	7	90	7	PDB 9b0m:A	PDB 2bje:E
333	0.2584	0.224	2.124	3.437	69	5	5	0.01449	0	96	7	83	5	PDB 9b0m:A	PDB 6hhs:D
334	0.2583	0.4273	2.067	3.839	75	5	5	0.09333	0	96	7	86	6	PDB 9b0m:A	PDB 7bfc:I
335	0.2583	0.2672	2.292	3.224	67	5	5	0.0597	0	96	7	84	6	PDB 9b0m:A	PDB 1zpv:B
336	0.2583	0.6012	2.768	3.873	69	5	7	0.1014	0	96	7	72	6	PDB 9b0m:A	PDB 5vdf:A
337	0.2582	0.4922	3.265	3.181	62	5	4	0.08065	0	96	7	73	6	PDB 9b0m:A	PDB 7lmx:B
338	0.2582	0.5275	2.679	3.466	65	5	6	0.1077	0	96	7	73	6	PDB 9b0m:A	PDB 7dv8:M
339	0.2582	0.349	2.426	3.190	67	5	5	0.0597	0	96	7	85	6	PDB 9b0m:A	PDB 1zpv:A
340	0.2582	0.494	2.566	2.843	59	5	5	0.1017	0	96	7	74	5	PDB 9b0m:A	PDB 1th5:A
341	0.2581	0.4261	2.067	3.842	75	5	6	0.09333	0	96	7	86	6	PDB 9b0m:A	PDB 7b90:F
342	0.258	0.02446	2.62	2.745	64	5	6	0.125	0	96	7	90	7	PDB 9b0m:A	PDB 4ojh:B
343	0.2579	0.2827	2.938	3.444	62	5	6	0.129	0	96	7	67	6	PDB 9b0m:A	PDB 4ydx:A
344	0.2577	0.6271	2.812	3.419	64	5	8	0.09375	0	96	7	72	6	PDB 9b0m:A	PDB 3k7r:B
345	0.2576	0.3135	2.99	3.639	64	5	6	0.1406	0	96	7	67	6	PDB 9b0m:A	PDB 1fee:A
346	0.2575	0.0653	2.172	2.936	66	5	6	0.1212	0	96	7	90	7	PDB 9b0m:A	PDB 4ojh:A
347	0.2575	0.9873	3.224	3.108	66	6	5	0.07576	0	96	7	85	6	PDB 9b0m:A	PDB 7b8v:R
348	0.2575	0.1259	1.909	3.396	68	5	5	0.01471	0	96	7	82	5	PDB 9b0m:A	PDB 6h5u:C
349	0.257	0.5905	2.292	3.605	72	5	5	0.08333	0	96	7	86	6	PDB 9b0m:A	PDB 7bfg:I
350	0.2566	0.4818	2.62	3.414	62	6	6	0.129	0	96	7	68	6	PDB 9b0m:A	PDB 1fe4:B
351	0.2564	0.4165	2.53	3.175	67	5	5	0.07463	0	96	7	86	6	PDB 9b0m:A	PDB 7bfg:K
352	0.2563	0.2271	2.138	3.373	68	5	5	0.01471	0	96	7	83	5	PDB 9b0m:A	PDB 6hhs:A
353	0.2562	0.9381	3.108	3.349	59	5	6	0.1864	0	96	7	63	5	PDB 9b0m:A	PDB 4a46:A
354	0.2561	0.9193	3.151	3.088	66	6	5	0.07576	0	96	7	86	6	PDB 9b0m:A	PDB 7bfg:C
355	0.2557	0.247	2.872	3.617	64	5	6	0.125	0	96	7	68	6	PDB 9b0m:A	PDB 5t7l:A
356	0.2554	1.374	3.576	2.937	65	5	5	0.03077	0	96	7	88	5	PDB 9b0m:A	PDB 6sd2:e
357	0.2554	1.374	3.576	2.937	65	5	5	0.03077	0	96	7	88	5	PDB 9b0m:A	PDB 6sd2:D
358	0.2554	1.374	3.576	2.937	65	5	5	0.03077	0	96	7	88	5	PDB 9b0m:A	PDB 6sd2:A
359	0.2554	1.374	3.576	2.937	65	5	5	0.03077	0	96	7	88	5	PDB 9b0m:A	PDB 6sd2:U
360	0.2554	1.374	3.576	2.937	65	5	5	0.03077	0	96	7	88	5	PDB 9b0m:A	PDB 6sd2:T
361	0.2554	1.374	3.576	2.937	65	5	5	0.03077	0	96	7	88	5	PDB 9b0m:A	PDB 6sd2:F
362	0.2554	1.374	3.576	2.937	65	5	5	0.03077	0	96	7	88	5	PDB 9b0m:A	PDB 6sd2:W
363	0.2554	1.374	3.576	2.937	65	5	5	0.03077	0	96	7	88	5	PDB 9b0m:A	PDB 6sd2:I
364	0.2554	1.374	3.576	2.937	65	5	5	0.03077	0	96	7	88	5	PDB 9b0m:A	PDB 6sd2:R
365	0.2554	1.374	3.576	2.937	65	5	5	0.03077	0	96	7	88	5	PDB 9b0m:A	PDB 6sd2:Q
366	0.2554	1.374	3.576	2.937	65	5	5	0.03077	0	96	7	88	5	PDB 9b0m:A	PDB 6sd2:G
367	0.2554	1.374	3.576	2.937	65	5	5	0.03077	0	96	7	88	5	PDB 9b0m:A	PDB 6sd2:f
368	0.2554	1.374	3.576	2.937	65	5	5	0.03077	0	96	7	88	5	PDB 9b0m:A	PDB 6sd2:L
369	0.2554	1.374	3.576	2.937	65	5	5	0.03077	0	96	7	88	5	PDB 9b0m:A	PDB 6sd2:O
370	0.2554	1.374	3.576	2.937	65	5	5	0.03077	0	96	7	88	5	PDB 9b0m:A	PDB 6sd2:Z
371	0.2554	1.374	3.576	2.937	65	5	5	0.03077	0	96	7	88	5	PDB 9b0m:A	PDB 6sd2:b
372	0.2554	1.374	3.576	2.937	65	5	5	0.03077	0	96	7	88	5	PDB 9b0m:A	PDB 6sd2:C
373	0.2554	1.374	3.576	2.937	65	5	5	0.03077	0	96	7	88	5	PDB 9b0m:A	PDB 6sd2:Y
374	0.2554	1.374	3.576	2.937	65	5	5	0.03077	0	96	7	88	5	PDB 9b0m:A	PDB 6sd2:J
375	0.2554	1.374	3.576	2.937	65	5	5	0.03077	0	96	7	88	5	PDB 9b0m:A	PDB 6sd2:N
376	0.255	1.01	2.798	3.596	66	5	5	0.1061	0	96	7	73	6	PDB 9b0m:A	PDB 1fes:A
377	0.2548	0.4274	2.545	3.424	65	5	7	0.1231	0	96	7	75	6	PDB 9b0m:A	PDB 7bnt:B
378	0.2547	0.4076	2.038	3.919	75	5	4	0.09333	0	96	7	85	6	PDB 9b0m:A	PDB 7b8v:B
379	0.2547	0.9258	3.151	3.285	68	6	5	0.07353	0	96	7	86	6	PDB 9b0m:A	PDB 7bfg:B
380	0.2547	1.734	3.922	3.304	69	5	3	0.1739	0	96	7	88	5	PDB 9b0m:A	PDB 1xhj:A
381	0.2544	0.9453	3.18	3.773	65	5	6	0.1692	0	96	7	67	6	PDB 9b0m:A	PDB 1fee:B
382	0.2544	0.01572	1.903	3.207	69	5	7	0.07246	0	96	7	91	7	PDB 9b0m:A	PDB 6krb:G

383	0.2543	0.8076	3.019	3.729	65	6	6	0.1692	0	96	7	68	6	PDB 9b0m:A	PDB 1fe4:A
384	0.2541	0.4083	2.515	3.514	65	5	7	0.1231	0	96	7	73	6	PDB 9b0m:A	PDB 7dv8:I
385	0.2541	0.2184	2.202	3.677	69	5	7	0.1159	0	96	7	78	6	PDB 9b0m:A	PDB 3qsi:F
386	0.254	0.4877	2.62	3.445	62	5	6	0.129	0	96	7	68	6	PDB 9b0m:A	PDB 3cjk:A
387	0.2539	0.8076	3.019	3.588	63	6	6	0.1587	0	96	7	67	6	PDB 9b0m:A	PDB 7zc3:A
388	0.2539	0.6613	2.381	3.627	71	5	6	0.09859	0	96	7	84	6	PDB 9b0m:A	PDB 7bff:A
389	0.2538	0.5568	2.247	3.665	71	5	5	0.08451	0	96	7	83	6	PDB 9b0m:A	PDB 7bff:E
390	0.2536	0.9736	3.209	3.281	65	5	6	0.07692	0	96	7	79	6	PDB 9b0m:A	PDB 5zng:A
391	0.2536	0.2617	2.898	3.547	63	5	5	0.127	0	96	7	68	6	PDB 9b0m:A	PDB 1fe0:B
392	0.2534	0.1785	2.127	3.737	78	5	8	0.1026	0	96	7	98	6	PDB 9b0m:A	PDB 1g1x:F
393	0.2533	0.4594	2.59	3.524	65	5	6	0.1231	0	96	7	73	6	PDB 9b0m:A	PDB 7dv8:E
394	0.2533	0.4489	2.097	3.936	75	5	5	0.08	0	96	7	85	6	PDB 9b0m:A	PDB 7b8v:K
395	0.253	0.5005	2.649	3.806	78	6	6	0.0641	0	96	7	96	6	PDB 9b0m:A	PDB 6dzk:F
396	0.2529	0.3037	2.272	3.252	65	5	4	0.01538	0	96	7	80	5	PDB 9b0m:A	PDB 3w5y:A
397	0.2528	0.3357	2.411	3.348	76	5	7	0.1053	0	96	7	106	7	PDB 9b0m:A	PDB 7boh:F
398	0.2528	0.3357	2.411	3.348	76	5	7	0.1053	0	96	7	106	7	PDB 9b0m:A	PDB 7bog:F
399	0.2528	0.5542	2.709	3.479	64	5	7	0.1094	0	96	7	72	6	PDB 9b0m:A	PDB 3k7r:K
400	0.2527	0.9623	3.195	3.219	67	6	6	0.07463	0	96	7	86	6	PDB 9b0m:A	PDB 7b90:I
401	0.2526	0.2597	1.799	3.693	72	5	5	0.08333	0	96	7	85	6	PDB 9b0m:A	PDB 7b8v:O
402	0.2526	0.9381	3.166	3.668	66	5	7	0.1212	0	96	7	72	6	PDB 9b0m:A	PDB 3k7r:D
403	0.2526	0.0106	1.844	3.028	66	5	7	0.06061	0	96	7	89	7	PDB 9b0m:A	PDB 4hi2:B
404	0.2526	0.2836	1.844	3.945	75	5	5	0.08	0	96	7	85	6	PDB 9b0m:A	PDB 7b8v:X
405	0.2525	0.5031	2.172	3.742	73	5	5	0.09589	0	96	7	86	6	PDB 9b0m:A	PDB 7b90:J
406	0.2525	0.7665	2.975	3.365	61	6	6	0.1148	0	96	7	68	6	PDB 9b0m:A	PDB 5vdf:C
407	0.2524	0.143	2.659	3.678	67	5	4	0.04478	0	96	7	74	6	PDB 9b0m:A	PDB 7lmx:C
408	0.2524	0.8589	3.078	3.312	68	6	5	0.07353	0	96	7	86	6	PDB 9b0m:A	PDB 7b90:B
409	0.2523	0.317	1.903	3.865	74	5	4	0.09459	0	96	7	85	6	PDB 9b0m:A	PDB 7b8v:A
410	0.2521	0.507	2.187	3.748	73	5	6	0.09589	0	96	7	86	6	PDB 9b0m:A	PDB 7b90:G
411	0.252	1.099	3.341	3.823	69	5	4	0.08696	0	96	7	75	6	PDB 9b0m:A	PDB 7lmx:A
412	0.2518	0.4601	2.112	3.871	74	5	5	0.09459	0	96	7	85	6	PDB 9b0m:A	PDB 7b8v:C
413	0.2517	0.1225	1.993	3.343	65	5	7	0.1231	0	96	7	78	6	PDB 9b0m:A	PDB 3lgh:D
414	0.2513	0.4731	2.605	3.424	61	6	6	0.1311	0	96	7	67	6	PDB 9b0m:A	PDB 3iwx:A
415	0.2505	0.4238	2.067	3.877	73	5	6	0.09589	0	96	7	83	6	PDB 9b0m:A	PDB 7bfe:A
416	0.2505	0.2166	2.202	3.682	69	5	7	0.1159	0	96	7	79	6	PDB 9b0m:A	PDB 3qsi:D
417	0.2503	0.528	2.217	3.721	72	5	5	0.09722	0	96	7	85	6	PDB 9b0m:A	PDB 7bff:D
418	0.25	0.6012	2.768	3.795	77	5	6	0.05195	0	96	7	95	6	PDB 9b0m:A	PDB 7mn1:A
419	0.25	0.3941	2.023	3.858	74	5	5	0.09459	0	96	7	86	6	PDB 9b0m:A	PDB 7bfc:C
420	0.25	0.6217	2.798	3.471	64	5	6	0.09375	0	96	7	73	6	PDB 9b0m:A	PDB 7dv8:K
421	0.2499	0.7326	2.93	3.539	62	5	6	0.1452	0	96	7	67	6	PDB 9b0m:A	PDB 4yea:A
422	0.2499	1.239	3.063	3.198	66	5	6	0.06061	0	96	7	85	6	PDB 9b0m:A	PDB 7b8v:D
423	0.2498	0.01748	1.918	3.173	68	5	6	0.07353	0	96	7	91	7	PDB 9b0m:A	PDB 6krb:A
424	0.2496	0.6023	2.306	3.693	72	5	6	0.1111	0	96	7	86	6	PDB 9b0m:A	PDB 7b90:D
425	0.2495	0.4786	3.239	3.400	61	5	5	0.09836	0	96	7	68	6	PDB 9b0m:A	PDB 1tl5:A
426	0.2495	1.576	3.413	3.111	65	5	4	0.07692	0	96	7	85	6	PDB 9b0m:A	PDB 7b8v:U
427	0.2491	0.5065	2.649	3.480	64	5	6	0.125	0	96	7	73	6	PDB 9b0m:A	PDB 7dv8:F
428	0.2491	0.01449	1.888	3.182	68	5	6	0.07353	0	96	7	91	7	PDB 9b0m:A	PDB 6krb:J
429	0.249	0.2184	2.202	3.963	81	5	7	0.09877	0	96	7	100	6	PDB 9b0m:A	PDB 6xe0:E
430	0.2489	0.4048	2.038	3.823	73	5	6	0.08219	0	96	7	85	6	PDB 9b0m:A	PDB 7b8v:P
431	0.2489	0.4272	2.067	3.907	74	5	5	0.09459	0	96	7	85	6	PDB 9b0m:A	PDB 7bfc:M
432	0.2488	0.4309	2.545	3.715	66	5	6	0.07576	0	96	7	72	6	PDB 9b0m:A	PDB 1aw0:A
433	0.2486	0.524	2.202	3.705	72	5	6	0.09722	0	96	7	86	6	PDB 9b0m:A	PDB 7bfg:G
434	0.2484	0.8657	3.092	3.216	66	6	6	0.07576	0	96	7	85	6	PDB 9b0m:A	PDB 7bfe:D
435	0.2484	0.3867	2.008	3.914	74	5	6	0.09459	0	96	7	85	6	PDB 9b0m:A	PDB 7bfc:K
436	0.2482	0.7902	3.004	3.709	64	5	6	0.1719	0	96	7	68	6	PDB 9b0m:A	PDB 7dc1:A
437	0.2481	0.3991	2.023	3.881	74	5	5	0.09459	0	96	7	86	6	PDB 9b0m:A	PDB 7bfc:B
438	0.2479	0.09394	2.526	3.642	79	5	7	0.1013	0	96	7	106	6	PDB 9b0m:A	PDB 7nax:F
439	0.2479	0.09394	2.526	3.642	79	5	7	0.1013	0	96	7	106	6	PDB 9b0m:A	PDB 7bof:F
440	0.2479	0.07855	2.217	3.141	67	5	7	0.01493	0	96	7	90	7	PDB 9b0m:A	PDB 3br8:A
441	0.2478	0.06875	2.187	3.298	68	5	6	0.05882	0	96	7	88	7	PDB 9b0m:A	PDB 4hi2:A
442	0.2478	0.6964	2.951	3.277	60	5	7	0.08333	0	96	7	69	5	PDB 9b0m:A	PDB 1fwp:A
443	0.2476	0.7269	2.93	3.370	68	6	5	0.07353	0	96	7	86	6	PDB 9b0m:A	PDB 7bff:B
444	0.2475	0.2648	2.292	3.719	69	6	7	0.07246	0	96	7	79	6	PDB 9b0m:A	PDB 7mpw:E
445	0.2474	0.2184	2.202	3.682	69	5	7	0.1159	0	96	7	80	6	PDB 9b0m:A	PDB 3qsi:H
446	0.2473	0.005241	1.754	3.092	66	5	6	0.06061	0	96	7	89	7	PDB 9b0m:A	PDB 4hi2:K
447	0.2471	0.1837	2.142	3.724	69	6	7	0.07246	0	96	7	79	6	PDB 9b0m:A	PDB 7mpw:C
448	0.247	0.3991	2.023	3.846	73	5	5	0.09589	0	96	7	85	6	PDB 9b0m:A	PDB 7b8v:I
449	0.247	0.4691	2.127	3.640	71	5	5	0.08451	0	96	7	86	6	PDB 9b0m:A	PDB 7bfg:H
450	0.2469	0.3674	2.456	3.550	64	6	5	0.0625	0	96	7	72	6	PDB 9b0m:A	PDB 2aw0:A
451	0.2468	0.3762	1.993	3.933	74	5	6	0.09459	0	96	7	85	6	PDB 9b0m:A	PDB 7bfe:C
452	0.2467	0.5186	2.202	3.851	73	5	6	0.09589	0	96	7	85	6	PDB 9b0m:A	PDB 7bfe:F
453	0.2466	0.2624	2.292	3.224	67	5	5	0.0597	0	96	7	88	6	PDB 9b0m:A	PDB 1zpv:C
454	0.2465	1.036	3.268	3.729	64	6	6	0.1719	0	96	7	68	6	PDB 9b0m:A	PDB 5f0w:C
455	0.2464	0.3834	2.008	3.902	74	5	6	0.09459	0	96	7	86	6	PDB 9b0m:A	PDB 7bfc:L
456	0.2464	0.5735	2.738	3.410	74	5	7	0.09459	0	96	7	101	6	PDB 9b0m:A	PDB 1i94:F
457	0.2462	0.5363	2.694	3.733	64	6	6	0.1562	0	96	7	68	6	PDB 9b0m:A	PDB 5f0w:B
458	0.246	0.5124	2.187	3.738	72	5	6	0.09722	0	96	7	86	6	PDB 9b0m:A	PDB 7bfg:F
459	0.2459	0.1224	2.686	3.545	66	5	6	0.04545	0	96	7	77	7	PDB 9b0m:A	PDB 5fii:A
460	0.2458	0.6721	2.857	3.738	64	6	6	0.1562	0	96	7	68	6	PDB 9b0m:A	PDB 5f0w:D
461	0.2458	0.4349	2.082	3.862	73	5	6	0.09589	0	96	7	85	6	PDB 9b0m:A	PDB 7bfc:J
462	0.2456	0.4489	2.097	3.657	71	5	5	0.08451	0	96	7	86	6	PDB 9b0m:A	PDB 7b90:H
463	0.2456	0.5423	2.694	3.740	64	6	6	0.1562	0	96	7	68	6	PDB 9b0m:A	PDB 5f0w:A
464	0.2452	0.005807	1.769	3.050	66	5	6	0.07576	0	96	7	91	7	PDB 9b0m:A	PDB 6krb:B
465	0.2451	0.08575	1.888	3.546	68	5	5	0.1029	0	96	7	82	6	PDB 9b0m:A	PDB 4rbt:C
466	0.245	0.6439	2.351	3.232	68	5	7	0.05882	0	96	7	91	6	PDB 9b0m:A	PDB 2yy3:A
467	0.2448	0.468	2.127	3.874	73	5	5	0.08219	0	96	7	85	6	PDB 9b0m:A	PDB 7b8v:J
468	0.2447	0.2672	2.292	3.715	69	5	7	0.07246	0	96	7	80	6	PDB 9b0m:A	PDB 7mpx:A
469	0.2446	0.1183	1.978	3.753	78	6	7	0.08974	0	96	7	101	6	PDB 9b0m:A	PDB 2uxc:F
470	0.2445	0.272	2.306	3.538	67	6	6	0.07463	0	96	7	80	6	PDB 9b0m:A	PDB 7mpv:C
471	0.2443	0.1671	1.873	3.429	75	5	7	0.09333	0	96	7	104	7	PDB 9b0m:A	PDB 6w77:F
472	0.244	0.5462	2.709	3.352	62	5	6	0.1129	0	96	7	73	6	PDB 9b0m:A	PDB 7dv8:N
473	0.2438	0.08627	1.888	3.370	60	5	9	0.01667	0	96	7	68	6	PDB 9b0m:A	PDB 1ffw:B
474	0.2437	0.02362	1.963	3.158	67	5	7	0.07463	0	96	7	91	7	PDB 9b0m:A	PDB 4ojg:A
475	0.2437	0.5084	2.649	3.661	62	6	9	0.06452	0	96	7	66	6	PDB 9b0m:A	PDB 2rog:A
476	0.2436	0.4885	2.157	3.768	72	5	5	0.09722	0	96	7	86	6	PDB 9b0m:A	PDB 7bfg:J
477	0.2431	0.293	2.336	3.569	61	5	6	0.09836	0	96	7	66	6	PDB 9b0m:A	PDB 4a48:A
478	0.243	0.4977	2.172	3.945	74	5	5	0.09459	0	96	7	86	6	PDB 9b0m:A	PDB 7bfc:N
479	0.2427	0.2187	2.819	3.560	64	5	7	0.09375	0	96	7	73	6	PDB 9b0m:A	PDB 1fd8:A

480	0.2427	0.6597	2.842	3.495	62	5	5	0.1129	0	96	7	70	6	PDB 9b0m:A	PDB 5vdf:F
481	0.2427	1.112	3.434	3.493	65	5	5	0.06154	0	96	7	77	5	PDB 9b0m:A	PDB 1y3k:A
482	0.2426	0.2535	2.277	3.741	69	5	7	0.07246	0	96	7	80	6	PDB 9b0m:A	PDB 7mpx:B
483	0.2424	0.375	1.993	3.868	73	5	6	0.09589	0	96	7	86	6	PDB 9b0m:A	PDB 7bfc:H
484	0.2423	0.2512	2.262	3.745	69	5	7	0.07246	0	96	7	80	6	PDB 9b0m:A	PDB 7mpx:F
485	0.2422	0.4805	2.142	3.955	74	5	5	0.09459	0	96	7	86	6	PDB 9b0m:A	PDB 7bfc:D
486	0.2421	0.4079	2.515	3.567	64	5	6	0.125	0	96	7	73	6	PDB 9b0m:A	PDB 7dv8:D
487	0.2421	0.331	2.396	3.567	64	5	6	0.125	0	96	7	73	6	PDB 9b0m:A	PDB 7dv8:G
488	0.2421	0.1952	2.157	3.697	68	5	6	0.07353	0	96	7	79	6	PDB 9b0m:A	PDB 3r2d:J
489	0.2421	0.2503	1.829	3.785	78	5	7	0.05128	0	96	7	101	6	PDB 9b0m:A	PDB 4dv1:F
490	0.2421	0.2493	2.262	3.748	69	5	7	0.07246	0	96	7	80	6	PDB 9b0m:A	PDB 7mpx:E
491	0.242	0.1759	1.636	3.824	72	5	7	0.08333	0	96	7	85	6	PDB 9b0m:A	PDB 7b8v:N
492	0.242	0.2648	2.292	3.711	69	6	7	0.07246	0	96	7	81	6	PDB 9b0m:A	PDB 7mpw:D
493	0.2418	0.01028	1.844	3.000	65	5	7	0.06154	0	96	7	91	7	PDB 9b0m:A	PDB 6krb:K
494	0.2416	0.4299	2.545	3.442	60	5	6	0.1167	0	96	7	67	6	PDB 9b0m:A	PDB 5vdf:G
495	0.2414	0.3007	2.351	3.719	69	6	6	0.07246	0	96	7	81	6	PDB 9b0m:A	PDB 7mpx:C
496	0.2411	0.006417	1.784	3.100	66	5	7	0.06061	0	96	7	91	7	PDB 9b0m:A	PDB 6krb:I
497	0.2409	0.2582	2.277	3.724	69	5	7	0.07246	0	96	7	81	6	PDB 9b0m:A	PDB 7mpv:I
498	0.2409	0.3807	2.471	3.582	64	5	6	0.125	0	96	7	73	6	PDB 9b0m:A	PDB 7dv8:C
499	0.2409	0.008764	1.829	3.103	66	5	7	0.06061	0	96	7	91	7	PDB 9b0m:A	PDB 6krb:E
500	0.2408	0.1802	2.127	3.726	69	6	7	0.07246	0	96	7	81	6	PDB 9b0m:A	PDB 7mpw:B
501	0.2406	0.8896	2.664	3.618	63	5	5	0.09524	0	96	7	70	6	PDB 9b0m:A	PDB 7qpx:D
502	0.2406	0.2582	2.277	3.729	69	5	7	0.07246	0	96	7	81	6	PDB 9b0m:A	PDB 7mpx:D
503	0.2404	0.4409	2.812	3.419	75	6	8	0.1067	0	96	7	106	8	PDB 9b0m:A	PDB 7bod:F
504	0.2404	0.4409	2.812	3.419	75	6	8	0.1067	0	96	7	106	8	PDB 9b0m:A	PDB 7boe:F
505	0.2403	0.06885	1.829	3.445	65	5	6	0.06154	0	96	7	79	6	PDB 9b0m:A	PDB 3qsi:G
506	0.2402	0.01713	1.903	2.960	64	5	7	0.07812	0	96	7	90	7	PDB 9b0m:A	PDB 2bje:C
507	0.24	0.1415	2.038	3.540	66	6	6	0.07576	0	96	7	79	6	PDB 9b0m:A	PDB 3qsi:I
508	0.24	0.006357	1.784	3.022	65	5	6	0.07692	0	96	7	91	7	PDB 9b0m:A	PDB 6krb:D
509	0.24	0.4989	2.172	3.850	72	5	6	0.09722	0	96	7	85	6	PDB 9b0m:A	PDB 7b8v:S
510	0.2399	0.524	2.202	3.815	72	5	5	0.09722	0	96	7	86	6	PDB 9b0m:A	PDB 7bfc:E
511	0.2398	0.4707	2.605	3.404	62	5	6	0.129	0	96	7	73	6	PDB 9b0m:A	PDB 7dv8:A
512	0.2398	0.806	3.019	3.501	63	5	6	0.127	0	96	7	73	6	PDB 9b0m:A	PDB 7dv8:B
513	0.2392	0.1885	2.057	3.459	69	5	4	0.01449	0	96	7	89	5	PDB 9b0m:A	PDB 6h5m:A
514	0.239	0.4594	2.59	3.511	63	5	6	0.127	0	96	7	73	6	PDB 9b0m:A	PDB 7dv8:J
515	0.239	0.1856	2.142	3.350	63	5	7	0.09524	0	96	7	77	6	PDB 9b0m:A	PDB 7nmm:C
516	0.2387	0.0009895	2.245	3.423	73	5	7	0.08219	0	96	7	101	7	PDB 9b0m:A	PDB 4jya:F
517	0.2385	0.0693	2.097	3.524	76	5	7	0.1053	0	96	7	106	7	PDB 9b0m:A	PDB 5no2:F
518	0.2383	0.318	2.381	3.324	61	5	6	0.09836	0	96	7	73	6	PDB 9b0m:A	PDB 5zne:A
519	0.2383	0.2424	2.247	3.358	63	6	8	0.1111	0	96	7	77	6	PDB 9b0m:A	PDB 3ibw:B
520	0.238	0.8412	3.063	3.329	61	5	6	0.1148	0	96	7	73	6	PDB 9b0m:A	PDB 7dvg:A
521	0.238	0.4077	2.038	3.926	73	5	6	0.09589	0	96	7	86	6	PDB 9b0m:A	PDB 7bfc:G
522	0.2378	0.03969	1.71	3.134	67	5	7	0.01493	0	96	7	94	7	PDB 9b0m:A	PDB 2vh7:A
523	0.2376	0.7068	2.441	3.405	67	5	6	0.08955	0	96	7	86	6	PDB 9b0m:A	PDB 7bfg:D
524	0.2376	0.9164	2.694	3.805	65	5	4	0.09231	0	96	7	71	6	PDB 9b0m:A	PDB 7a8x:D
525	0.2375	0.3592	2.441	3.620	69	5	4	0.1159	0	96	7	85	7	PDB 9b0m:A	PDB 4p7t:A
526	0.2374	0.4055	2.819	3.077	67	5	5	0.04478	0	96	7	96	6	PDB 9b0m:A	PDB 6g6i:A
527	0.2372	0.1087	1.963	3.760	68	6	4	0.07353	0	96	7	79	6	PDB 9b0m:A	PDB 3lgh:C
528	0.2369	0.2512	2.262	3.753	70	5	7	0.07143	0	96	7	84	6	PDB 9b0m:A	PDB 7mpv:H
529	0.2366	0.3626	2.5	4.123	72	5	7	0.05556	0	96	7	79	7	PDB 9b0m:A	PDB 5fii:B
530	0.2365	0.4857	2.62	3.348	61	5	7	0.08197	0	96	7	73	6	PDB 9b0m:A	PDB 7dv8:L
531	0.2364	0.3041	1.873	4.031	74	5	6	0.09459	0	96	7	86	6	PDB 9b0m:A	PDB 7bff:C
532	0.2363	0.3335	1.859	3.700	76	5	4	0.1316	0	96	7	101	6	PDB 9b0m:A	PDB 2od4:A
533	0.2362	0.2397	2.545	3.772	68	5	4	0.05882	0	96	7	79	7	PDB 9b0m:A	PDB 3ofh:B
534	0.2361	0.7134	2.845	3.398	58	5	5	0.1379	0	96	7	65	5	PDB 9b0m:A	PDB 5f0u:A
535	0.2359	0.2672	2.292	3.961	65	6	6	0.1385	0	96	7	68	6	PDB 9b0m:A	PDB 2k1r:B
536	0.2354	0.5568	2.247	3.700	70	5	5	0.08571	0	96	7	86	6	PDB 9b0m:A	PDB 7bfc:A
537	0.2351	0.2716	2.218	3.368	67	5	5	0.01493	0	96	7	88	5	PDB 9b0m:A	PDB 6hhs:B
538	0.235	0.01448	1.859	3.302	67	5	6	0.0597	0	96	7	90	7	PDB 9b0m:A	PDB 3tnv:A
539	0.2349	0.04525	2.326	3.277	58	5	6	0.03448	0	96	7	68	6	PDB 9b0m:A	PDB 1ffw:D
540	0.2346	0.4451	2.575	3.348	64	5	7	0.1562	0	96	7	81	6	PDB 9b0m:A	PDB 6fub:A
541	0.2346	1.262	3.46	3.028	58	5	5	0.06897	0	96	7	74	5	PDB 9b0m:A	PDB 3zzp:A
542	0.2346	0.2367	2.247	3.676	70	5	4	0.05714	0	96	7	87	7	PDB 9b0m:A	PDB 4ox8:C
543	0.2344	0.2658	2.911	3.316	60	5	4	0.08333	0	96	7	72	6	PDB 9b0m:A	PDB 1fvq:A
544	0.2344	0.2981	2.351	3.571	63	5	6	0.127	0	96	7	73	6	PDB 9b0m:A	PDB 7dv8:H
545	0.2342	0.0421	1.725	3.148	67	5	7	0.01493	0	96	7	95	7	PDB 9b0m:A	PDB 2w4c:A
546	0.2342	0.01361	1.888	2.779	63	5	6	0.09524	0	96	7	95	7	PDB 9b0m:A	PDB 4oj3:B
547	0.2338	0.2501	2.262	3.772	71	5	4	0.05634	0	96	7	87	7	PDB 9b0m:A	PDB 4ox8:B
548	0.2337	0.2038	2.172	3.043	64	5	5	0.0625	0	96	7	90	7	PDB 9b0m:A	PDB 4oj1:A
549	0.2336	0.07915	2.097	3.398	59	5	5	0.1356	0	96	7	68	6	PDB 9b0m:A	PDB 5hl8:D
550	0.2335	0.1162	1.882	3.275	65	5	5	0.04615	0	96	7	86	5	PDB 9b0m:A	PDB 6vda:A
551	0.2335	0.09061	1.903	3.399	64	6	6	0.0625	0	96	7	80	6	PDB 9b0m:A	PDB 3qsi:E
552	0.2333	0.3011	2.351	3.289	60	5	7	0.06667	0	96	7	73	6	PDB 9b0m:A	PDB 7dvg:B
553	0.2333	0.349	2.426	3.532	67	5	4	0.1194	0	96	7	84	6	PDB 9b0m:A	PDB 4ppd:E
554	0.2333	0.07334	1.844	3.496	65	6	6	0.07692	0	96	7	80	6	PDB 9b0m:A	PDB 3qsi:C
555	0.2328	0.01988	1.933	2.926	63	5	7	0.04762	0	96	7	91	7	PDB 9b0m:A	PDB 7chx:A
556	0.2327	0.07334	1.844	3.504	65	6	6	0.07692	0	96	7	80	6	PDB 9b0m:A	PDB 3qsi:A
557	0.2323	0.2642	2.292	3.509	67	6	5	0.08955	0	96	7	85	7	PDB 9b0m:A	PDB 7mmx:F
558	0.2322	0.3757	2.471	3.292	65	5	6	0.07692	0	96	7	86	6	PDB 9b0m:A	PDB 7bfe:B
559	0.2321	0.1697	2.017	3.483	69	5	4	0.01449	0	96	7	91	5	PDB 9b0m:A	PDB 6h5u:B
560	0.2318	0.175	2.112	3.318	58	5	6	0.03448	0	96	7	68	6	PDB 9b0m:A	PDB 1ffs:B
561	0.2317	0.2346	2.232	3.517	67	6	5	0.1194	0	96	7	85	7	PDB 9b0m:A	PDB 5l38:E
562	0.2317	0.4362	2.56	3.555	65	6	8	0.07692	0	96	7	79	6	PDB 9b0m:A	PDB 1kvj:A
563	0.2315	0.116	1.978	3.537	66	6	4	0.1061	0	96	7	82	6	PDB 9b0m:A	PDB 4rbt:B
564	0.2314	0.23	2.232	3.717	70	5	4	0.05714	0	96	7	87	7	PDB 9b0m:A	PDB 4ox8:D
565	0.2312	0.2524	2.277	3.488	67	5	5	0.1045	0	96	7	86	7	PDB 9b0m:A	PDB 4axj:C
566	0.2311	0.3203	2.381	3.327	58	5	6	0.03448	0	96	7	68	6	PDB 9b0m:A	PDB 1ffs:D
567	0.231	0.4393	2.023	2.729	58	5	7	0.2241	0	96	7	83	6	PDB 9b0m:A	PDB 3dfe:D
568	0.2309	0.03802	1.963	4.204	79	5	7	0.1392	0	96	7	95	7	PDB 9b0m:A	PDB 5b0a:A
569	0.2307	0.5714	2.738	3.474	64	5	7	0.1406	0	96	7	79	6	PDB 9b0m:A	PDB 6lxg:B
570	0.2306	0.3347	2.411	3.496	67	6	5	0.1194	0	96	7	86	7	PDB 9b0m:A	PDB 5l38:K
571	0.2305	0.3538	2.441	3.498	67	6	5	0.1194	0	96	7	86	6	PDB 9b0m:A	PDB 5l38:B
572	0.2304	0.1198	2.306	3.178	65	5	5	0.01538	0	96	7	90	7	PDB 9b0m:A	PDB 2w4d:F
573	0.2303	0.05703	2.379	3.126	56	5	6	0.01786	0	96	7	68	6	PDB 9b0m:A	PDB 1ffg:B
574	0.23	0.2614	2.292	3.648	69	5	4	0.05797	0	96	7	87	7	PDB 9b0m:A	PDB 4ox8:F
575	0.23	0.004841	1.754	3.058	64	5	6	0.0625	0	96	7	91	7	PDB 9b0m:A	PDB 6krb:H
576	0.2299	0.5696	2.738	3.840	62	5	7	0.1613	0	96	7	66	6	PDB 9b0m:A	PDB 7dc1:B

577	0.2298	0.1109	1.963	3.938	62	5	6	0.08065	0	96	7	64	6	PDB 9b0m:A	PDB 6u2d:D
578	0.2298	0.06314	1.799	3.337	64	6	5	0.07812	0	96	7	83	6	PDB 9b0m:A	PDB 3bkt:B
579	0.2297	0.04837	1.754	3.488	66	5	4	0.06061	0	96	7	84	7	PDB 9b0m:A	PDB 4qif:I
580	0.2296	0.3203	2.441	4.211	71	5	5	0.07042	0	96	7	77	7	PDB 9b0m:A	PDB 5fii:C
581	0.2295	0.23	2.232	3.742	70	5	4	0.05714	0	96	7	87	7	PDB 9b0m:A	PDB 4ox8:E
582	0.2295	0.2239	2.545	3.440	67	5	5	0.0597	0	96	7	88	7	PDB 9b0m:A	PDB 4hi2:G
583	0.2294	0.01025	2.057	3.791	76	5	10	0.06579	0	96	7	101	6	PDB 9b0m:A	PDB 1xmq:F
584	0.2292	0.2085	2.097	3.521	69	5	4	0.01449	0	96	7	91	5	PDB 9b0m:A	PDB 6gp6:B
585	0.2292	0.2493	2.381	3.006	63	5	5	0.06349	0	96	7	90	7	PDB 9b0m:A	PDB 2bje:A
586	0.2292	0.2638	2.292	3.514	67	6	5	0.08955	0	96	7	86	7	PDB 9b0m:A	PDB 7mmx:D
587	0.2292	0.1595	2.082	3.526	61	5	7	0.08197	0	96	7	71	6	PDB 9b0m:A	PDB 6g10:A
588	0.2292	0.2494	2.885	4.001	75	5	6	0.05333	0	96	7	92	6	PDB 9b0m:A	PDB 2rrn:A
589	0.2291	0.1631	2.097	3.752	68	6	6	0.1029	0	96	7	82	6	PDB 9b0m:A	PDB 4ppd:C
590	0.2289	0.07517	2.459	3.335	65	5	4	0.06154	0	96	7	86	7	PDB 9b0m:A	PDB 4p7t:D
591	0.2287	0.3046	2.366	3.521	67	5	4	0.1194	0	96	7	86	7	PDB 9b0m:A	PDB 4p7t:E
592	0.2286	0.1631	2.097	3.595	67	6	5	0.08955	0	96	7	84	6	PDB 9b0m:A	PDB 7mpv:J
593	0.2285	0.1651	2.097	3.595	67	6	5	0.08955	0	96	7	84	6	PDB 9b0m:A	PDB 7mpv:K
594	0.2285	0.05535	1.784	3.307	69	5	7	0.04348	0	96	7	98	7	PDB 9b0m:A	PDB 2acy:A
595	0.2283	0.02362	1.963	2.889	62	5	7	0.03226	0	96	7	91	7	PDB 9b0m:A	PDB 7chx:B
596	0.2282	0.06203	2.779	3.480	68	5	5	0.04412	0	96	7	90	7	PDB 9b0m:A	PDB 2w4d:A
597	0.2281	0.2579	2.326	3.357	60	5	5	0.1333	0	96	7	73	5	PDB 9b0m:A	PDB 1k0v:A
598	0.228	0.05884	2.766	3.483	68	5	5	0.04412	0	96	7	90	7	PDB 9b0m:A	PDB 2w4d:D
599	0.2279	2.254	4.488	2.663	65	5	5	0.1385	0	96	7	108	6	PDB 9b0m:A	PDB 2kdn:A
600	0.2278	0.1917	2.157	3.588	68	5	5	0.1029	0	96	7	87	6	PDB 9b0m:A	PDB 4rbv:E
601	0.2276	0.03447	1.68	3.203	67	5	7	0.01493	0	96	7	96	7	PDB 9b0m:A	PDB 2w4p:A
602	0.2275	0.1505	1.963	3.455	68	5	4	0.01471	0	96	7	91	5	PDB 9b0m:A	PDB 6h5k:A
603	0.2273	0.2716	2.218	3.625	74	5	3	0.08108	0	96	7	102	5	PDB 9b0m:A	PDB 3ueb:B
604	0.2272	0.004786	2.339	3.221	65	5	6	0.07692	0	96	7	90	7	PDB 9b0m:A	PDB 2w4d:E
605	0.2272	0.2876	2.336	3.471	67	6	5	0.1194	0	96	7	88	7	PDB 9b0m:A	PDB 5l38:H
606	0.227	0.2101	2.187	3.653	69	5	5	0.1014	0	96	7	88	6	PDB 9b0m:A	PDB 4rbu:C
607	0.2269	0.09208	1.918	3.233	64	5	4	0.07812	0	96	7	87	7	PDB 9b0m:A	PDB 4rbu:H
608	0.2269	0.001839	1.287	4.074	73	5	6	0.09589	0	96	7	86	6	PDB 9b0m:A	PDB 3mgj:B
609	0.2269	0.331	2.396	3.510	67	6	5	0.1194	0	96	7	87	6	PDB 9b0m:A	PDB 5l38:I
610	0.2268	0.1	2.54	3.527	66	5	4	0.07576	0	96	7	84	7	PDB 9b0m:A	PDB 4p7t:F
611	0.2264	0.02446	1.621	3.367	65	5	4	0.06154	0	96	7	86	7	PDB 9b0m:A	PDB 4qie:E
612	0.2261	0.03762	2.052	3.014	63	5	6	0.01587	0	96	7	91	7	PDB 9b0m:A	PDB 2fhm:A
613	0.226	0.1996	2.172	3.911	81	6	4	0.1111	0	96	7	112	6	PDB 9b0m:A	PDB 2mq8:A
614	0.2258	0.03742	2.023	3.145	64	5	5	0	0	96	7	90	7	PDB 9b0m:A	PDB 2w4d:C
615	0.2258	1.004	2.798	3.586	62	5	4	0.09677	0	96	7	73	6	PDB 9b0m:A	PDB 6q76:A
616	0.2258	0.0793	1.873	3.180	64	5	4	0.04688	0	96	7	89	7	PDB 9b0m:A	PDB 4rbv:G
617	0.2258	0.1631	2.097	3.596	67	6	5	0.08955	0	96	7	85	6	PDB 9b0m:A	PDB 7mpv:L
618	0.2257	0.001985	1.855	3.225	63	5	4	0.04762	0	96	7	85	7	PDB 9b0m:A	PDB 4qie:I
619	0.2256	0.08397	1.873	3.760	75	6	9	0.06667	0	96	7	101	6	PDB 9b0m:A	PDB 7v2q:F
620	0.2256	0.1207	1.993	3.343	65	5	4	0.07692	0	96	7	87	7	PDB 9b0m:A	PDB 4rbu:A
621	0.2256	0.1442	2.052	3.392	64	6	4	0.09375	0	96	7	83	7	PDB 9b0m:A	PDB 4qie:G
622	0.2255	0.001585	1.621	3.426	67	5	6	0.0597	0	96	7	90	7	PDB 9b0m:A	PDB 4oj3:A
623	0.2253	0.06885	2.157	3.245	65	5	5	0.01538	0	96	7	90	7	PDB 9b0m:A	PDB 2w4d:B
624	0.2253	0.2305	2.232	3.695	70	5	4	0.05714	0	96	7	90	7	PDB 9b0m:A	PDB 4ox8:A
625	0.2251	0.02401	1.621	3.197	63	5	4	0.04762	0	96	7	86	7	PDB 9b0m:A	PDB 4ppd:G
626	0.2251	0.3908	2.419	3.300	67	5	5	0.01493	0	96	7	94	5	PDB 9b0m:A	PDB 6han:A
627	0.2249	0.03052	2.259	3.294	64	5	4	0.04688	0	96	7	86	7	PDB 9b0m:A	PDB 4p7v:A
628	0.2249	0.1319	2.023	3.516	66	6	5	0.09091	0	96	7	85	6	PDB 9b0m:A	PDB 7mpv:A
629	0.2247	0.03193	2.285	3.119	68	5	4	0.07353	0	96	7	103	6	PDB 9b0m:A	PDB 3ueb:C
630	0.2247	0.008379	1.784	3.346	66	5	7	0.07576	0	96	7	90	7	PDB 9b0m:A	PDB 1w2i:B
631	0.2247	0.1867	2.142	3.755	69	6	7	0.1159	0	96	7	86	7	PDB 9b0m:A	PDB 5l38:L
632	0.2246	0.07517	2.459	3.392	65	5	4	0.04615	0	96	7	86	7	PDB 9b0m:A	PDB 7mmx:A
633	0.2245	0.1837	2.142	3.429	65	6	5	0.07692	0	96	7	85	6	PDB 9b0m:A	PDB 4rbv:C
634	0.224	0.1521	2.067	3.693	69	6	5	0.1014	0	96	7	88	6	PDB 9b0m:A	PDB 4qig:B
635	0.2238	0.0594	2.406	3.083	62	5	4	0.03226	0	96	7	87	7	PDB 9b0m:A	PDB 4axj:A
636	0.2238	0.349	2.426	3.482	67	6	5	0.1194	0	96	7	89	6	PDB 9b0m:A	PDB 5l38:D
637	0.2238	0.04139	1.725	3.439	65	5	4	0.04615	0	96	7	85	7	PDB 9b0m:A	PDB 4qie:C
638	0.2231	0.01584	2.138	3.295	63	5	5	0.04762	0	96	7	84	7	PDB 9b0m:A	PDB 4qif:G
639	0.2231	0.01482	1.533	3.190	63	5	4	0.04762	0	96	7	87	7	PDB 9b0m:A	PDB 4ppd:D
640	0.223	0.1631	2.097	3.305	58	5	6	0.1379	0	96	7	71	6	PDB 9b0m:A	PDB 7bnt:A
641	0.2227	0.3752	2.471	3.497	67	5	4	0.1194	0	96	7	89	6	PDB 9b0m:A	PDB 4qie:H
642	0.2226	0.2414	2.247	3.498	67	6	5	0.1194	0	96	7	89	7	PDB 9b0m:A	PDB 5l38:J
643	0.2225	0.05239	2.379	3.513	66	5	4	0.07576	0	96	7	86	7	PDB 9b0m:A	PDB 4p7t:B
644	0.2223	0.1109	1.963	3.906	63	6	6	0.09524	0	96	7	69	6	PDB 9b0m:A	PDB 6xfu:B
645	0.2222	0.07517	2.459	3.296	64	5	4	0.04688	0	96	7	87	7	PDB 9b0m:A	PDB 7mmx:E
646	0.2222	0.06469	1.814	3.718	69	5	6	0.1014	0	96	7	88	6	PDB 9b0m:A	PDB 4qie:A
647	0.2222	0.1856	2.142	4.041	82	6	4	0.1098	0	96	7	112	6	PDB 9b0m:A	PDB 2n76:A
648	0.222	0.2367	2.247	3.760	71	5	5	0.05634	0	96	7	92	7	PDB 9b0m:A	PDB 8b12:B
649	0.2217	0.01683	2.151	3.243	63	5	4	0.04762	0	96	7	86	7	PDB 9b0m:A	PDB 4qif:E
650	0.2215	0.1424	2.112	3.787	66	5	5	0.06061	0	96	7	79	7	PDB 9b0m:A	PDB 5fii:D
651	0.2214	0.106	1.948	3.730	69	6	7	0.1159	0	96	7	88	6	PDB 9b0m:A	PDB 4qif:D
652	0.2214	0.1542	2.686	3.661	69	5	5	0.08696	0	96	7	90	7	PDB 9b0m:A	PDB 5l38:F
653	0.2213	0.1087	1.963	3.517	67	5	4	0.1194	0	96	7	89	6	PDB 9b0m:A	PDB 4rbt:A
654	0.2211	0.2047	2.187	3.773	71	5	4	0.05634	0	96	7	92	7	PDB 9b0m:A	PDB 8b11:D
655	0.2209	1.623	3.82	3.526	70	5	4	0.1143	0	96	7	97	5	PDB 9b0m:A	PDB 2m5o:A
656	0.2209	0.0009487	2.165	3.117	63	5	6	0.04762	0	96	7	90	7	PDB 9b0m:A	PDB 1v3z:B
657	0.2209	0.003659	1.68	3.231	59	6	6	0.05085	0	96	7	76	7	PDB 9b0m:A	PDB 4rwx:A
658	0.2208	0.1164	1.978	3.757	70	5	6	0.08571	0	96	7	90	7	PDB 9b0m:A	PDB 3dnc:A
659	0.2208	0.09874	1.933	3.738	69	6	7	0.1014	0	96	7	88	6	PDB 9b0m:A	PDB 4qie:D
660	0.2208	0.1047	1.948	3.221	63	5	4	0.04762	0	96	7	87	7	PDB 9b0m:A	PDB 4qig:G
661	0.2206	0.06054	1.799	3.760	70	6	6	0.05714	0	96	7	90	7	PDB 9b0m:A	PDB 3mpw:H
662	0.2203	0.03911	1.695	3.791	62	5	7	0.1129	0	96	7	70	6	PDB 9b0m:A	PDB 6xfu:A
663	0.2202	0.1761	2.127	3.264	60	5	4	0.15	0	96	7	78	6	PDB 9b0m:A	PDB 7nlj:A
664	0.2201	0.23	2.232	3.700	70	5	4	0.05714	0	96	7	92	7	PDB 9b0m:A	PDB 8b12:D
665	0.2199	0.08284	1.873	3.198	63	5	4	0.04762	0	96	7	88	7	PDB 9b0m:A	PDB 4p2s:D
666	0.2197	0.5577	2.723	3.312	56	6	7	0.01786	0	96	7	67	6	PDB 9b0m:A	PDB 1a0o:H
667	0.2196	0.07311	1.829	3.569	65	5	6	0.09231	0	96	7	83	6	PDB 9b0m:A	PDB 3bkt:A
668	0.2194	0.1289	2.008	3.521	66	6	4	0.07576	0	96	7	87	7	PDB 9b0m:A	PDB 4rbu:I
669	0.2194	0.2386	2.247	3.710	70	5	4	0.05714	0	96	7	92	7	PDB 9b0m:A	PDB 8b11:E
670	0.2193	0.08575	1.888	3.206	63	5	4	0.04762	0	96	7	88	7	PDB 9b0m:A	PDB 4p2s:A
671	0.2193	0.2638	2.292	3.510	67	6	5	0.08955	0	96	7	90	7	PDB 9b0m:A	PDB 7mmx:C
672	0.2192	0.1996	2.172	3.173	63	5	4	0.06349	0	96	7	89	7	PDB 9b0m:A	PDB 4rbu:D
673	0.2192	0.07002	2.446	3.396	65	5	4	0.04615	0	96	7	88	7	PDB 9b0m:A	PDB 7mmx:B

674	0.2192	0.3358	2.326	3.348	67	5	5	0.01493	0	96	7	95	5	PDB 9b0m:A	PDB 6han:C
675	0.2192	0.02865	1.636	3.941	69	6	7	0.1304	0	96	7	83	6	PDB 9b0m:A	PDB 2y3y:B
676	0.219	1.356	3.602	3.763	69	5	7	0.04348	0	96	7	88	7	PDB 9b0m:A	PDB 6lpi:G
677	0.2189	0.3056	2.366	3.640	68	6	6	0.07353	0	96	7	89	6	PDB 9b0m:A	PDB 7mpv:E
678	0.2188	0.1825	2.127	3.552	59	6	6	0.0678	0	96	7	69	6	PDB 9b0m:A	PDB 1a0o:F
679	0.2187	0.1207	1.993	3.473	65	6	4	0.09231	0	96	7	86	7	PDB 9b0m:A	PDB 4p7v:B
680	0.2187	0.07622	2.459	3.428	66	5	4	0.04545	0	96	7	90	6	PDB 9b0m:A	PDB 3mpw:E
681	0.2186	0.05513	2.097	3.243	64	5	6	0.01562	0	96	7	90	7	PDB 9b0m:A	PDB 1w2i:A
682	0.2185	0.1098	2.566	3.217	63	5	4	0.06349	0	96	7	88	7	PDB 9b0m:A	PDB 4qig:C
683	0.2185	0.2367	2.247	3.521	67	6	5	0.1194	0	96	7	90	7	PDB 9b0m:A	PDB 5d6v:A
684	0.2184	0.07931	1.873	3.373	65	5	4	0.07692	0	96	7	89	7	PDB 9b0m:A	PDB 4rbu:G
685	0.2184	0.1727	2.112	3.591	67	6	5	0.08955	0	96	7	88	6	PDB 9b0m:A	PDB 7mpv:D
686	0.2184	0.595	2.768	3.669	69	6	6	0.05797	0	96	7	91	6	PDB 9b0m:A	PDB 3ofg:A
687	0.2184	0.3783	1.993	3.557	67	5	5	0.0597	0	96	7	89	6	PDB 9b0m:A	PDB 4c3l:A
688	0.2184	0.3445	1.948	3.884	73	5	6	0.06849	0	96	7	95	6	PDB 9b0m:A	PDB 2eg2:A
689	0.2184	0.1727	2.112	3.811	71	5	5	0.05634	0	96	7	92	7	PDB 9b0m:A	PDB 8b11:C
690	0.2183	0.01878	1.903	3.153	63	5	6	0	0	96	7	90	7	PDB 9b0m:A	PDB 1v3z:A
691	0.2183	0.1435	2.052	3.242	65	5	4	0.06154	0	96	7	93	7	PDB 9b0m:A	PDB 4ox6:B
692	0.2179	0.2582	2.277	3.530	67	5	5	0.07463	0	96	7	90	7	PDB 9b0m:A	PDB 3mpw:L
693	0.2179	0.8598	2.634	3.752	63	5	4	0.09524	0	96	7	74	6	PDB 9b0m:A	PDB 5a6p:B
694	0.2178	0.1669	2.097	3.599	67	6	5	0.08955	0	96	7	88	6	PDB 9b0m:A	PDB 7mpv:B
695	0.2177	0.03536	1.68	3.872	68	6	7	0.08824	0	96	7	83	6	PDB 9b0m:A	PDB 2y3y:D
696	0.2176	0.2311	2.232	3.534	67	5	5	0.07463	0	96	7	90	7	PDB 9b0m:A	PDB 3i6p:A
697	0.2176	0.2524	2.277	3.534	67	6	6	0.1194	0	96	7	90	7	PDB 9b0m:A	PDB 5l38:C
698	0.2176	0.05239	2.366	3.257	64	5	4	0.09375	0	96	7	90	7	PDB 9b0m:A	PDB 3i6p:C
699	0.2176	0.1647	2.097	3.418	65	6	4	0.09231	0	96	7	88	7	PDB 9b0m:A	PDB 4qig:D
700	0.2176	0.1917	2.157	3.534	67	5	5	0.07463	0	96	7	90	7	PDB 9b0m:A	PDB 3i6p:D
701	0.2175	0.1805	1.784	4.344	82	5	8	0.1341	0	96	7	104	7	PDB 9b0m:A	PDB 7w6d:A
702	0.2173	0.07966	1.873	3.683	69	6	5	0.1014	0	96	7	91	6	PDB 9b0m:A	PDB 4qif:A
703	0.2172	0.6373	2.752	3.390	65	5	5	0.1077	0	96	7	89	5	PDB 9b0m:A	PDB 2lu1:A
704	0.2171	0.2047	2.187	3.742	70	5	4	0.05714	0	96	7	92	7	PDB 9b0m:A	PDB 8b12:A
705	0.217	0.1791	2.127	3.830	71	5	5	0.05634	0	96	7	92	7	PDB 9b0m:A	PDB 8b12:C
706	0.2168	0.756	2.96	3.745	70	6	6	0.04286	0	96	7	92	7	PDB 9b0m:A	PDB 5ypw:C
707	0.2168	0.2512	2.262	3.725	69	6	5	0.1014	0	96	7	90	7	PDB 9b0m:A	PDB 3i6p:E
708	0.2168	0.1105	1.963	3.725	69	5	6	0.08696	0	96	7	90	7	PDB 9b0m:A	PDB 3mpw:J
709	0.2167	0.6929	2.989	3.659	69	6	6	0.04348	0	96	7	92	7	PDB 9b0m:A	PDB 5ypw:F
710	0.2167	0.1521	2.067	3.559	66	6	4	0.07576	0	96	7	87	6	PDB 9b0m:A	PDB 4rbu:B
711	0.2165	0.2465	2.262	3.729	69	6	5	0.1014	0	96	7	90	7	PDB 9b0m:A	PDB 3i6p:B
712	0.2165	0.06803	2.433	3.365	65	5	4	0.09231	0	96	7	90	7	PDB 9b0m:A	PDB 3i6p:F
713	0.2165	0.1878	2.142	3.696	69	6	7	0.1159	0	96	7	91	6	PDB 9b0m:A	PDB 4qif:H
714	0.2164	0.08633	2.499	3.401	65	5	4	0.06154	0	96	7	89	7	PDB 9b0m:A	PDB 3ngk:A
715	0.2164	0.05836	1.799	3.819	70	5	7	0.08571	0	96	7	90	7	PDB 9b0m:A	PDB 3dn9:D
716	0.2164	0.05836	1.799	3.820	70	5	7	0.08571	0	96	7	90	7	PDB 9b0m:A	PDB 3dn9:F
717	0.2163	0.02609	1.888	3.839	74	5	5	0.04054	0	96	7	100	7	PDB 9b0m:A	PDB 2ftr:B
718	0.2162	0.9241	2.709	3.473	69	5	9	0.08696	0	96	7	98	6	PDB 9b0m:A	PDB 4c3k:C
719	0.2162	0.1319	2.023	3.565	66	6	4	0.07576	0	96	7	87	7	PDB 9b0m:A	PDB 4p2s:E
720	0.216	0.139	2.646	3.180	64	5	4	0.04688	0	96	7	93	7	PDB 9b0m:A	PDB 4rbv:A
721	0.216	0.2102	2.187	3.622	61	5	6	0.09836	0	96	7	73	6	PDB 9b0m:A	PDB 7qpx:B
722	0.2159	0.03748	1.695	4.080	61	5	6	0.1148	0	96	7	63	6	PDB 9b0m:A	PDB 6u2d:C
723	0.2158	0.5031	2.753	3.793	70	6	6	0.04286	0	96	7	91	7	PDB 9b0m:A	PDB 5ypw:H
724	0.2157	0.2165	2.515	3.790	68	5	4	0.05882	0	96	7	86	7	PDB 9b0m:A	PDB 3ofh:A
725	0.2157	0.2535	2.277	3.527	67	5	4	0.1194	0	96	7	91	6	PDB 9b0m:A	PDB 4ppd:B
726	0.2156	0.1636	2.097	3.851	71	5	5	0.05634	0	96	7	92	7	PDB 9b0m:A	PDB 8b0y:F
727	0.2155	0.03925	2.312	3.286	64	5	4	0.04688	0	96	7	90	7	PDB 9b0m:A	PDB 5l38:G
728	0.2154	0.2203	2.832	3.565	67	5	6	0.07463	0	96	7	90	7	PDB 9b0m:A	PDB 3mpw:I
729	0.2153	0.1727	2.112	3.205	58	6	5	0.1379	0	96	7	76	6	PDB 9b0m:A	PDB 2n7y:A
730	0.2152	0.254	2.406	3.579	66	5	6	0.04545	0	96	7	87	6	PDB 9b0m:A	PDB 3np5:A
731	0.2152	0.03699	2.285	3.229	63	5	4	0.04762	0	96	7	89	7	PDB 9b0m:A	PDB 4p2s:H
732	0.2151	0.0177	1.562	3.385	65	5	6	0.04615	0	96	7	90	7	PDB 9b0m:A	PDB 2bjd:A
733	0.2148	1.238	3.486	3.788	69	5	6	0.04348	0	96	7	89	7	PDB 9b0m:A	PDB 6lpi:F
734	0.2146	0.8598	2.634	3.604	72	5	7	0.06944	0	96	7	103	6	PDB 9b0m:A	PDB 2xzw:I
735	0.2145	0.4514	3.2	3.218	61	5	7	0.04918	0	96	7	84	6	PDB 9b0m:A	PDB 6y53:q
736	0.2145	0.6115	2.886	3.724	69	6	6	0.05797	0	96	7	91	7	PDB 9b0m:A	PDB 5ypw:A
737	0.2144	0.215	2.202	3.593	69	5	4	0.05797	0	96	7	95	7	PDB 9b0m:A	PDB 2g13:A
738	0.2144	0.0516	1.769	3.579	67	5	5	0.08955	0	96	7	90	7	PDB 9b0m:A	PDB 3mpw:F
739	0.2142	0.1751	2.127	3.783	70	5	4	0.05714	0	96	7	92	7	PDB 9b0m:A	PDB 8b0y:C
740	0.2141	0.1215	1.993	3.595	66	6	4	0.07576	0	96	7	87	7	PDB 9b0m:A	PDB 4p2s:G
741	0.2141	0.03089	1.814	3.400	65	5	6	0.04615	0	96	7	90	7	PDB 9b0m:A	PDB 4oj1:B
742	0.2139	0.1353	2.023	3.562	66	6	4	0.07576	0	96	7	88	7	PDB 9b0m:A	PDB 4p2s:I
743	0.2138	0.3082	2.366	3.539	69	5	4	0.07246	0	96	7	97	7	PDB 9b0m:A	PDB 2i9s:A
744	0.2137	0.1197	1.993	3.600	66	6	4	0.07576	0	96	7	87	7	PDB 9b0m:A	PDB 4p2s:C
745	0.2132	0.1359	2.038	3.550	65	6	5	0.07692	0	96	7	86	7	PDB 9b0m:A	PDB 4p7v:E
746	0.213	0.03973	1.695	3.435	63	5	6	0.09524	0	96	7	84	6	PDB 9b0m:A	PDB 1q5y:A
747	0.213	0.103	1.948	4.084	76	5	5	0.05263	0	96	7	99	6	PDB 9b0m:A	PDB 1mwq:B
748	0.2129	0.04336	2.326	3.228	63	5	4	0.04762	0	96	7	90	7	PDB 9b0m:A	PDB 4qig:A
749	0.2128	0.2402	2.247	3.430	69	5	7	0.08696	0	96	7	101	7	PDB 9b0m:A	PDB 6zol:U
750	0.2127	0.006666	1.68	4.463	83	5	5	0.09639	0	96	7	105	7	PDB 9b0m:A	PDB 5y02:H
751	0.2126	0.05403	1.754	3.327	64	5	6	0.04688	0	96	7	90	7	PDB 9b0m:A	PDB 2bjd:B
752	0.2126	0.8425	3.063	3.730	68	6	6	0.04412	0	96	7	89	7	PDB 9b0m:A	PDB 6lpi:E
753	0.2124	0.04461	1.74	4.155	69	5	6	0.1014	0	96	7	80	6	PDB 9b0m:A	PDB 2nzc:C
754	0.2122	0.12	1.651	4.470	83	5	5	0.09639	0	96	7	105	7	PDB 9b0m:A	PDB 5xzt:E
755	0.2121	0.3352	2.664	3.715	67	5	6	0.04478	0	96	7	87	7	PDB 9b0m:A	PDB 3ng8:B
756	0.2119	0.1353	2.023	3.557	66	6	4	0.07576	0	96	7	89	7	PDB 9b0m:A	PDB 4rbu:F
757	0.2119	0.4279	2.649	3.904	71	6	6	0.04225	0	96	7	92	7	PDB 9b0m:A	PDB 5ypp:C
758	0.2119	0.272	2.306	3.796	78	5	7	0.03846	0	96	7	115	6	PDB 9b0m:A	PDB 3bf4:A
759	0.2118	0.1478	2.082	4.090	77	5	5	0.09091	0	96	7	102	6	PDB 9b0m:A	PDB 3ueb:A
760	0.2116	0.0292	1.651	4.253	77	6	4	0.05195	0	96	7	97	6	PDB 9b0m:A	PDB 3mgj:A
761	0.2114	0.2762	1.829	3.691	67	5	5	0.08955	0	96	7	88	6	PDB 9b0m:A	PDB 2ko1:B
762	0.2114	0.244	2.872	3.606	63	5	6	0.09524	0	96	7	80	6	PDB 9b0m:A	PDB 1jww:A
763	0.2114	0.244	2.872	3.606	63	5	6	0.09524	0	96	7	80	6	PDB 9b0m:A	PDB 2voy:A
764	0.2113	0.1353	2.023	3.566	66	6	4	0.07576	0	96	7	89	7	PDB 9b0m:A	PDB 4p2s:B
765	0.2112	0.1678	1.607	3.985	58	5	5	0.1379	0	96	7	60	6	PDB 9b0m:A	PDB 4zoq:B
766	0.2112	0.1211	1.993	3.672	69	6	5	0.1014	0	96	7	94	6	PDB 9b0m:A	PDB 4rbv:B
767	0.211	0.009947	2.057	3.418	64	5	5	0.04688	0	96	7	88	7	PDB 9b0m:A	PDB 4qie:F
768	0.2108	0.6885	2.426	3.556	68	5	8	0.1176	0	96	7	95	6	PDB 9b0m:A	PDB 3t9z:C
769	0.2108	0.1051	1.933	3.503	63	5	5	0.07937	0	96	7	83	6	PDB 9b0m:A	PDB 1q5y:B
770	0.2106	0.05331	2.379	3.261	63	5	5	0.04762	0	96	7	90	7	PDB 9b0m:A	PDB 4qif:C

771	0.2105	0.1323	2.023	3.510	66	6	5	0.09091	0	96	7	91	6	PDB 9b0m:A	PDB 7mpv:F
772	0.2104	0.03822	2.299	3.297	63	5	5	0.04762	0	96	7	89	7	PDB 9b0m:A	PDB 4p2s:F
773	0.2102	0.1353	2.023	3.774	70	5	5	0.08571	0	96	7	94	7	PDB 9b0m:A	PDB 3cim:A
774	0.2102	0.6217	2.798	3.697	68	6	6	0.04412	0	96	7	91	7	PDB 9b0m:A	PDB 5ypw:E
775	0.21	0.3296	1.918	4.054	72	5	6	0.08333	0	96	7	91	6	PDB 9b0m:A	PDB 1vfj:C
776	0.21	0.2154	1.814	4.287	81	5	7	0.1235	0	96	7	107	7	PDB 9b0m:A	PDB 5xzt:H
777	0.2098	0.02639	2.038	3.507	68	5	4	0.05882	0	96	7	97	7	PDB 9b0m:A	PDB 2dcl:C
778	0.2097	0.2369	1.74	3.885	64	5	6	0.07812	0	96	7	76	6	PDB 9b0m:A	PDB 2e1a:D
779	0.2097	0.05657	1.784	3.615	64	6	5	0.07812	0	96	7	83	6	PDB 9b0m:A	PDB 3bku:C
780	0.2096	0.08716	1.903	3.829	69	5	7	0.08696	0	96	7	90	7	PDB 9b0m:A	PDB 4liw:B
781	0.2091	0.609	2.321	3.523	67	5	8	0.1194	0	96	7	94	6	PDB 9b0m:A	PDB 3t9z:F
782	0.2089	0.1536	1.976	3.542	60	5	3	0.01667	0	96	7	75	6	PDB 9b0m:A	PDB 4lij:A
783	0.2088	0.1525	2.067	4.032	75	5	4	0.06667	0	96	7	100	6	PDB 9b0m:A	PDB 1mwq:A
784	0.2088	0.01744	1.71	3.442	65	5	5	0.04615	0	96	7	91	7	PDB 9b0m:A	PDB 4ojg:B
785	0.2085	0.1878	2.142	3.622	68	5	4	0.04412	0	96	7	94	7	PDB 9b0m:A	PDB 5djb:E
786	0.2084	0.1369	2.038	3.507	66	6	5	0.09091	0	96	7	92	6	PDB 9b0m:A	PDB 7mpv:G
787	0.2082	0.06001	1.784	3.824	66	5	6	0.07576	0	96	7	83	6	PDB 9b0m:A	PDB 2y3y:A
788	0.2081	0.4137	2.515	3.261	64	5	6	0.09375	0	96	7	94	6	PDB 9b0m:A	PDB 4cny:A
789	0.208	0.006822	1.431	3.326	64	5	4	0.04688	0	96	7	92	7	PDB 9b0m:A	PDB 4ppd:A
790	0.208	0.2315	2.232	3.629	68	5	4	0.04412	0	96	7	94	7	PDB 9b0m:A	PDB 5djb:F
791	0.208	0.6812	3.161	3.059	56	5	4	0.07143	0	96	7	77	6	PDB 9b0m:A	PDB 8ab1:D
792	0.2079	0.5124	2.187	3.639	67	5	8	0.08955	0	96	7	91	6	PDB 9b0m:A	PDB 1v3s:C
793	0.2078	0.1164	1.978	3.822	69	6	5	0.07246	0	96	7	91	7	PDB 9b0m:A	PDB 3bn4:B
794	0.2077	0.8546	2.62	3.330	57	5	5	0.08772	0	96	7	73	6	PDB 9b0m:A	PDB 1mwy:A
795	0.2075	0.01529	1.799	4.416	81	5	5	0.1111	0	96	7	104	7	PDB 9b0m:A	PDB 5xzt:I
796	0.2075	6.438e-05	1.842	3.591	71	5	8	0.09859	0	96	7	104	7	PDB 9b0m:A	PDB 7m4u:f
797	0.2075	0.3	2.964	3.624	70	5	7	0.08571	0	96	7	100	7	PDB 9b0m:A	PDB 7k5i:U
798	0.2073	0.01599	1.814	4.309	80	5	6	0.1	0	96	7	105	7	PDB 9b0m:A	PDB 5y02:I
799	0.2071	0.2	2.172	3.552	67	6	5	0.0597	0	96	7	94	7	PDB 9b0m:A	PDB 5djb:G
800	0.2071	1.224e-08	1.601	3.379	63	5	6	0.04762	0	96	7	88	7	PDB 9b0m:A	PDB 4hi2:D
801	0.207	1.018	3.253	3.847	68	5	6	0.02941	0	96	7	88	7	PDB 9b0m:A	PDB 6lpi:H
802	0.2069	0.1467	2.339	3.253	68	5	7	0.05882	0	96	7	107	7	PDB 9b0m:A	PDB 3ahp:C
803	0.2069	0.003469	1.607	3.938	75	5	7	0.09333	0	96	7	104	7	PDB 9b0m:A	PDB 7w6e:A
804	0.2069	0.1855	1.754	3.516	69	5	5	0.07246	0	96	7	101	7	PDB 9b0m:A	PDB 2od4:B
805	0.2068	0.2669	1.814	3.759	67	5	5	0.1045	0	96	7	88	6	PDB 9b0m:A	PDB 2ko1:A
806	0.2068	0.1525	2.067	3.288	58	5	6	0.06897	0	96	7	77	6	PDB 9b0m:A	PDB 6r8k:B
807	0.2066	0.7664	2.911	3.278	59	5	4	0.0678	0	96	7	80	6	PDB 9b0m:A	PDB 8a9x:A
808	0.2066	0.5821	2.857	3.784	68	6	6	0.04412	0	96	7	90	7	PDB 9b0m:A	PDB 5ypw:D
809	0.2063	0.08968	1.903	3.478	65	6	4	0.07692	0	96	7	91	7	PDB 9b0m:A	PDB 4qif:F
810	0.2063	0.4284	2.067	3.921	71	5	6	0.05634	0	96	7	94	6	PDB 9b0m:A	PDB 1ul3:B
811	0.2061	0.1965	1.666	4.065	67	5	5	0.04478	0	96	7	80	6	PDB 9b0m:A	PDB 1q5y:D
812	0.206	0.609	2.886	3.725	68	6	6	0.04412	0	96	7	92	7	PDB 9b0m:A	PDB 5ypy:A
813	0.2054	0.116	1.978	3.823	69	5	5	0.07246	0	96	7	92	7	PDB 9b0m:A	PDB 3bn4:F
814	0.2054	0.1804	2.247	3.805	71	6	7	0.07042	0	96	7	98	7	PDB 9b0m:A	PDB 2lvw:B
815	0.2053	0.1804	2.247	3.806	71	6	7	0.07042	0	96	7	98	7	PDB 9b0m:A	PDB 2lvw:A
816	0.2051	0.07601	1.859	3.327	60	5	5	0.1	0	96	7	82	6	PDB 9b0m:A	PDB 2nzc:D
817	0.205	0.1669	2.097	3.739	68	5	5	0.08824	0	96	7	92	7	PDB 9b0m:A	PDB 3bn4:C
818	0.205	0.1917	2.157	3.830	69	5	5	0.07246	0	96	7	92	7	PDB 9b0m:A	PDB 3bn4:E
819	0.205	0.1669	2.097	3.740	68	5	5	0.08824	0	96	7	92	7	PDB 9b0m:A	PDB 3bn4:A
820	0.2047	0.2676	2.292	3.895	73	5	7	0.08219	0	96	7	101	7	PDB 9b0m:A	PDB 6zoj:U
821	0.2046	0.1021	1.445	3.824	63	5	6	0.07937	0	96	7	77	6	PDB 9b0m:A	PDB 2z4p:B
822	0.2041	0.2465	2.262	3.287	64	5	5	0.07812	0	96	7	95	7	PDB 9b0m:A	PDB 3toq:A
823	0.2041	0.1573	2.097	3.403	56	5	8	0.01786	0	96	7	70	5	PDB 9b0m:A	PDB 1a0o:B
824	0.2038	0.1306	2.339	3.680	63	5	5	0.06349	0	96	7	81	6	PDB 9b0m:A	PDB 5n7l:A
825	0.2037	2.771e-11	1.416	3.610	66	5	7	0.0303	0	96	7	91	7	PDB 9b0m:A	PDB 6krb:F
826	0.2036	0.3596	1.963	4.009	70	5	7	0.08571	0	96	7	90	6	PDB 9b0m:A	PDB 1v3r:C
827	0.2035	0.524	2.202	3.454	65	5	5	0.1231	0	96	7	93	6	PDB 9b0m:A	PDB 7egl:B
828	0.203	0.01914	1.71	3.769	68	5	7	0.05882	0	96	7	92	7	PDB 9b0m:A	PDB 5ypp:E
829	0.203	0.1014	1.963	4.162	77	5	4	0.07792	0	96	7	104	6	PDB 9b0m:A	PDB 3ueb:E
830	0.203	0.07661	2.191	3.331	59	5	4	0.05085	0	96	7	80	6	PDB 9b0m:A	PDB 3dnj:A
831	0.2029	0.6917	3.174	2.469	56	5	4	0.1071	0	96	7	96	7	PDB 9b0m:A	PDB 2ebb:A
832	0.2028	0.2156	2.419	3.373	69	5	4	0.1014	0	96	7	108	6	PDB 9b0m:A	PDB 1kkg:A
833	0.2027	0.04215	1.978	4.430	81	5	6	0.09877	0	96	7	106	7	PDB 9b0m:A	PDB 1tr0:Y
834	0.2026	0.1761	2.127	3.619	67	5	5	0.04478	0	96	7	94	7	PDB 9b0m:A	PDB 5djb:H
835	0.2025	1.1	3.278	3.441	60	5	4	0.1167	0	96	7	80	6	PDB 9b0m:A	PDB 1xs3:A
836	0.2024	0.04544	1.993	4.435	81	5	6	0.09877	0	96	7	106	7	PDB 9b0m:A	PDB 1tr0:I
837	0.2022	0.00226	1.301	4.137	70	5	6	0.04286	0	96	7	87	6	PDB 9b0m:A	PDB 1rwu:A
838	0.202	0.02875	1.518	3.825	71	5	4	0.01408	0	96	7	99	7	PDB 9b0m:A	PDB 1lxn:A
839	0.202	0.4897	2.157	3.670	66	5	8	0.09091	0	96	7	90	6	PDB 9b0m:A	PDB 1v9o:C
840	0.2018	0.04215	1.978	4.444	81	5	6	0.09877	0	96	7	106	7	PDB 9b0m:A	PDB 1tr0:S
841	0.2018	0.1584	2.686	2.906	52	5	5	0.1538	0	96	7	72	6	PDB 9b0m:A	PDB 3fry:B
842	0.2016	0.2239	2.217	3.507	67	5	5	0.07463	0	96	7	98	7	PDB 9b0m:A	PDB 3mpw:G
843	0.2016	0.8997	2.679	3.510	66	5	8	0.06061	0	96	7	95	6	PDB 9b0m:A	PDB 2eg1:A
844	0.2015	0.0421	1.725	3.946	65	5	5	0.1231	0	96	7	80	6	PDB 9b0m:A	PDB 2nzc:A
845	0.2013	0.01099	1.754	4.084	72	5	6	0.08333	0	96	7	94	7	PDB 9b0m:A	PDB 5b0b:C
846	0.2012	0.0614	1.799	3.777	62	5	5	0.09677	0	96	7	77	6	PDB 9b0m:A	PDB 1itp:A
847	0.2011	0.5401	2.99	3.160	56	5	4	0.07143	0	96	7	77	6	PDB 9b0m:A	PDB 8ab1:B
848	0.201	0.03901	1.963	4.458	81	5	6	0.09877	0	96	7	106	7	PDB 9b0m:A	PDB 1tr0:N
849	0.2009	0.1405	1.636	3.744	67	5	6	0.1194	0	96	7	91	7	PDB 9b0m:A	PDB 5ypy:D
850	0.2008	0.1075	1.548	3.928	69	5	6	0.1014	0	96	7	91	7	PDB 9b0m:A	PDB 5ypp:F
851	0.2008	0.09298	1.344	4.081	73	5	7	0.05479	0	96	7	97	6	PDB 9b0m:A	PDB 4zos:B
852	0.2007	0.1153	1.621	4.573	82	5	6	0.09756	0	96	7	105	7	PDB 9b0m:A	PDB 5y02:D
853	0.2005	0.1861	2.142	3.618	67	5	5	0.04478	0	96	7	95	7	PDB 9b0m:A	PDB 5djb:C
854	0.2005	0.03973	1.963	4.466	81	5	6	0.09877	0	96	7	106	7	PDB 9b0m:A	PDB 1tr0:B
855	0.2005	0.01247	2.097	3.310	63	5	4	0.07937	0	96	7	93	7	PDB 9b0m:A	PDB 7mgp:A
856	0.2004	0.2184	2.202	3.762	70	6	5	0.08571	0	96	7	99	8	PDB 9b0m:A	PDB 3ssr:B
857	0.2003	0.03509	1.948	4.469	81	5	6	0.09877	0	96	7	106	7	PDB 9b0m:A	PDB 1tr0:U
858	0.2001	0.6111	2.783	3.896	67	5	5	0.1194	0	96	7	87	6	PDB 9b0m:A	PDB 7mp7:A
859	0.2001	0.03672	1.948	4.473	81	5	6	0.09877	0	96	7	106	7	PDB 9b0m:A	PDB 1si9:A
860	0.1996	0.2025	1.814	4.450	81	5	7	0.09877	0	96	7	107	7	PDB 9b0m:A	PDB 5xzt:C
861	0.1994	0.2117	1.784	3.949	69	5	6	0.1014	0	96	7	91	7	PDB 9b0m:A	PDB 5ypw:G
862	0.1994	0.002956	1.882	3.902	65	5	5	0.06154	0	96	7	82	6	PDB 9b0m:A	PDB 2mew:A
863	0.1993	0.01487	1.799	4.498	80	5	6	0.0875	0	96	7	103	7	PDB 9b0m:A	PDB 5y02:K
864	0.1992	0.7754	2.53	3.603	68	5	8	0.1176	0	96	7	99	6	PDB 9b0m:A	PDB 3ta0:C
865	0.1991	0.5481	2.709	3.948	67	5	6	0.08955	0	96	7	86	7	PDB 9b0m:A	PDB 1u0s:A
866	0.199	0.1458	2.339	3.281	67	5	8	0.02985	0	96	7	107	7	PDB 9b0m:A	PDB 3ahp:F
867	0.1989	0.09291	2.232	2.553	51	5	4	0.07843	0	96	7	79	6	PDB 9b0m:A	PDB 4low:B

868	0.1988	0.04571	1.74	3.714	61	5	6	0.09836	0	96	7	77	7	PDB 9b0m:A	PDB 2n2u:A
869	0.1987	0.009809	1.74	4.525	81	5	6	0.08642	0	96	7	105	7	PDB 9b0m:A	PDB 5xzt:D
870	0.1985	0.01644	1.814	4.578	82	5	5	0.09756	0	96	7	106	7	PDB 9b0m:A	PDB 1tr0:K
871	0.1983	0.1273	1.518	4.108	67	5	5	0.0597	0	96	7	82	6	PDB 9b0m:A	PDB 3bku:B
872	0.1982	0.2525	1.769	3.661	66	5	6	0.1061	0	96	7	92	7	PDB 9b0m:A	PDB 5yum:A
873	0.1981	0.002158	1.301	3.729	63	5	7	0.09524	0	96	7	82	6	PDB 9b0m:A	PDB 1q5y:C
874	0.198	0.09745	2.259	3.717	64	5	3	0.07812	0	96	7	85	6	PDB 9b0m:A	PDB 3o2o:F
875	0.1979	0.09745	2.259	3.718	64	5	3	0.07812	0	96	7	85	6	PDB 9b0m:A	PDB 3o2o:H
876	0.1979	0.09904	2.259	3.718	64	5	3	0.07812	0	96	7	85	6	PDB 9b0m:A	PDB 3o2o:C
877	0.1979	0.2124	1.695	3.510	64	5	6	0.0625	0	96	7	91	6	PDB 9b0m:A	PDB 1b64:A
878	0.1979	0.1657	1.607	4.042	65	5	5	0.01538	0	96	7	79	6	PDB 9b0m:A	PDB 3bkt:D
879	0.1978	0.09677	2.259	3.721	64	5	3	0.07812	0	96	7	85	6	PDB 9b0m:A	PDB 3o2o:D
880	0.1978	0.09545	2.245	3.721	64	5	3	0.07812	0	96	7	85	6	PDB 9b0m:A	PDB 3o2o:B
881	0.1975	0.002881	1.695	3.704	66	5	6	0.04545	0	96	7	91	7	PDB 9b0m:A	PDB 6krb:C
882	0.1975	0.007037	1.695	4.496	80	5	5	0.1	0	96	7	104	7	PDB 9b0m:A	PDB 5y02:L
883	0.1974	0.09322	2.245	3.726	64	5	3	0.07812	0	96	7	85	6	PDB 9b0m:A	PDB 3o2o:G
884	0.1974	0.9193	3.151	4.015	69	6	4	0.1014	0	96	7	90	6	PDB 9b0m:A	PDB 6wue:A
885	0.1974	0.001367	1.621	3.798	67	5	6	0.0597	0	96	7	91	7	PDB 9b0m:A	PDB 6krb:L
886	0.1972	0.1826	2.142	3.240	65	6	4	0.06154	0	96	7	103	8	PDB 9b0m:A	PDB 4ox6:A
887	0.1971	0.1856	2.142	3.634	69	6	4	0.05797	0	96	7	102	8	PDB 9b0m:A	PDB 2a10:E
888	0.1969	0.01056	1.475	4.139	65	5	5	0.1538	0	96	7	77	6	PDB 9b0m:A	PDB 2g9o:A
889	0.1967	0.08179	2.205	3.738	64	5	3	0.07812	0	96	7	85	6	PDB 9b0m:A	PDB 3o2o:E
890	0.1966	0.07638	2.178	3.039	56	5	5	0.125	0	96	7	82	6	PDB 9b0m:A	PDB 2w7v:A
891	0.1966	0.07638	2.178	3.039	56	5	5	0.125	0	96	7	82	6	PDB 9b0m:A	PDB 2w7v:B
892	0.1965	0.0932	2.245	3.741	64	5	3	0.07812	0	96	7	85	6	PDB 9b0m:A	PDB 3o2o:A
893	0.1961	0.7014	2.426	3.625	62	5	6	0.1452	0	96	7	83	6	PDB 9b0m:A	PDB 2zbc:B
894	0.1956	0.2424	2.247	3.843	69	5	5	0.04348	0	96	7	96	7	PDB 9b0m:A	PDB 6cbu:A
895	0.1954	1.149	3.486	2.597	57	5	3	0.08772	0	96	7	99	6	PDB 9b0m:A	PDB 1dch:D
896	0.1952	0.01188	1.504	4.007	65	6	6	0.1385	0	96	7	81	6	PDB 9b0m:A	PDB 2y3y:C
897	0.195	0.6634	2.381	3.816	63	5	5	0.1429	0	96	7	81	6	PDB 9b0m:A	PDB 2zbc:D
898	0.1948	0.04618	2.366	3.760	69	5	3	0.1014	0	96	7	99	6	PDB 9b0m:A	PDB 3ueb:D
899	0.1947	0.03973	1.963	4.560	81	5	5	0.07407	0	96	7	106	7	PDB 9b0m:A	PDB 1tr0:F
900	0.1947	0.1929	1.769	4.562	81	5	5	0.1235	0	96	7	106	7	PDB 9b0m:A	PDB 1si9:B
901	0.1947	0.1526	2.217	3.612	69	6	4	0.05797	0	96	7	104	8	PDB 9b0m:A	PDB 2a10:A
902	0.1946	0.164	1.592	3.819	61	5	7	0.08197	0	96	7	76	6	PDB 9b0m:A	PDB 2e1a:B
903	0.1934	0.6403	2.938	2.628	57	5	4	0.1228	0	96	7	99	6	PDB 9b0m:A	PDB 1dch:H
904	0.193	0.04892	1.621	3.790	69	5	3	0.01449	0	96	7	99	7	PDB 9b0m:A	PDB 1lxn:D
905	0.1928	0.1983	2.157	3.280	65	5	7	0.04615	0	96	7	104	7	PDB 9b0m:A	PDB 7qiy:L
906	0.1927	1.828e-07	1.721	3.853	70	5	6	0.07143	0	96	7	100	7	PDB 9b0m:A	PDB 7syo:V
907	0.1924	0.04215	1.978	4.518	80	5	5	0.1	0	96	7	106	7	PDB 9b0m:A	PDB 1tr0:O
908	0.1924	0.0445	1.993	4.519	80	5	5	0.1	0	96	7	106	7	PDB 9b0m:A	PDB 1tr0:V
909	0.1923	0.997	3.33	2.538	56	5	4	0.1071	0	96	7	99	6	PDB 9b0m:A	PDB 1dco:F
910	0.1923	0.6766	2.396	3.775	59	5	6	0.01695	0	96	7	73	6	PDB 9b0m:A	PDB 2k1r:A
911	0.1919	0.6748	2.977	2.545	56	5	3	0.08929	0	96	7	99	6	PDB 9b0m:A	PDB 1f93:C
912	0.1915	0.02285	1.495	4.127	64	5	3	0.07812	0	96	7	77	6	PDB 9b0m:A	PDB 4lij:C
913	0.1915	0.02144	1.592	3.969	64	5	5	0.125	0	96	7	81	6	PDB 9b0m:A	PDB 2nzc:B
914	0.1913	0.6554	2.951	2.766	58	5	4	0.1034	0	96	7	99	6	PDB 9b0m:A	PDB 1dco:E
915	0.1913	0.9854	3.317	2.555	56	5	4	0.1071	0	96	7	99	6	PDB 9b0m:A	PDB 1dco:H
916	0.1913	0.9188	3.252	2.555	56	5	3	0.08929	0	96	7	99	6	PDB 9b0m:A	PDB 1dco:B
917	0.1913	0.609	2.321	3.528	70	5	9	0.1143	0	96	7	112	6	PDB 9b0m:A	PDB 3ta1:F
918	0.1912	0.002956	1.882	3.534	55	5	5	0.05455	0	96	7	69	6	PDB 9b0m:A	PDB 2kt2:A
919	0.1911	0.01893	1.577	3.754	64	5	5	0.1406	0	96	7	87	6	PDB 9b0m:A	PDB 3ofg:B
920	0.1911	1.043	3.226	2.558	56	5	4	0.08929	0	96	7	99	6	PDB 9b0m:A	PDB 1dco:D
921	0.191	0.1642	2.084	3.758	61	5	7	0.1148	0	96	7	79	6	PDB 9b0m:A	PDB 1x0f:A
922	0.1909	0.4128	3.148	3.550	66	5	8	0.1061	0	96	7	99	7	PDB 9b0m:A	PDB 5ovo:B
923	0.1907	0.6388	2.938	2.670	57	5	4	0.08772	0	96	7	99	6	PDB 9b0m:A	PDB 1dcp:E
924	0.1907	1.032	3.213	2.563	56	5	4	0.08929	0	96	7	99	6	PDB 9b0m:A	PDB 1dcp:D
925	0.1906	0.9901	3.174	2.689	66	5	5	0.06061	0	96	7	132	5	PDB 9b0m:A	PDB 3jd5:C
926	0.1905	0.9254	3.252	2.567	56	5	3	0.08929	0	96	7	99	6	PDB 9b0m:A	PDB 1dco:G
927	0.1904	0.7099	3.017	2.569	56	5	3	0.08929	0	96	7	99	6	PDB 9b0m:A	PDB 1dcp:G
928	0.1903	0.9685	3.304	2.569	56	5	4	0.1071	0	96	7	99	6	PDB 9b0m:A	PDB 1dcp:B
929	0.1903	0.797	3.122	2.782	58	5	4	0.1207	0	96	7	99	6	PDB 9b0m:A	PDB 1dcp:A
930	0.1902	0.002849	1.285	3.575	54	5	5	0.1296	0	96	7	66	5	PDB 9b0m:A	PDB 6u2d:A
931	0.1898	0.5211	2.187	3.800	62	5	5	0.1452	0	96	7	81	6	PDB 9b0m:A	PDB 2zbc:C
932	0.1896	0.8613	3.187	2.471	55	5	4	0.1091	0	96	7	99	6	PDB 9b0m:A	PDB 1dcp:F
933	0.1895	0.9201	3.252	2.583	56	5	3	0.1071	0	96	7	99	6	PDB 9b0m:A	PDB 1dcp:H
934	0.1894	0.2213	2.515	3.757	63	5	4	0.06349	0	96	7	85	7	PDB 9b0m:A	PDB 3off:A
935	0.1894	0.06584	1.68	3.758	68	5	3	0.01471	0	96	7	99	7	PDB 9b0m:A	PDB 1lxn:B
936	0.1892	0.5962	2.885	3.102	61	5	3	0.09836	0	96	7	99	6	PDB 9b0m:A	PDB 1dch:E
937	0.1892	0.02687	2.262	3.102	61	6	6	0.04918	0	96	7	99	8	PDB 9b0m:A	PDB 3m05:C
938	0.189	0.2281	2.54	2.640	52	5	4	0.07692	0	96	7	84	6	PDB 9b0m:A	PDB 4low:A
939	0.189	0.3991	2.023	4.071	63	5	6	0.1905	0	96	7	77	6	PDB 9b0m:A	PDB 6gc5:D
940	0.1889	1.908	4.086	2.552	62	5	7	0.06452	0	96	7	123	5	PDB 9b0m:A	PDB 6nf8:C
941	0.1888	0.02735	2.232	3.505	60	5	6	0.05	0	96	7	84	6	PDB 9b0m:A	PDB 2n2t:A
942	0.1884	0.2041	1.695	3.595	59	5	5	0.01695	0	96	7	79	6	PDB 9b0m:A	PDB 1kvi:A
943	0.1884	0.04825	1.258	3.961	61	5	6	0.04918	0	96	7	75	6	PDB 9b0m:A	PDB 2z4p:D
944	0.1883	0.7415	2.885	2.709	57	5	5	0.1228	0	96	7	99	6	PDB 9b0m:A	PDB 2v6u:B
945	0.1883	0.03509	1.948	3.803	70	5	6	0.1	0	96	7	104	7	PDB 9b0m:A	PDB 5xzq:K
946	0.1881	0.006206	1.68	3.835	71	5	7	0.1268	0	96	7	106	7	PDB 9b0m:A	PDB 1tr0:R
947	0.1879	0.1561	2.686	3.548	61	5	8	0.08197	0	96	7	86	6	PDB 9b0m:A	PDB 6xh1:A
948	0.1878	0.2891	2.321	3.249	61	6	8	0.09836	0	96	7	95	6	PDB 9b0m:A	PDB 1ul3:D
949	0.1876	0.1262	1.621	3.902	71	5	6	0.09859	0	96	7	104	7	PDB 9b0m:A	PDB 5xzt:F
950	0.1874	0.005095	1.651	3.845	71	5	7	0.1268	0	96	7	106	7	PDB 9b0m:A	PDB 1tr0:D
951	0.1873	0.006206	1.68	3.847	71	5	7	0.1268	0	96	7	106	7	PDB 9b0m:A	PDB 1tr0:E
952	0.1873	0.006054	1.666	3.847	71	5	7	0.1268	0	96	7	106	7	PDB 9b0m:A	PDB 1tr0:G
953	0.1873	0.2812	2.938	3.457	60	5	9	0.08333	0	96	7	86	6	PDB 9b0m:A	PDB 5ddo:G
954	0.1872	0.0923	2.277	3.679	71	6	8	0.09859	0	96	7	112	7	PDB 9b0m:A	PDB 3mhy:B
955	0.1872	0.006054	1.666	3.849	71	5	7	0.1268	0	96	7	106	7	PDB 9b0m:A	PDB 1tr0:H
956	0.1872	0.007037	1.695	3.849	71	5	7	0.1268	0	96	7	106	7	PDB 9b0m:A	PDB 1tr0:M
957	0.1872	0.006206	1.68	3.850	71	5	7	0.1268	0	96	7	106	7	PDB 9b0m:A	PDB 1tr0:A
958	0.1872	0.6491	2.951	2.511	55	5	3	0.1091	0	96	7	99	6	PDB 9b0m:A	PDB 1dco:A
959	0.1871	0.8777	3.2	2.592	56	5	4	0.08929	0	96	7	100	6	PDB 9b0m:A	PDB 1f93:D
960	0.187	0.007971	1.71	3.939	72	5	6	0.125	0	96	7	106	7	PDB 9b0m:A	PDB 1tr0:L
961	0.187	0.007544	1.695	3.852	71	5	7	0.1268	0	96	7	106	7	PDB 9b0m:A	PDB 1tr0:P
962	0.187	0.007037	1.695	3.853	71	5	7	0.1268	0	96	7	106	7	PDB 9b0m:A	PDB 1tr0:T
963	0.187	0.007266	1.695	3.853	71	5	7	0.1268	0	96	7	106	7	PDB 9b0m:A	PDB 1tr0:C
964	0.1869	0.7664	2.911	2.626	56	5	5	0.1071	0	96	7	99	6	PDB 9b0m:A	PDB 2v6t:A

965	0.1868	0.08632	2.499	3.630	62	5	8	0.09677	0	96	7	87	6	PDB 9b0m:A	PDB 3ud4:P
966	0.1866	0.3345	1.993	3.462	62	5	6	0.1129	0	96	7	92	7	PDB 9b0m:A	PDB 5ypy:C
967	0.1865	0.5429	2.232	3.436	69	5	9	0.1159	0	96	7	115	6	PDB 9b0m:A	PDB 3ta1:D
968	0.1864	0.3309	3.017	3.636	62	5	9	0.08065	0	96	7	87	6	PDB 9b0m:A	PDB 3ud3:P
969	0.1863	2.796e-05	1.508	3.626	65	5	6	0.04615	0	96	7	96	6	PDB 9b0m:A	PDB 2diu:A
970	0.1862	0.1311	2.633	3.511	60	5	8	0.1167	0	96	7	85	6	PDB 9b0m:A	PDB 7dwh:B
971	0.186	0.6051	2.845	3.567	64	5	6	0.1094	0	96	7	95	6	PDB 9b0m:A	PDB 4kso:A
972	0.186	0.1575	2.699	3.418	58	5	8	0.08621	0	96	7	82	6	PDB 9b0m:A	PDB 3k0j:D
973	0.1859	0.7099	3.017	2.500	55	5	4	0.1273	0	96	7	100	6	PDB 9b0m:A	PDB 3hxa:A
974	0.1856	0.7755	2.924	2.616	56	5	5	0.1071	0	96	7	100	6	PDB 9b0m:A	PDB 2v6t:B
975	0.1855	0.07707	2.191	3.730	68	5	5	0.1176	0	96	7	102	7	PDB 9b0m:A	PDB 7bl4:6
976	0.1854	0.00563	1.666	3.879	71	5	7	0.1127	0	96	7	106	7	PDB 9b0m:A	PDB 1tr0:W
977	0.1854	0.06541	2.419	3.453	60	5	9	0.08333	0	96	7	87	6	PDB 9b0m:A	PDB 3ucu:P
978	0.1854	0.006612	1.68	3.880	71	5	7	0.1127	0	96	7	106	7	PDB 9b0m:A	PDB 1tr0:X
979	0.1854	0.006612	1.68	3.880	71	5	7	0.1127	0	96	7	106	7	PDB 9b0m:A	PDB 1tr0:J
980	0.1847	0.0876	2.218	3.563	57	5	4	0.07018	0	96	7	76	6	PDB 9b0m:A	PDB 1uv7:A
981	0.1846	0.001959	1.548	4.347	73	5	5	0.06849	0	96	7	97	7	PDB 9b0m:A	PDB 2ifx:B
982	0.1846	0.1851	2.292	3.599	68	6	4	0.04412	0	96	7	107	8	PDB 9b0m:A	PDB 2a10:C
983	0.1845	0.569	2.845	2.586	55	5	4	0.1091	0	96	7	98	6	PDB 9b0m:A	PDB 3hxa:C
984	0.1844	0.6748	2.977	2.665	56	5	4	0.1786	0	96	7	99	6	PDB 9b0m:A	PDB 4c45:A
985	0.1844	0.07983	2.473	3.570	61	5	8	0.09836	0	96	7	87	6	PDB 9b0m:A	PDB 3mxh:P
986	0.1843	0.05156	2.352	3.671	62	5	8	0.08065	0	96	7	87	6	PDB 9b0m:A	PDB 7dlz:B
987	0.1837	0.08851	2.499	3.582	61	5	8	0.08197	0	96	7	87	6	PDB 9b0m:A	PDB 6xh0:A
988	0.1835	0.7752	3.095	2.572	55	5	3	0.09091	0	96	7	99	6	PDB 9b0m:A	PDB 1f93:B
989	0.1834	1.171	2.989	2.922	54	5	6	0.03704	0	96	7	85	6	PDB 9b0m:A	PDB 2l48:B
990	0.1833	0.186	1.666	4.398	67	5	5	0.0597	0	96	7	81	7	PDB 9b0m:A	PDB 2lu2:A
991	0.1832	0.04469	1.993	3.887	71	5	6	0.1127	0	96	7	107	7	PDB 9b0m:A	PDB 5xzq:I
992	0.1832	0.6048	2.898	2.576	55	5	4	0.1091	0	96	7	99	6	PDB 9b0m:A	PDB 3hxa:B
993	0.1829	0.004049	1.621	3.921	71	5	7	0.1127	0	96	7	106	7	PDB 9b0m:A	PDB 1si9:C
994	0.1826	0.2283	2.845	3.497	61	5	8	0.09836	0	96	7	90	6	PDB 9b0m:A	PDB 3mur:P
995	0.1824	0.3085	2.486	2.478	54	5	3	0.1296	0	96	7	99	6	PDB 9b0m:A	PDB 1dco:C
996	0.1821	0.02001	1.201	4.586	76	5	6	0.05263	0	96	7	99	7	PDB 9b0m:A	PDB 1xbw:A
997	0.182	0.4325	2.606	3.728	66	5	6	0.1061	0	96	7	98	6	PDB 9b0m:A	PDB 2qke:C
998	0.1819	0.3129	2.99	3.408	60	5	9	0.08333	0	96	7	90	7	PDB 9b0m:A	PDB 4w92:B
999	0.1819	0.002525	1.668	3.683	61	5	5	0.08197	0	96	7	85	7	PDB 9b0m:A	PDB 1hdn:A
1000	0.1818	0.1787	2.441	3.464	56	6	4	0.07143	0	96	7	77	7	PDB 9b0m:A	PDB 4rwx:C
1001	0.1818	0.7205	3.03	2.569	55	5	4	0.1273	0	96	7	100	6	PDB 9b0m:A	PDB 3hxa:F
1002	0.1814	0.01467	2.526	3.349	58	5	4	0.1207	0	96	7	86	7	PDB 9b0m:A	PDB 3j80:j
1003	0.181	0.001592	1.829	3.114	57	5	8	0.1228	0	96	7	90	6	PDB 9b0m:A	PDB 3ns5:B
1004	0.1809	0.4337	2.673	2.503	54	5	3	0.1296	0	96	7	99	6	PDB 9b0m:A	PDB 1dcp:C
1005	0.1808	0.6944	3.003	2.585	55	5	4	0.1091	0	96	7	100	6	PDB 9b0m:A	PDB 3hxa:D
1006	0.1807	1.202	3.019	3.184	56	5	6	0.03571	0	96	7	85	6	PDB 9b0m:A	PDB 2l48:A
1007	0.1807	0.00867	1.725	3.871	70	5	6	0.1286	0	96	7	106	7	PDB 9b0m:A	PDB 5xzq:E
1008	0.1806	0.5962	2.885	2.588	55	5	3	0.1818	0	96	7	100	6	PDB 9b0m:A	PDB 1ru0:A
1009	0.1806	0.0123	1.769	3.755	69	5	7	0.1014	0	96	7	107	7	PDB 9b0m:A	PDB 5xzq:H
1010	0.1804	0.07107	1.844	3.759	57	5	5	0.05263	0	96	7	73	6	PDB 9b0m:A	PDB 3hlu:B
1011	0.1801	0.0323	1.933	3.824	70	5	6	0.1	0	96	7	108	7	PDB 9b0m:A	PDB 5xzq:J
1012	0.1798	0.04715	2.008	3.887	69	5	4	0.1159	0	96	7	103	7	PDB 9b0m:A	PDB 1q4r:A
1013	0.1797	0.116	2.593	3.512	61	5	8	0.09836	0	96	7	91	6	PDB 9b0m:A	PDB 6cmn:A
1014	0.1797	0.3105	2.99	3.530	57	5	8	0.07018	0	96	7	79	6	PDB 9b0m:A	PDB 6exx:A
1015	0.1794	0.2936	2.336	3.365	66	5	7	0.09091	0	96	7	112	6	PDB 9b0m:A	PDB 2pii:A
1016	0.1793	0.08572	2.486	3.618	62	5	9	0.08065	0	96	7	91	6	PDB 9b0m:A	PDB 3ucz:P
1017	0.1791	0.1848	2.752	3.353	59	5	10	0.1017	0	96	7	90	6	PDB 9b0m:A	PDB 3l3c:B
1018	0.1791	0.1605	2.699	3.523	60	5	8	0.08333	0	96	7	88	6	PDB 9b0m:A	PDB 6xh3:A
1019	0.1789	0.5509	2.232	3.651	70	5	8	0.1	0	96	7	115	6	PDB 9b0m:A	PDB 3ncq:C
1020	0.1788	0.6646	2.792	2.806	55	5	4	0.1273	0	96	7	94	6	PDB 9b0m:A	PDB 3jst:B
1021	0.1786	0.2406	2.082	3.337	61	5	5	0.1639	0	96	7	97	7	PDB 9b0m:A	PDB 2epi:C
1022	0.1786	0.001083	1.518	4.143	72	5	7	0.08333	0	96	7	104	7	PDB 9b0m:A	PDB 7w6e:B
1023	0.1786	0.5962	2.885	2.749	57	5	3	0.08772	0	96	7	103	6	PDB 9b0m:A	PDB 1f93:A
1024	0.1785	0.2106	2.805	3.328	58	5	10	0.1034	0	96	7	88	6	PDB 9b0m:A	PDB 3g8t:D
1025	0.1776	0.6784	2.805	2.550	55	5	5	0.1091	0	96	7	103	6	PDB 9b0m:A	PDB 2v6u:A
1026	0.1776	0.1873	2.752	3.647	62	5	9	0.08065	0	96	7	91	6	PDB 9b0m:A	PDB 8gxc:D
1027	0.1774	0.07454	1.748	3.807	67	5	6	0.08955	0	96	7	101	5	PDB 9b0m:A	PDB 7qri:B
1028	0.1768	0.139	2.646	3.461	60	5	9	0.08333	0	96	7	91	6	PDB 9b0m:A	PDB 6laz:C
1029	0.1763	0.0833	2.486	3.469	60	5	9	0.08333	0	96	7	91	6	PDB 9b0m:A	PDB 6f4h:A
1030	0.1763	0.1223	2.606	3.504	61	5	8	0.08197	0	96	7	93	6	PDB 9b0m:A	PDB 6xh2:A
1031	0.1763	0.001731	1.601	3.706	57	5	5	0.1754	0	96	7	76	6	PDB 9b0m:A	PDB 4ce4:l
1032	0.1763	0.0137	1.784	4.007	71	5	4	0.1127	0	96	7	107	7	PDB 9b0m:A	PDB 5xzq:L
1033	0.1759	0.5962	2.885	2.557	54	5	3	0.1667	0	96	7	100	6	PDB 9b0m:A	PDB 4wil:A
1034	0.1755	0.00959	1.725	3.814	69	5	7	0.1014	0	96	7	108	7	PDB 9b0m:A	PDB 5xzq:C
1035	0.1755	0.2335	2.858	3.518	61	5	8	0.09836	0	96	7	93	6	PDB 9b0m:A	PDB 5ddq:D
1036	0.1754	0.7325	2.872	2.738	55	5	4	0.1273	0	96	7	98	6	PDB 9b0m:A	PDB 2v6s:B
1037	0.1754	0.1418	2.646	3.383	59	5	10	0.08475	0	96	7	91	6	PDB 9b0m:A	PDB 3iwn:C
1038	0.1753	0.2688	2.911	3.520	60	5	8	0.1	0	96	7	90	6	PDB 9b0m:A	PDB 3l3c:D
1039	0.1753	0.0868	2.218	3.813	67	5	6	0.07463	0	96	7	102	7	PDB 9b0m:A	PDB 7bl5:6
1040	0.1748	0.3201	3.003	3.430	60	5	9	0.08333	0	96	7	93	7	PDB 9b0m:A	PDB 6lax:E
1041	0.1747	0.1361	2.646	3.395	59	5	8	0.08475	0	96	7	91	6	PDB 9b0m:A	PDB 6laz:E
1042	0.1745	0.08124	1.775	3.764	66	5	6	0.09091	0	96	7	101	5	PDB 9b0m:A	PDB 7qri:A
1043	0.1744	0.4783	2.127	3.680	59	5	6	0.1356	0	96	7	83	6	PDB 9b0m:A	PDB 2zbc:A
1044	0.174	0.2157	2.819	3.411	60	5	9	0.08333	0	96	7	94	6	PDB 9b0m:A	PDB 5ddr:C
1045	0.1737	0.01008	1.74	3.907	65	6	5	0.07692	0	96	7	94	7	PDB 9b0m:A	PDB 5b0b:D
1046	0.1734	0.01203	1.829	3.451	57	6	4	0.03509	0	96	7	84	7	PDB 9b0m:A	PDB 4rwx:B
1047	0.1733	0.1109	1.963	4.382	70	5	5	0.08571	0	96	7	94	6	PDB 9b0m:A	PDB 3znj:Z
1048	0.173	0.09315	2.262	3.637	65	6	6	0.04615	0	96	7	103	7	PDB 9b0m:A	PDB 1y9o:A
1049	0.1729	0.1738	2.726	3.459	59	5	9	0.08475	0	96	7	90	6	PDB 9b0m:A	PDB 3l3c:C
1050	0.1727	0.6544	2.779	2.817	55	5	4	0.1273	0	96	7	97	6	PDB 9b0m:A	PDB 3jst:A
1051	0.1724	0.1611	2.699	3.570	60	5	9	0.08333	0	96	7	90	6	PDB 9b0m:A	PDB 3l3c:A
1052	0.1724	0.004248	1.504	4.527	80	5	5	0.0625	0	96	7	118	7	PDB 9b0m:A	PDB 2czv:C
1053	0.1719	0.0005629	1.896	3.784	61	5	4	0.08197	0	96	7	87	7	PDB 9b0m:A	PDB 1jem:A
1054	0.1719	0.5531	2.832	2.689	54	5	4	0.1481	0	96	7	98	6	PDB 9b0m:A	PDB 4wil:B
1055	0.1717	0.119	2.739	3.688	60	5	3	0.1333	0	96	7	87	7	PDB 9b0m:A	PDB 1fu0:A
1056	0.1712	0.07951	2.473	3.489	59	5	9	0.08475	0	96	7	90	6	PDB 9b0m:A	PDB 6f4g:E
1057	0.171	0.06724	1.815	4.242	57	5	4	0.05263	0	96	7	66	5	PDB 9b0m:A	PDB 3bkf:A
1058	0.1709	0.002072	1.548	3.953	68	5	7	0.1029	0	96	7	103	7	PDB 9b0m:A	PDB 5xzq:F
1059	0.1706	0.116	2.739	3.707	60	5	3	0.1333	0	96	7	87	7	PDB 9b0m:A	PDB 1fu0:B
1060	0.1706	0.2658	2.911	3.604	59	5	9	0.1356	0	96	7	87	6	PDB 9b0m:A	PDB 6exn:Y
1061	0.1702	0.266	2.911	3.446	60	5	9	0.08333	0	96	7	95	7	PDB 9b0m:A	PDB 2oj3:A

1062	0.1701	0.003639	1.607	3.911	69	5	8	0.1014	0	96	7	108	7	PDB 9b0m:A	PDB 5xzq:D
1063	0.1687	0.07061	2.593	3.706	63	5	6	0.09524	0	96	7	97	7	PDB 9b0m:A	PDB 7dwh:D
1064	0.1687	0.2776	2.232	3.398	58	5	4	0.1207	0	96	7	91	5	PDB 9b0m:A	PDB 2jz5:A
1065	0.1686	0.09166	2.513	3.438	59	5	9	0.08475	0	96	7	93	6	PDB 9b0m:A	PDB 5ddp:C
1066	0.1668	0.01012	1.74	4.037	71	5	5	0.09859	0	96	7	112	7	PDB 9b0m:A	PDB 1q53:A
1067	0.166	0.2088	2.097	3.855	52	5	4	0.05769	0	96	7	64	5	PDB 9b0m:A	PDB 4q2l:A
1068	0.166	0.00289	1.896	3.559	61	5	7	0.06557	0	96	7	97	7	PDB 9b0m:A	PDB 5fj4:B
1069	0.1654	0.001419	1.615	4.070	63	5	5	0.09524	0	96	7	88	7	PDB 9b0m:A	PDB 1qr5:A
1070	0.1651	0.6559	2.779	2.689	54	5	4	0.1296	0	96	7	102	6	PDB 9b0m:A	PDB 2v6s:A
1071	0.1649	0.04393	2.326	3.439	59	5	9	0.05085	0	96	7	95	7	PDB 9b0m:A	PDB 1m5o:F
1072	0.1629	0.09016	1.903	4.341	66	5	4	0.1212	0	96	7	90	6	PDB 9b0m:A	PDB 4fpi:E
1073	0.1628	0.467	2.112	4.019	62	5	6	0.08065	0	96	7	88	6	PDB 9b0m:A	PDB 2err:A
1074	0.1624	0.1861	1.978	3.543	56	5	5	0.1071	0	96	7	84	7	PDB 9b0m:A	PDB 7ei1:U
1075	0.1622	0.04222	1.23	4.189	61	5	5	0.06557	0	96	7	81	6	PDB 9b0m:A	PDB 5iqq:D
1076	0.1622	0.09705	1.918	4.218	66	5	5	0.1061	0	96	7	94	6	PDB 9b0m:A	PDB 3znj:4
1077	0.162	0.00343	1.33	4.169	60	5	5	0.1333	0	96	7	79	6	PDB 9b0m:A	PDB 3r2d:K
1078	0.1619	0.2136	2.805	3.910	59	5	8	0.1017	0	96	7	83	6	PDB 9b0m:A	PDB 5w0g:A
1079	0.161	0.01064	2.057	4.148	60	5	5	0.1333	0	96	7	80	6	PDB 9b0m:A	PDB 3r2c:K
1080	0.1608	0.2273	2.832	3.890	60	5	8	0.1	0	96	7	87	6	PDB 9b0m:A	PDB 2hzc:A
1081	0.1607	0.1843	2.752	3.657	61	5	8	0.06557	0	96	7	97	7	PDB 9b0m:A	PDB 5fj4:F
1082	0.1607	0.0614	1.799	4.219	65	5	5	0.1231	0	96	7	92	6	PDB 9b0m:A	PDB 3znj:3
1083	0.1598	0.06692	2.419	3.637	60	5	9	0.08333	0	96	7	95	6	PDB 9b0m:A	PDB 3bo4:A
1084	0.1597	0.05449	1.769	4.273	65	5	5	0.1231	0	96	7	91	6	PDB 9b0m:A	PDB 3znu:I
1085	0.1597	0.06237	1.799	4.175	64	5	5	0.125	0	96	7	91	6	PDB 9b0m:A	PDB 3zo7:F
1086	0.1588	0.0699	1.829	3.991	63	5	5	0.127	0	96	7	94	6	PDB 9b0m:A	PDB 4fpi:T
1087	0.1576	0.2397	2.247	3.337	64	5	5	0.1406	0	96	7	121	6	PDB 9b0m:A	PDB 2lxf:A
1088	0.1575	0.03582	2.473	3.308	57	5	8	0.05263	0	96	7	97	7	PDB 9b0m:A	PDB 7dwh:C
1089	0.1574	0.07302	1.844	4.215	65	5	5	0.1231	0	96	7	94	6	PDB 9b0m:A	PDB 4fpi:H
1090	0.1574	0.4229	2.459	3.493	62	5	3	0.1129	0	96	7	108	5	PDB 9b0m:A	PDB 2khd:A
1091	0.1574	0.05331	1.769	4.216	65	5	5	0.1231	0	96	7	94	6	PDB 9b0m:A	PDB 3znj:8
1092	0.1572	0.05449	1.769	4.316	66	5	4	0.1212	0	96	7	94	6	PDB 9b0m:A	PDB 3znj:S
1093	0.1567	0.05246	1.769	4.234	64	5	5	0.1094	0	96	7	91	6	PDB 9b0m:A	PDB 3znj:9
1094	0.1567	0.06428	1.814	4.230	65	5	5	0.1231	0	96	7	94	6	PDB 9b0m:A	PDB 3znj:F
1095	0.1563	0.03815	1.695	4.239	65	5	5	0.1231	0	96	7	94	6	PDB 9b0m:A	PDB 3znj:N
1096	0.1562	0.06237	1.799	4.239	65	5	5	0.1231	0	96	7	94	6	PDB 9b0m:A	PDB 3znj:6
1097	0.1561	0.07302	1.844	4.045	63	5	5	0.127	0	96	7	94	6	PDB 9b0m:A	PDB 3zo7:C
1098	0.1561	0.0878	1.888	4.045	63	5	5	0.127	0	96	7	94	6	PDB 9b0m:A	PDB 4fpi:M
1099	0.1559	0.06428	1.814	4.147	64	5	5	0.125	0	96	7	94	6	PDB 9b0m:A	PDB 3zo7:G
1100	0.1557	0.05713	1.784	4.250	65	5	5	0.1231	0	96	7	94	6	PDB 9b0m:A	PDB 3znj:d
1101	0.1555	0.05165	1.754	4.157	64	5	5	0.125	0	96	7	94	6	PDB 9b0m:A	PDB 3znu:A
1102	0.1553	0.07798	1.859	4.258	65	5	5	0.1231	0	96	7	94	6	PDB 9b0m:A	PDB 4fpi:K
1103	0.1551	0.05421	1.769	4.262	65	5	5	0.1231	0	96	7	94	6	PDB 9b0m:A	PDB 3znj:E
1104	0.1551	0.0614	1.799	4.065	63	5	5	0.127	0	96	7	94	6	PDB 9b0m:A	PDB 3znj:J
1105	0.1549	0.06428	1.814	4.266	65	5	5	0.1231	0	96	7	94	6	PDB 9b0m:A	PDB 3znj:P
1106	0.1549	0.07303	1.844	4.168	64	5	5	0.125	0	96	7	94	6	PDB 9b0m:A	PDB 4fpi:J
1107	0.1547	0.04367	1.725	4.270	65	5	5	0.1231	0	96	7	94	6	PDB 9b0m:A	PDB 4fpi:A
1108	0.1547	0.05741	1.784	4.073	63	5	5	0.127	0	96	7	94	6	PDB 9b0m:A	PDB 3znj:G
1109	0.1545	0.0008774	1.201	4.278	60	5	5	0.1	0	96	7	80	6	PDB 9b0m:A	PDB 2kvq:E
1110	0.1545	0.04368	1.725	4.177	64	5	5	0.125	0	96	7	94	6	PDB 9b0m:A	PDB 3znu:G
1111	0.1544	0.002841	1.315	4.214	59	5	5	0.1356	0	96	7	79	6	PDB 9b0m:A	PDB 3r2c:J
1112	0.1543	0.05449	1.769	4.180	64	5	5	0.125	0	96	7	94	6	PDB 9b0m:A	PDB 4fpi:P
1113	0.1543	0.05165	1.754	4.181	64	5	5	0.125	0	96	7	94	6	PDB 9b0m:A	PDB 3znj:L
1114	0.1542	0.05078	1.754	4.183	64	5	5	0.125	0	96	7	94	6	PDB 9b0m:A	PDB 3znj:T
1115	0.1541	0.09046	1.431	4.058	60	5	5	0.08333	0	96	7	86	6	PDB 9b0m:A	PDB 5bjr:A
1116	0.154	0.04969	1.754	4.187	64	5	5	0.125	0	96	7	94	6	PDB 9b0m:A	PDB 4fpi:D
1117	0.1537	0.002956	1.33	3.771	62	5	6	0.06452	0	96	7	101	6	PDB 9b0m:A	PDB 1y0h:A
1118	0.1536	0.04367	1.725	4.195	64	5	5	0.125	0	96	7	94	6	PDB 9b0m:A	PDB 4fpi:O
1119	0.1535	0.07441	1.844	4.196	64	5	5	0.125	0	96	7	94	6	PDB 9b0m:A	PDB 4fpi:G
1120	0.1535	0.08165	1.873	4.098	63	5	5	0.127	0	96	7	94	6	PDB 9b0m:A	PDB 4fpi:F
1121	0.1534	0.06991	1.829	4.199	64	5	5	0.125	0	96	7	94	6	PDB 9b0m:A	PDB 4fpi:C
1122	0.1534	0.07302	1.976	4.460	67	5	6	0.1194	0	96	7	95	6	PDB 9b0m:A	PDB 6xav:L
1123	0.1533	0.01268	1.504	4.202	64	5	5	0.1094	0	96	7	94	6	PDB 9b0m:A	PDB 4fpi:U
1124	0.1531	0.03615	1.68	4.206	64	5	5	0.125	0	96	7	94	6	PDB 9b0m:A	PDB 3znj:M
1125	0.1531	0.04048	1.71	4.305	65	5	5	0.1231	0	96	7	94	6	PDB 9b0m:A	PDB 4fpi:R
1126	0.1526	0.003622	1.909	4.465	57	5	9	0.1053	0	96	7	69	6	PDB 9b0m:A	PDB 4wwu:D
1127	0.1525	0.05331	1.769	4.252	64	5	5	0.1094	0	96	7	93	6	PDB 9b0m:A	PDB 3znj:2
1128	0.1524	0.05449	1.769	4.219	64	5	5	0.125	0	96	7	94	6	PDB 9b0m:A	PDB 4fpi:N
1129	0.1521	0.1052	1.948	3.713	58	5	6	0.1207	0	96	7	91	6	PDB 9b0m:A	PDB 3znj:Y
1130	0.1516	0.06657	1.814	4.236	64	5	5	0.125	0	96	7	94	6	PDB 9b0m:A	PDB 3znj:C
1131	0.1516	0.06428	1.814	4.236	64	5	5	0.125	0	96	7	94	6	PDB 9b0m:A	PDB 3znu:C
1132	0.1516	0.06112	1.799	4.237	64	5	5	0.125	0	96	7	94	6	PDB 9b0m:A	PDB 3znj:I
1133	0.1505	0.02643	1.621	4.259	64	5	5	0.1094	0	96	7	94	6	PDB 9b0m:A	PDB 3znj:1
1134	0.1502	0.04367	1.725	4.166	63	5	5	0.1111	0	96	7	94	6	PDB 9b0m:A	PDB 3znj:c
1135	0.1501	0.09016	1.903	3.758	59	5	6	0.1186	0	96	7	94	6	PDB 9b0m:A	PDB 3znj:R
1136	0.1501	0.007152	2.003	3.974	56	5	7	0.1429	0	96	7	79	6	PDB 9b0m:A	PDB 7s7b:C
1137	0.149	6.212e-07	1.048	4.257	64	5	7	0.07812	0	96	7	95	7	PDB 9b0m:A	PDB 4hl9:I
1138	0.1487	0.02624	1.621	4.198	63	5	5	0.1111	0	96	7	94	6	PDB 9b0m:A	PDB 4fpi:L
1139	0.1486	0.09182	2.911	3.704	55	5	7	0.1091	0	96	7	84	7	PDB 9b0m:A	PDB 7eb1:A
1140	0.1481	0.04703	1.244	4.469	69	5	6	0.04348	0	96	7	104	6	PDB 9b0m:A	PDB 6zvh:U
1141	0.1479	0.1109	1.963	3.908	60	5	6	0.1167	0	96	7	94	6	PDB 9b0m:A	PDB 3znj:K
1142	0.1465	1.718e-07	0.6267	4.039	53	5	4	0.1321	0	96	7	71	7	PDB 9b0m:A	PDB 1scj:B
1143	0.1461	0.02495	2.205	3.734	63	5	7	0.1587	0	96	7	111	7	PDB 9b0m:A	PDB 5ool:u
1144	0.146	0.01327	1.842	3.884	60	5	3	0.01667	0	96	7	96	7	PDB 9b0m:A	PDB 6nlu:B
1145	0.1441	8.501e-06	1.201	4.376	69	5	5	0.05797	0	96	7	110	7	PDB 9b0m:A	PDB 3hf5:C
1146	0.1439	6.096e-05	1.201	3.864	60	5	5	0.15	0	96	7	98	7	PDB 9b0m:A	PDB 3ced:B
1147	0.1439	0.155	2.686	3.647	52	5	7	0.1154	0	96	7	79	6	PDB 9b0m:A	PDB 3hi9:A
1148	0.1439	6.404e-05	1.216	3.864	60	5	5	0.15	0	96	7	98	7	PDB 9b0m:A	PDB 3ced:A
1149	0.1438	0.006908	1.431	4.262	57	5	6	0.05263	0	96	7	78	6	PDB 9b0m:A	PDB 1no8:A
1150	0.1428	0.116	2.593	3.274	52	5	7	0.1154	0	96	7	90	6	PDB 9b0m:A	PDB 2dnp:A
1151	0.1423	0.000371	1.33	3.796	59	5	5	0.1525	0	96	7	98	7	PDB 9b0m:A	PDB 3ced:C
1152	0.1418	0.02377	1.117	4.218	58	5	6	0.1034	0	96	7	83	6	PDB 9b0m:A	PDB 5iqq:A
1153	0.141	0.05849	1.695	4.078	66	5	4	0.1061	0	96	7	113	5	PDB 9b0m:A	PDB 6bi6:A
1154	0.1409	0.04064	1.216	4.172	56	5	6	0.08929	0	96	7	79	6	PDB 9b0m:A	PDB 7s7c:C
1155	0.1408	0.02665	1.769	4.340	64	5	6	0.04688	0	96	7	98	7	PDB 9b0m:A	PDB 6c6u:N
1156	0.139	0.9888	3.858	3.272	51	5	4	0.1176	0	96	7	89	6	PDB 9b0m:A	PDB 2n3l:A
1157	0.1368	0.09212	2.245	2.888	45	5	4	0.06667	0	96	7	80	7	PDB 9b0m:A	PDB 4olp:D
1158	0.1302	2.547e-13	1.156	3.636	58	5	6	0.05172	0	96	7	109	7	PDB 9b0m:A	PDB 3hds:C

1159	0.1278	0.0197	1.775	4.215	67	5	6	0.04478	0	96	7	123	6	PDB 9b0m:A	PDB 2k06:A
1160	0.1255	0.000648	1.482	5.076	55	5	5	0.1455	0	96	7	65	6	PDB 9b0m:A	PDB 4yn3:B
1161	0.1247	0.528	2.217	3.682	60	5	5	0.1	0	96	7	120	6	PDB 9b0m:A	PDB 8gf6:X
1162	0.1223	0.6042	2.901	3.817	62	5	6	0.08065	0	96	7	125	7	PDB 9b0m:A	PDB 5knw:A
1163	0.1205	0.2658	2.911	4.114	58	5	7	0.1034	0	96	7	101	6	PDB 9b0m:A	PDB 2hvz:A
1164	0.1168	0.001136	1.748	3.950	57	5	4	0.08772	0	96	7	106	6	PDB 9b0m:A	PDB 2kzf:A
1165	0.1155	6.011e-05	1.376	4.340	54	5	4	0.1296	0	96	7	85	7	PDB 9b0m:A	PDB 3bv8:A
1166	0.1115	0.001283	0.8226	3.946	51	5	6	0.03922	0	96	7	89	6	PDB 9b0m:A	PDB 1o51:A
1167	0.1005	0.002058	1.842	3.631	49	5	7	0.102	0	96	7	101	6	PDB 9b0m:A	PDB 5szw:A
1168	0.09769	0.1253	1.504	4.297	61	5	5	0.1311	0	96	7	130	6	PDB 9b0m:A	PDB 2mk4:A
1169	0.04445	0.02307	1.909	4.353	53	5	4	0.1132	0	96	7	212	7	PDB 9b0m:A	PDB 3r8y:E
1170	0.04435	0.004578	1.95	4.393	52	5	5	0.1154	0	96	7	202	7	PDB 9b0m:A	PDB 3r8y:F
1171	0.04432	0.004082	1.936	4.411	52	5	5	0.1154	0	96	7	201	7	PDB 9b0m:A	PDB 3r8y:C
1172	0.04429	0.00425	1.95	4.381	52	5	5	0.1154	0	96	7	203	7	PDB 9b0m:A	PDB 3r8y:D
1173	0.03595	−0	0.1413	7.626	52	5	3	0.09615	0	96	7	105	7	PDB 9b0m:A	PDB 1kpt:B
1174	0.03516	−0	0.1413	7.895	53	5	4	0.07547	0	96	7	105	7	PDB 9b0m:A	PDB 1kpt:A
